# Targeting WDxR motif reprograms immune microenvironment and inhibits hepatocellular carcinoma progression

**DOI:** 10.15252/emmm.202215924

**Published:** 2023-03-22

**Authors:** Heng Zhang, Gang Chen, Xing Feng, Huiwen Song, Lingbing Meng, Yao Fu, Jun Yang, Zhiwen Fan, Youxiang Ding, Zhijie Du, Jianchao Wang, Li Yang, Jun Zhang, Lixia Sun, Zhigang Liu, Zhiyong Zhang, Quanhai Li, Xiangshan Fan

**Affiliations:** ^1^ Department of Thoracic Surgery Renmin Hospital of Wuhan University Wuhan China; ^2^ Department of Histology and Embryology, Xiang Ya School of Medicine Central South University Changsha China; ^3^ Department of Pathology Fujian Medical University Cancer Hospital, Fujian Cancer Hospital Fuzhou China; ^4^ Department of Immunobiology Yale University School of Medicine New Haven CT USA; ^5^ Department of Cardiology Jiading District Central Hospital Affiliated Shanghai University of Medicine & Health Sciences Shanghai China; ^6^ Departments of Cardiology, Beijing Hospital, National Center of Gerontology Chinese Academy of Medical Sciences Beijing China; ^7^ Department of Pathology, The Affiliated Drum Tower Hospital Nanjing University Medical School Nanjing China; ^8^ Nanjing Drum Tower Hospital Clinical College of Traditional Chinese and Western Medicine Nanjing University of Chinese Medicine Nanjing China; ^9^ Institute of Digestive Disease China Three Gorges University Yichang China; ^10^ Department of Gastroenterology Yichang Central People's Hospital Yichang China; ^11^ Shenzhen Qianhai Shekou Free Trade Zone Hospital Shenzhen China; ^12^ Department of Hepatological Surgery The Affiliated Wuhu hospital of ECNU Wuhu China; ^13^ Department of Surgery, Robert‐Wood‐Johnson Medical School University Hospital Rutgers University New Brunswick NJ USA; ^14^ National Center for International Research of Biological Targeting Diagnosis and Therapy Guangxi Medical University Nanning China; ^15^ Cell Therapy Laboratory The First Hospital of Hebei Medical University Shijiazhuang China

**Keywords:** MDSC, PD‐1, TNFα, UVRAG, WDR6, Cancer, Digestive System, Immunology

## Abstract

The WD‐repeat (WDR) family affects carcinogenesis, but its role in the immune microenvironment is poorly characterized. Although functional loss or gain of WDR6 does not markedly change *in vitro* proliferative and invasive capacity of HCC cells, its deficiency in hepa1‐6 cells drastically inhibits the growth and lung metastasis of orthotopically implanted tumors in immune‐competent C57BL/6J mice. Mechanistically, WDR6 targets tumor suppressor UVRAG to the CUL4A‐DDB1‐ROC1 E3 ubiquitin ligase complex through a unique WDxR motif and promotes its degradation. This upregulates chromatin accessibility at the TNFα locus by blocking autophagic degradation of p65, elevates intratumoral myeloid‐derived suppressor cell (MDSC) number, and reduces CD8^+^ T cell infiltration, thereby promoting HCC progression. These immunosuppressive effects are reversed by TNFα blockade. TNFα recruits NF‐κB to activate the transcription of *WDR6*, establishing a WDR6‐TNFα loop. Clinically, the WDR6/UVRAG/NF‐κB pathway is hyperactivated in HCC, predicting a poor prognosis. Importantly, a WDxR‐like peptide disrupts the WDR6/UVRAG complex and enhances the efficiency of anti‐PD‐L1 against HCC with WDR6 dysregulation.

## Graphical abstract

The WD‐repeat (WDR) family affects carcinogenesis, but its role in immune microenvironment is poorly characterized. This study reports the cellular and molecular effects of targeting WDR6 on HCC.

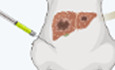

**The paper explained**

**Problem**

The bad prognosis and high mortality of hepatocellular carcinoma (HCC) are ascribed to a high recurrence rate postresection and the absence of early diagnosis tools. Identifying novel targets to inhibit HCC progression remains an important need. Here, we investigated the role of WDR6 in HCC.

**Results**

WDR6 deficiency inhibits HCC growth and metastasis in immune‐competent mice only. WDR6 degraded UVRAG by recruiting CUL4A‐DDB1‐ROC1 E3 ligase complex through its unique WDxR motif, which upregulated p65‐induced TNFα, elevated intratumoral MDSC number, and reduces CD8^+^ T cell infiltration, thereby promoting HCC progression. These immunosuppressive effects were reversed by TNFα blockade. TNFα in turn recruited NF‐κB to activate WDR6 transcription, establishing a WDR6‐TNFα loop. Clinically, the WDR6/UVRAG/NF‐κB pathway was hyperactivated in HCC, and predicted a poor prognosis. Importantly, a WDxR‐like peptide enhanced the efficiency of anti‐PD‐L1 against HCC.

**Impact**

Our findings demonstrate the therapeutic potential of immune‐modulation to suppress HCC with WDR6 dysregulation.

## Introduction

The poor prognosis and high mortality of hepatocellular carcinoma (HCC) patients are ascribed to a high recurrence rate postresection and the absence of early diagnosis tools (Ma *et al*, 2019). Recent studies demonstrate a central role of the tumor immune microenvironment (TIME) in HCC development (Dituri *et al*, 2019). Fundamentally, the intricate crosstalk between tumor and immune cells produces a cytokine‐rich milieu conducive to the growth and metastasis of HCC (Keenan *et al*, 2019). Therefore, immunotherapy may make a big difference for patients with HCC. However, the key points addressing these issues are still lacking in HCC.

WD‐repeat 6 (WDR6) protein is a less well‐explored member of the WDR superfamily despite its ubiquitous expression (Chiba *et al*, 2009). Chromosomal arm 15q21 in which *WDR6* is located often harbors putative progression‐associated genes in different tumors, suggesting a relationship between *WDR6* and carcinogenesis (Li *et al*, 2000). Recently, several groups have observed its dysregulation in different tumors, including HCC (Yafune *et al*, 2013; Savci‐Heijink *et al*, 2019). Thus, WDR6 may be an unknown regulator critical to tumor progression.

The goal of this study is to explore the impact of WDR6 and the underlying mechanism of its involvement in HCC. We demonstrate that WDR6 promotes HCC progression by enhancing ubiquitination‐dependent degradation of tumor suppressor UV radiation resistance‐associated gene (UVRAG), thereby fostering an immunosuppressive and prometastatic tumor microenvironment. This novel finding suggests that the WDR6/UVRAG axis may be a target for HCC treatments.

## Results

### WDR6 level is positively correlated to the malignancy of HCC and predicts a poor prognosis

**Figure 1:**
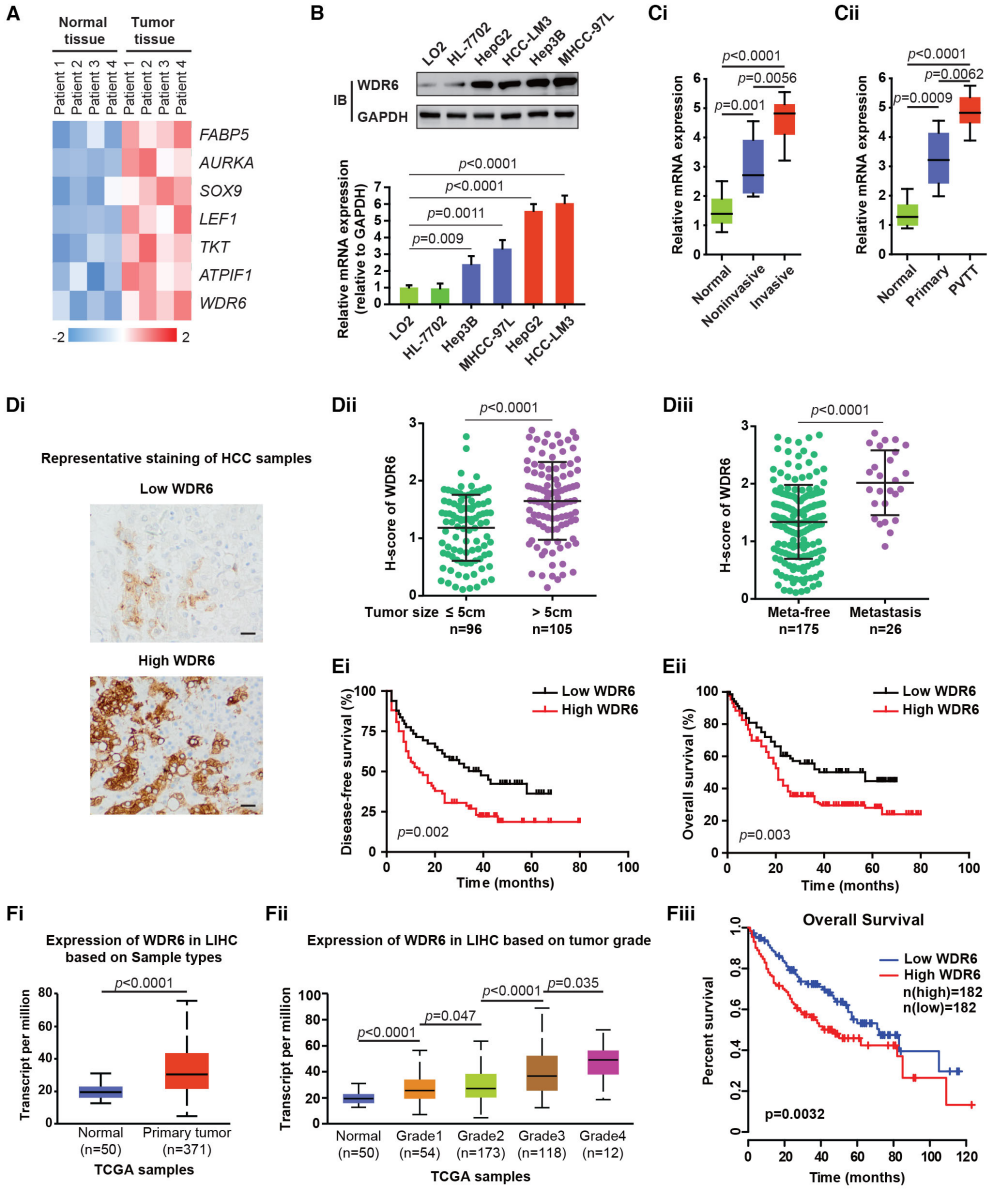
Higher levels of WDR6 in HCC tissues indicate a worse prognosis of patients and metastasis AHeatmap of altered genes in four pairs of HCC and adjacent normal tissues from patients.BAnalyzing WDR6 expression in the normal and HCC cell lines.CiThe levels of WDR6 were determined and normalized against GAPDH in three groups. Normal, *n* = 21; Noninvasive, *n* = 15; Invasive, *n* = 8.CiiThe levels of WDR6 in 10 pairs of patients' tissues.DiRepresentative IHC staining of WDR6 in clinical HCC samples (*n* = 201). Scale bars: 100 μm.Dii, DiiiWDR6 expression was analyzed based on the following parameters: tumor size and distant metastasis (*n* = 201).Ei, EiiThe DFS and OS assays of HCC patients (*n* = 201) stratified by WDR6 expression.Fi–FiiiAnalysis of WDR6 using TCGA LIHC data. (Fi) and (Fiii) were plotted with GEPIA analyzing tool, and (Fii) were analyzed by UALCAN analyzing tool. Data information: All results are representative data generated from three independent experiments. Data are presented as mean ± SD. Unpaired two‐tailed Student's *t*‐test (B–Diii, Fi, Fii) were used for statistical analysis, and long‐rank (Mantel‐cox) test for survival comparison (Ei, Eii, Fiii). Source data are available online for this figure.

To screen potentially novel biomarkers in HCC, four patients' HCC tissues and matched normal controls were subjected to RNA‐seq. Among seven representative genes whose levels were significantly upregulated in tumor tissues (Fig 1A and Appendix Table S1), we focused on WDR6 based on three reasons: (i) WDR6 is rarely reported in tumors, and its expression level was higher in our tumor tissue samples (fold change 3.45, *P*‐value 0.003891); (ii) several members of the WDR family regulate carcinogenesis (Li *et al*, 2000); (iii) the dysregulation of the six additional genes identified by RNASeq have already been reported to be associated with HCC pathogenesis. For example, we previously reported the effects of ATPIF1 on HCC (Song *et al*, 2014).

Interestingly, the levels of WDR6 protein and mRNA progressively increased from normal hepatocyte cells to highly metastatic tumor cells (Fig 1B). Clinically, the mRNA levels of WDR6 in invasive samples were markedly higher than those in noninvasive tumors or control samples (Fig 1Ci). Similar data were obtained in HCC patients with portal vein tumor thrombus (PVTT) usually related to frequent recurrence and intrahepatic metastasis (Yu & Park, 2016) (Fig 1Cii). Therefore, WDR6 may be involved in HCC aggressiveness.

Furthermore, immunohistochemical staining (IHC) using clinical HCC samples (*n* = 201) revealed that enhanced WDR6 expression levels were significantly related to increased tumor size, vascular invasion, and distant metastasis (Fig 1D, Appendix Table S2). Higher WDR6 levels predicted worse DFS or OS (Fig 1E). Similar findings were observed in the cohort of HCC patients from the TCGA LIHC dataset (Fig 1F). Collectively, WDR6 may be an unrecognized marker of poor prognosis in HCC.

### WDR6 significantly promotes HCC growth and metastasis possibly via reprogramming the tumor immune microenvironment

**Figure 2:**
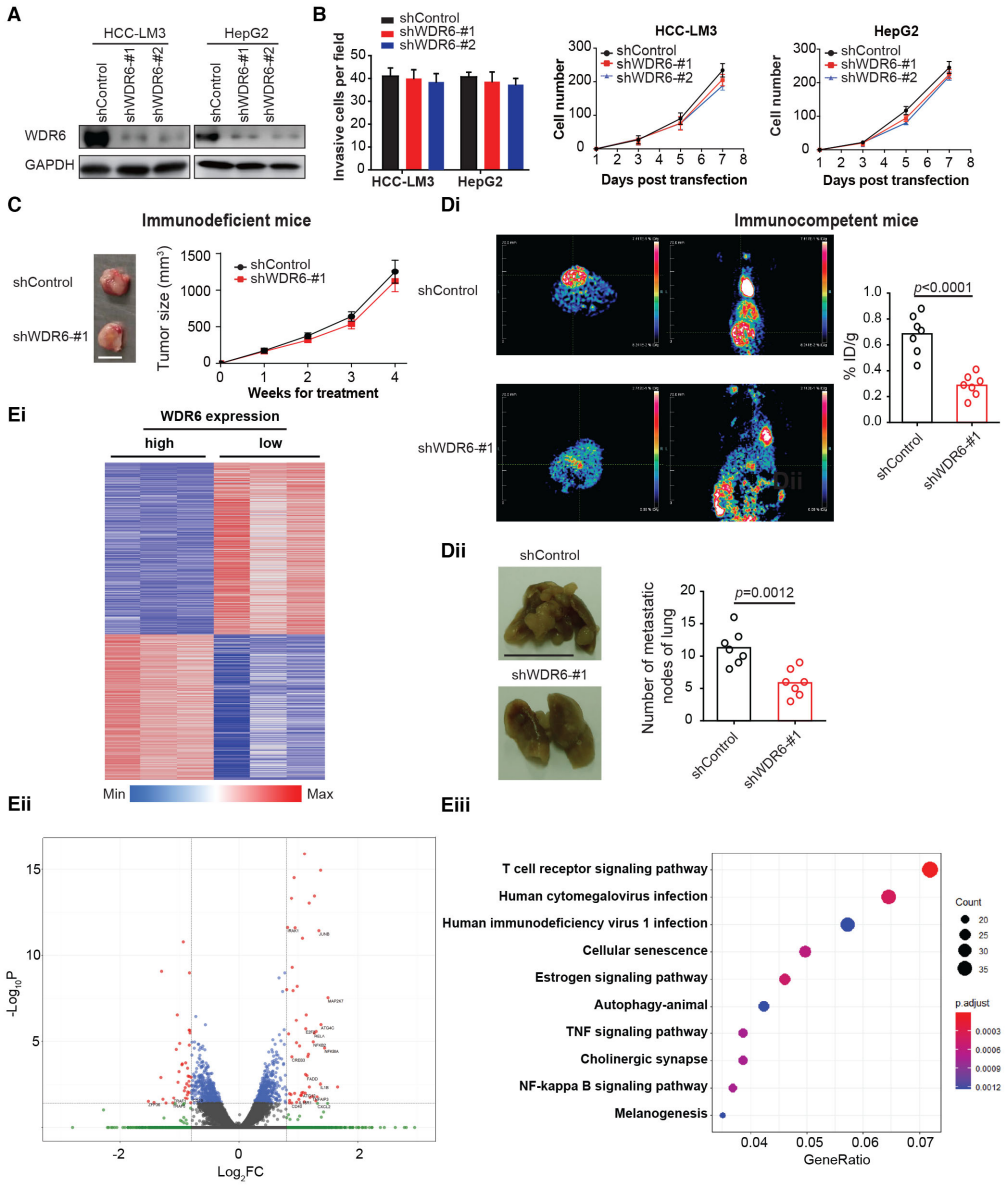
WDR6 stimulates HCC development possibly through affecting TIME AWestern blots confirmed *WDR6* knockdown in HCC cells transduced with *shWDR6* or *shcontrol* lentiviral vector.BThe effects of *WDR6* silencing on the invasive and proliferative ability of HCC cells, respectively. Left panel: HCC‐LM3: *P* = 0.912 shControl versus shWDR6‐#1, *P* = 0.889 shControl versus shWDR6‐#2; HepG2: *P* = 0.826 shControl versus shWDR6‐#1, *P* = 0.796 shControl versus shWDR6‐#2; right panel: HCC‐LM3: *P* = 0.856 shControl versus shWDR6‐#1, *P* = 0.814 shControl versus shWDR6‐#2; HepG2: *P* = 0.726 shControl versus shWDR6‐#1, *P* = 0.745 shControl versus shWDR6‐#2.CHCC‐LM3 cells transfected with or without *shWDR6* were subcutaneously implanted into nude mice (*n* = 6/group, scale bar = 1 cm). Representative images and growth curve of the HCC‐LM3 subcutaneous tumors were shown. *P* = 0.903.Di, DiiHepa1‐6 cells transfected with or without *shWDR6* were orthotopically implanted into C57BL/6J mouse (*n* = 7/group, scale bar = 1 cm). PET‐CT quantification of tumors and lung metastasis were examined.EiThe heatmap displaying differentially expressed genes between WDR6 high and low HCC tumor tissues.EiiVolcano plot of differential expressed genes between WDR6 high and low HCC tumor tissues. *x* axis indicates the Log_2_Fold change value (cutoff lines represent fold change > 1.8), and *y* axis shows −Log_10_ *P* values (cutoff line represents *P*‐value < 0.05), gray indicates genes with no significant differences. Highlighted genes were shown.EiiiStatistical scatter plot of differential gene pathway enrichment for clinical HCC tissues with WDR6 high or low expression. Data information: All results are representative data generated from three independent experiments. Data are presented as mean ± SD. One‐way ANOVA (B), two‐way ANOVA (B, C), and unpaired two‐tailed Student's *t*‐test (Di, Dii) were used for statistical analysis. Source data are available online for this figure.

Next, we tried to clarify the function of WDR6 in HCC. We found that *in vitro WDR6* knockdown by small hairpin RNAs (shRNAs) or overexpression did not markedly decrease or increase the malignant characteristics of HCC cells, respectively (Fig 2A and B, Appendix Fig S1A). *In vivo*, *WDR6* knockdown in HCC‐LM3 cells barely reduced the growth of subcutaneous tumors in nude mice (Fig 2C). Similarly, subcutaneously implanted MHCC‐97L cells with *WDR6* overexpression had no significant effects (Appendix Fig S1B). Given that nude mice are immune‐deficient (Rossa & D'Silva, 2019), we orthotopically implanted hepa1‐6 cells with *WDR6* knockdown (Appendix Fig S1C) into immune‐competent C57BL/6J (Li *et al*, 2019). It was observed that *WDR6* deficiency drastically repressed the growth and metastasis of HCC (Fig 2Di and Dii). These findings suggest that WDR6 promotes the malignancy state of HCC possibly by reprogramming the tumor immune microenvironment (TIME) rather than directly affecting HCC cells themselves.

To identify the possible mechanism of action of WDR6 in HCC development, we compared transcriptomes between clinical HCC tissues with WDR6 high or low expression. This RNA sequencing uncovered 590 downregulated and 667 upregulated genes (Fig 2Ei, Appendix Table S3). Some of the significantly altered genes were highlighted in the volcano plot, including RELA, NFKB2, NFKBIA, E2F2, CD40, IL1B, IL18R1, TRAF1, TRAF5, TNFIP3, ATG12, Beclin1, and ATG4C (Fig 2Eii). KEGG analysis revealed that the largest number of differentially expressed genes (DEGs) were components of the T cell receptor signaling pathway, 30 out of 39 of which were upregulated (Fig 2Eiii). Thus, we hypothesized that WDR6 might enhance HCC progression by reprogramming the TIME.

### WDR6 promotes a MDSC‐abundant TIME in HCC

**Figure 3:**
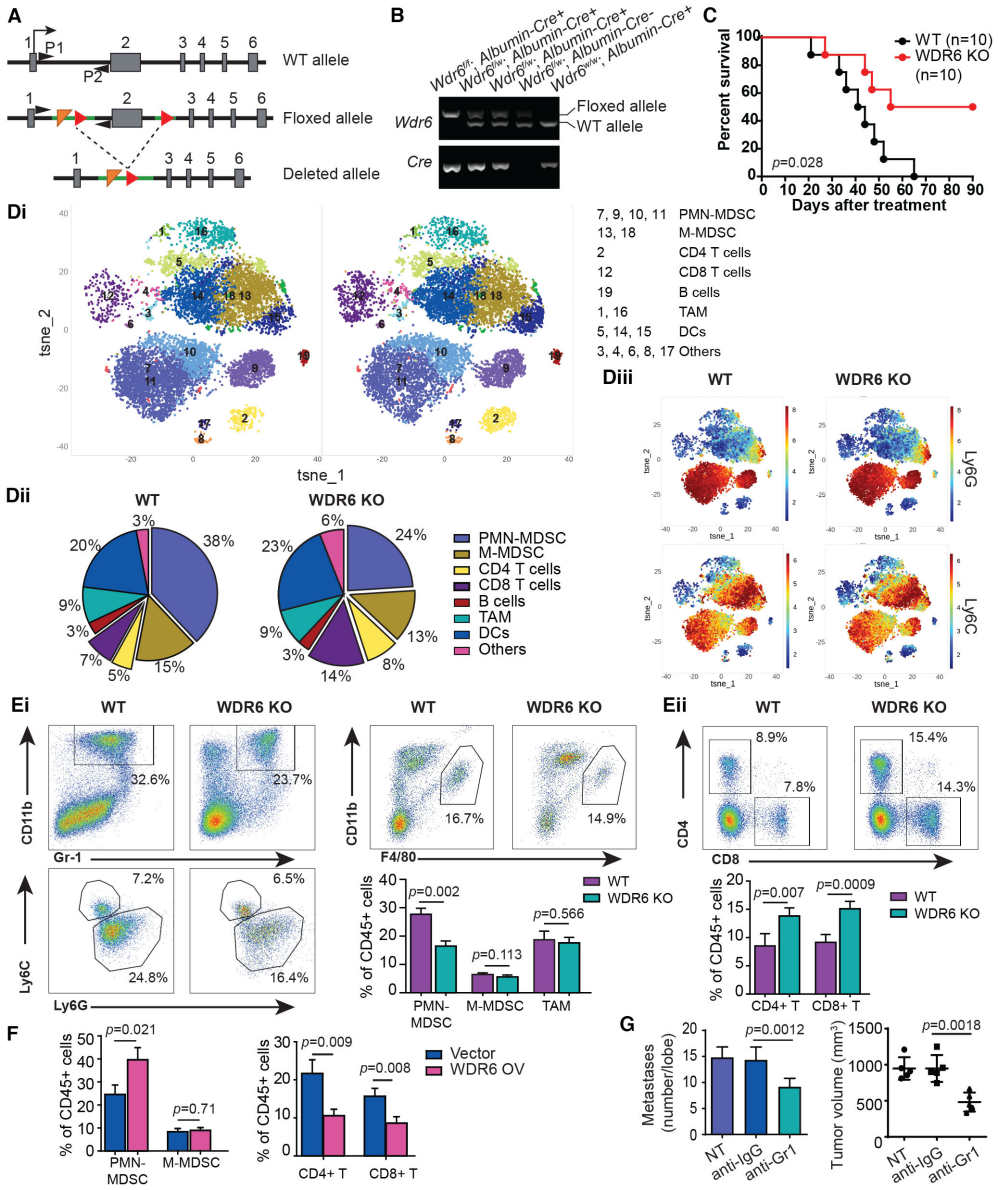
WDR6 promotes an immune‐suppressive microenvironment in HCC AEstablishing of *Albumin‐Cre* ^/+^; *Wdr6* ^*f/f*^ mice. Schematic genome map of wild‐type, floxed, and deleted alleles. Loxp sites (red triangles) were inserted in intron 2 and 3. Liver‐specific cre recombination (*Albumin‐Cre*) results in exon 2 deletion and *Wdr6* gene silence. Genotyping primers (P1 and P2, black triangles) were used.BRepresentative PCR result of genotyping.CThe survival plot of *Albumin‐Cre* ^/+^; *Wdr6* ^*f/f*^ mice with DEN‐induced HCC.Di–DiiiCyTOF analysis of DEN‐induced HCC in WT and *WDR6‐KO* mice. TILs were collected and analyzed using CyTOF (10,000 cells from each). (Di) Cellular distribution and clustering for WT and *WDR6‐KO* samples were colored and defined with PhenoGraph and tSNE1‐2. (Dii) Pie charts indicated the distribution of WT and *WDR6‐KO* TILs in clusters. (Diii) PhenoGraph‐based visualization, colored based on Ly6G and Ly6C expression, demonstrated cell clustering and varied scaling on a tSNE plot.Ei, EiiFlow cytometry analysis shows decreased PMN‐MDSCs (Ei) and increased CD8^+^ T cells (Eii) in DEN‐induced HCC using *WDR6‐KO* mice (*n* = 6/group), while no significant difference in M‐MDSC and TAM subsets (Ei).FFlow cytometric analysis of MDSC and T cells in tumors from BALB/c mice with H22‐WDR6 or empty vector cells orthotopically implantation (*n* = 6/group).Gα‐Gr1 antibody injection markedly decreased the tumor volume and lung metastatic nodules' number in BALB/c mice with H22‐WDR6 cells orthotopically implantation when compared to IgG control (*n* = 5/group). Data information: All results are representative data generated from three independent experiments. Data are presented as mean ± SD. Unpaired two‐tailed Student's *t*‐test (Ei–G) were used for statistical analysis, and long‐rank (Mantel‐cox) test for survival comparison (C).

To reveal the effects of WDR6 on TIME, we constructed liver‐specific *WDR6* knockout (KO) mice (*Albumin‐Cre* ^/+^; *Wdr6* ^*f/f*^ mice) (Fig 3A and B). Consistent with the above findings, wild‐type (WT)‐*WDR6* mice with N‐nitrosodiethylamine (DEN)‐induced HCC indicated shorter OS relative to *WDR6*‐KO mice (Fig 3C). Compared with the control group, *WDR6*‐KO mice showed an immune profiling with less tumor‐suppressive polymorphonuclear myeloid‐derived suppressor cells (PMN‐MDSC, CD11b^+^Ly6G^+^Ly6C^lo^) recruitment, but higher T‐cell counts (Fig 3D and E). No significant difference was observed for monocytic (M‐MDSC) and tumor‐associated macrophages (TAM). In the orthotopic tumors from BALB/c mice introduced with H22‐WDR6 cells, we observed the opposite change (Fig 3F). Notably, α‐Gr1 antibody injection strongly decreased tumor volume and lung metastatic number in BALB/c mice with H22‐WDR6 overexpression compared with IgG control (Fig 3G). Thus, WDR6 promotes HCC development mainly by reprogramming TIME with minimal CD8^+^ T cells accumulation and abundant MDSCs.

### WDR6 activates TNFα‐mediated migration of MDSCs in HCC tissues

**Figure 4:**
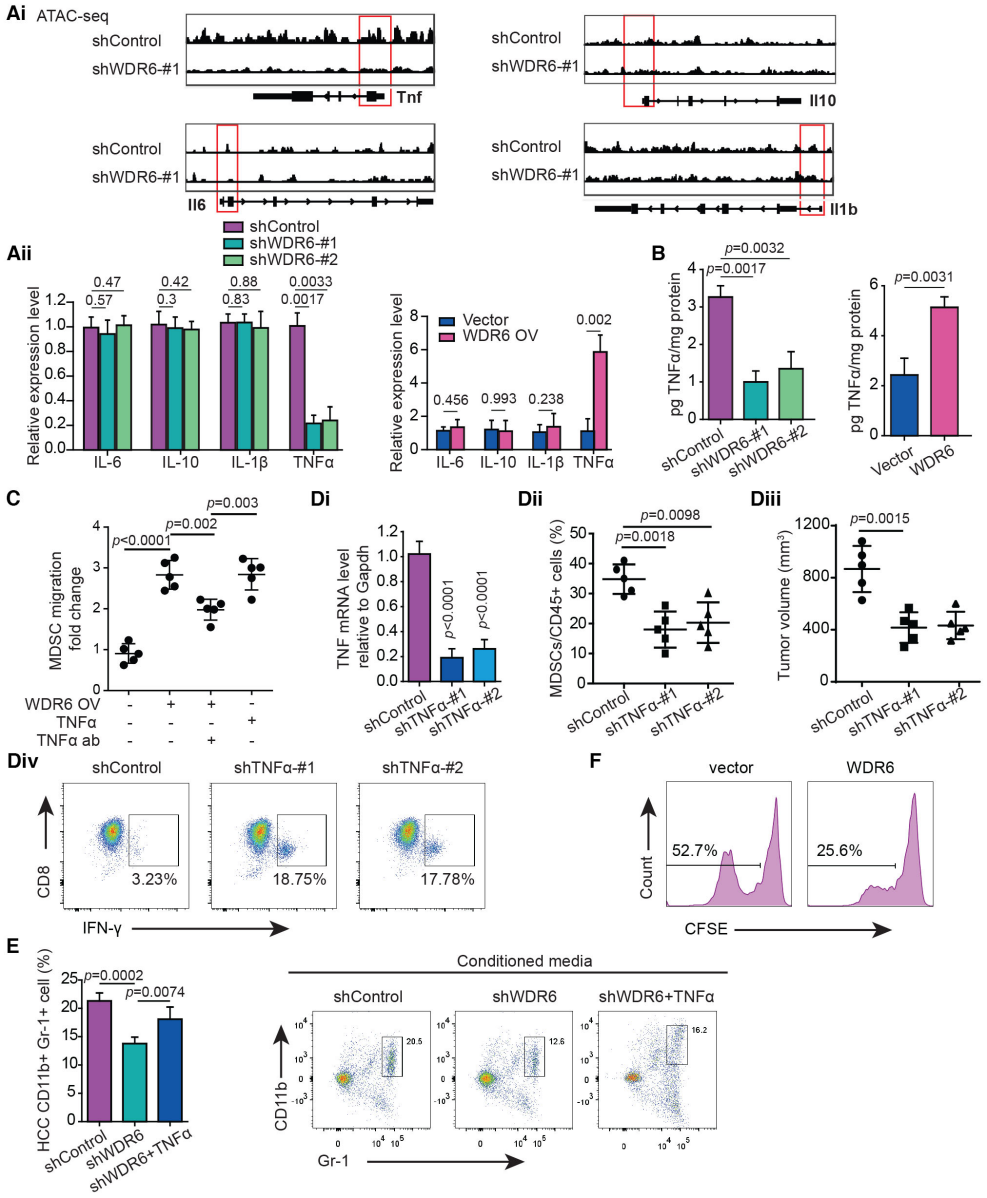
WDR6 activates TNFα‐mediated migration of MDSCs in HCC tissues AiATAC‐sequencing showed the effects of *WDR6* knockdown on the chromatin accessibility of IL10, IL1β, IL6, and TNFα loci.AiiQuantitative real‐time polymerase chain reaction (qRT‐PCR) analyzing cytokines' mRNA levels in Hepa1‐6 and H22 cells treated with or without *shWDR6* or WDR6, respectively.BTNFα ELISA assays were performed in the conditioned medium from Hepa1‐6 and H22 cells with *WDR6* knockdown or overexpression, respectively.CThe effects of the conditioned medium from H22 cells with WDR6 overexpression on the migration of isolated MDSCs in the presence of TNFα or TNFα antibody.Di–DivThe effects of *TNFα* knockdown in H22‐WDR6 cells on MDSC recruitment (Dii), tumor size (Diii), and the ratio of IFN‐γ^+^ CD8^+^ T cells (Div) in tumors from syngeneic mice (*n* = 5/group). *TNFα* knockdown was confirmed by qRT‐PCR (Di).ETNFα supplementation decreased MDSC infiltration induced by *WDR6* knockdown in tumor tissues from syngeneic mice (*n* = 6/group).FAfter sorted from vector and H22‐WDR6 mice' liver, PMN‐MDSCs were cultured at the ratio of 2:1 with CFSE‐labeled splenic CD3^+^ T cells from BALB/c mice. Flow cytometry was performed to examine CFSE^low^ proportion in CD3^+^ T cells (*n* = 3). Data information: All results are representative data generated from three independent experiments. Data are presented as mean ± SD. One‐way ANOVA (Ai–E) were used for statistical analysis.

IL10, IL1β, IL6, and TNFα have been shown to enhance MDSC recruitment in TIME (Beury *et al*, 2014). To clarify whether WDR6 dysregulation was affecting these cytokines, and thus MDSC recruitment, we performed ATAC‐sequencing. This assay indicated that *WDR6* knockdown in Hepa1‐6 cells only decreased the chromatin accessibility at the TNFα locus (Fig 4Ai). Consistently, *WDR6* knockdown downregulated the mRNA levels of TNFα but not IL6, IL1β, and IL10, while WDR6 overexpression had the opposite effect (Fig 4Aii, Appendix Fig S1E). Meanwhile, measuring the supernatant from the same cell lines revealed decreased TNFα secretion after *WDR6* knockdown and increased TNFα level in association with WDR6 overexpression (Fig 4B, Appendix Fig S1F). *In vivo*, TNFα secretion was reduced in DEN‐induced HCC of liver‐specific *WDR6‐KO* mice relative to the control.

Although the conditioned medium from H22 cells with WDR6 overexpression promoted MDSC migration to a similar extent as the supplementation of TNFα, this effect was reverted after a TNFα‐neutralizing antibody treatment (Fig 4C, Appendix Fig S1G). *TNFα* knockdown in WDR6‐overexpressing H22 cells (H22‐WDR6) resulted in less MDSC infiltration and smaller tumors in syngeneic mice compared to the control, in addition to increased intratumoral infiltration of IFN‐γ^+^ CD8^+^ T cells (Fig 4D). Conversely, TNFα supplementation reverted the downregulation of MDSC maintenance mediated by *shWDR6* in HCC tissues (Fig 4E). *In vitro* coculture experiments confirmed that MDSCs from the syngeneic mice with H22‐WDR6 cells blocked T‐cell proliferation (Fig 4F). These data suggest that TNFα takes part in the WDR6‐enhanced recruitment of MDSCs in HCC.

### A WDR6/NF‐κB feedback loop upregulates TNFα expression by directly impairing autophagy‐dependent degradation of p65 in HCC

**Figure 5:**
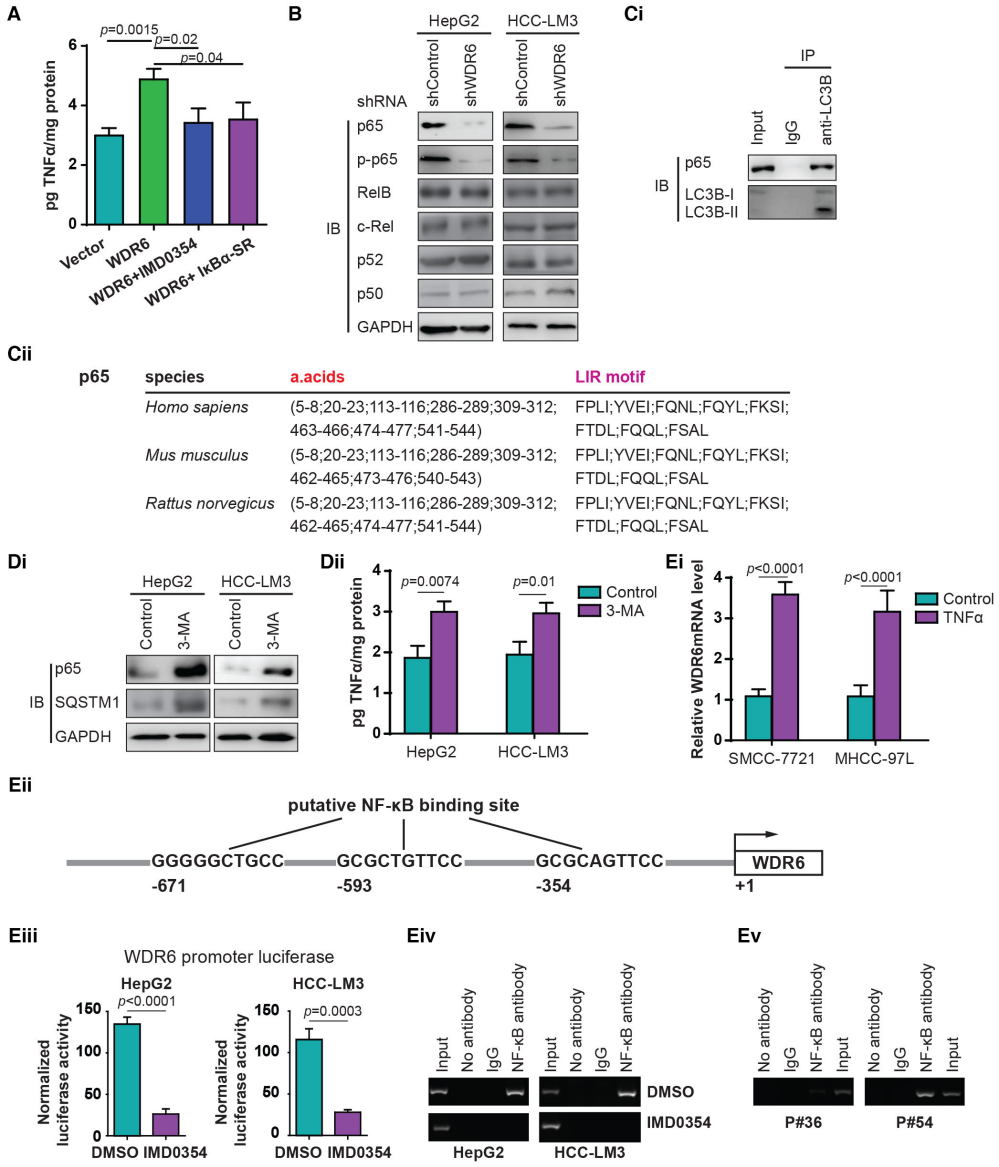
WDR6/NF‐κB feedback loop upregulates TNFα expression by impairing autophagy‐dependent degradation of p65 in HCC AThe effects of NF‐κB inhibitor IMD0354 or IκBα‐SR on TNFα expression in MHCC‐97L cells with or without *WDR6* overexpression.BThe effects of *WDR6* knockdown on the protein levels of p65, RelB, c‐Rel, p52, and p50 in HCC cells.CiCo‐immunoprecipitation identified p65 as an interactor of LC3B in MHCC‐97L cells.CiiSchematic representation of the location of evolutionarily conserved LIR motifs in the sequence of p65 protein.Di, DiiThe effects of autophagic deficiency using 3‐MA on the levels of p65 protein and TNFα secretion in HCC cells with *WDR6* knockdown.Ei
*WDR6* mRNA levels in Hep3B and MHCC‐97L cells treated with or without TNFα in culture media.EiiSchematic diagram showing three candidate NF‐κB‐binding sites in *WDR6* promoter.EiiiThe impact of IMD0354 on the luciferase reporter of *WDR6* proximal promoter in HCC cells.Eiv, EvChIP assays using HCC cells or HCC tissues from patients (P#36 and P#54). Data information: All results are representative data generated from three independent experiments. Data are presented as mean ± SD. Unpaired two‐tailed Student's *t*‐test (A, Dii, Ei, Eiii) was used for statistical analysis. Source data are available online for this figure.

Next, we wanted to elucidate the mechanism by which WDR6 upregulates TNFα secretion in HCC. Given the RNA sequencing data in Fig 2E, we focused on NF‐κB, one well‐known TNFα‐associated transcriptional factor (Hayden & Ghosh, 2014). Indeed, NF‐κB inhibitor IMD0354 or IκBα‐SR reverted TNFα expression enhanced by WDR6 overexpression (Song *et al*, 2014) (Fig 5A), indicating that NF‐κB is a critical effector downstream of WDR6 upregulating TNFα secretion in HCC cells. The chromatin immunoprecipitation (ChIP) assays further confirmed the binding of p65 to TNFα (Appendix Fig S2B). Since *WDR6* knockdown did not change the mRNA levels of the five members (p65, RelB, c‐Rel, p52, and p50) of the NF‐κB family (Appendix Fig S2A), but did downregulate the protein levels of p65 and pp65 (Fig 5B), we reasoned that WDR6 might activate NF‐κB pathway by blocking the degradation of the p65 protein.

Ubiquitination and autophagy regulate protein degradation (Feng *et al*, 2020). Since Fig 2Eiii suggested WDR6 may be involved in the autophagy‐associated pathway (Fig 2Eiii), we hypothesized that WDR6 might upregulate TNFα expression by blocking autophagy‐dependent degradation of p65. To confirm this idea, we performed immunoprecipitation of microtubule‐associated protein 1 light chain 3B (LC3B) in HCC cell lysates. We found that p65 co‐immunoprecipitated with LC3B (Fig 5Ci). Because LC3B‐interacting region (LIR) motifs (W/Y/FXXI/L/V) are essential for the recognition of autophagic cargo by ATG8‐like proteins and LC3B (Putyrski *et al*, 2020), we examined the p65 protein sequence in which eight highly conserved LIR motifs were identified across species (Fig 5Cii). Moreover, autophagic deficiency induced by 3‐methyladenine (3‐MA) treatment significantly suppressed p65 protein level and TNFα expression in HCC cells with *WDR6* knockdown (Fig 5Di and Dii). However, the mRNA levels of p65 and TNFα were not significantly affected, thereby making it unlikely that they are transcriptionally regulated. Thus, we conclude that p65 constitutes one substrate of autophagy in HCC cells with *WDR6* deficiency.

Interestingly, we found that TNFα treatment upregulated WDR6 expression (Fig 5Ei). We noted three NF‐κB binding motifs in the promoter of *WDR6*, suggesting a possible mechanism by which TNFα may promote WDR6 expression in HCC (Fig 5Eii). We then demonstrated that NF‐κB was indeed a novel activator for *WDR6* transcription in HCC cells by using the NF‐κB inhibitor IMD0354 and ChIP assays (Fig 5Eiii and Eiv). The ChIP assay confirmed the binding of p65 to WDR6 promoter in the patient sample P#54 that expressed high WDR6 but not in the patient sample P#36 that had low WDR6 expression (Fig 5Ev). Therefore, TNFα recruits NF‐κB to bind the *WDR6* promoter in HCC, establishing a WDR6‐NF‐κB‐TNFα feedback loop.

Together, the WDR6/NF‐κB loop upregulates TNFα expression by directly impairing autophagy‐dependent degradation of p65 in HCC.

### WDR6 targets the autophagic initiator UVRAG to the CUL4A/DDB1/ROC E3 ligase complex through its WDxR motif and promotes UVRAG ubiquitination

**Figure 6:**
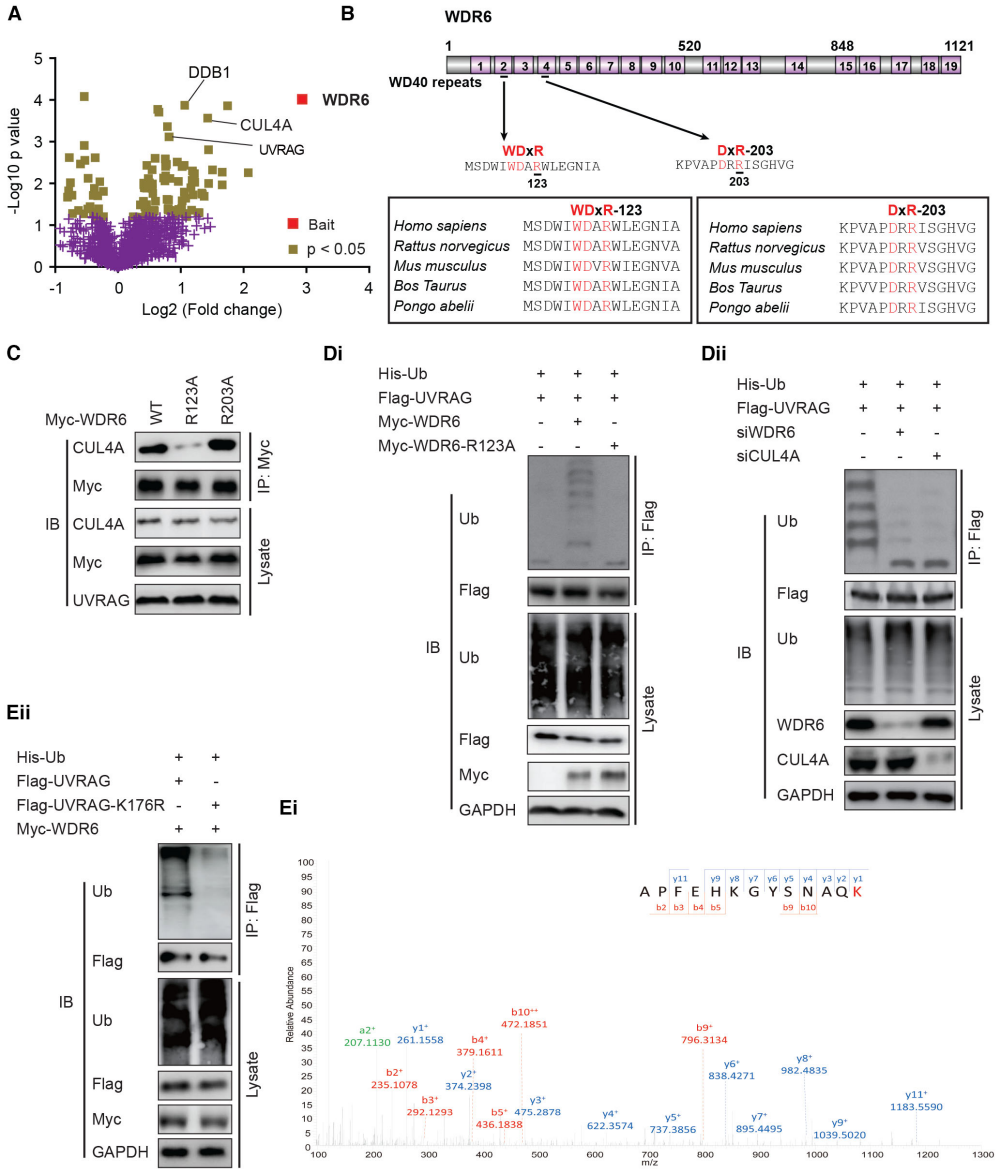
WDR6 targets UVRAG to CUL4A/DDB1/ROC E3 ligase complex through its WDxR motif AVolcano plot indicating the interactors of WDR6 identified in SMCC‐7721 cells with Empty‐BioID2 or WDR6‐BioID2 (*n* = 5).BThe conserved WDxR and DxR motifs in the WD40 repeats of WDR6 protein.CCo‐IP analysis indicated the effect of WDR6 mutant (R123A) or (R203A) on the binding of CUL4A to endogenous UVRAG in MHCC‐97L cells.DiUVRAG ubiquitination assay in MHCC‐97L cells expressing *WT‐WDR6* or its mutant *WDR6*‐R123A.DiiUVRAG ubiquitination in HCC‐LM3 cells treated as shown.EiMS assay identified K176 of UVRAG as major ubiquitination site.Eii
*K176R mutant* abolished UVRAG ubiquitination promoted by WDR6. Data information: All results are representative data generated from three independent experiments. Source data are available online for this figure.

To clarify the mechanism by which WDR6 blocked autophagy‐dependent degradation of p65, we performed a proximity‐dependent biotin experiment (Zhou *et al*, 2020). This screen identified several proteins such as damaged DNA‐binding protein 1 (DDB1), CUL4A E3 ligase, and ultraviolet irradiation resistance‐associated gene (UVRAG) in the WDR6 immunocomplex (Fig 6A). Since UVRAG is a critical autophagy initiator, and the member of the WDR family has been reported to enhance CUL4/DDB1‐mediated ubiquitination (Choe *et al*, 2009), we postulated that WDR6 might disrupt autophagy and activate the p65‐TNFα pathway by enhancing CUL4A‐mediated ubiquitination and degradation of UVRAG.

To test this idea, we explored whether WDR6 recruited UVRAG to the CUL4A/DDB1 E3 complex by serving as a substrate adaptor. As shown in Appendix Fig S2Ci and Cii, WDR6 was capable of binding endogenous and exogenous UVRAG. Importantly, *WDR6* depletion compromised the binding of UVRAG to endogenous DDB1 and CUL4A but not CUL4B (Appendix Fig S2Ciii), demonstrating that WDR6 acts as a substrate adaptor. Previous studies reported that the proteins containing WD40 repeats interacted with other proteins via a WDxR or DxR motif (Choe *et al*, 2009). Given that WDR6 is a WD40‐repeat protein possessing two conserved WDxR and DxR motifs (Fig 6B), we reasoned that WDR6 might recruit UVRAG to CUL4A‐DDB1 E3 ligase via these two motifs. To test this hypothesis, we generated two mutants WDR6‐R123A and WDR6‐R203A with arginine (R) at amino acid 123 or 203 changed to alanine (A), respectively (Fig 6B). We observed that the WDR6‐R123A but not R203A mutant significantly decreased the binding of CUL4A to UVRAG (Fig 6C). Therefore, the WDxR motif determined the formation of the WDR6/UVRAG/CUL4A/DDB1 complex.

Next, we examined whether this E3 ligase complex mediated UVRAG ubiquitination. *In vivo* ubiquitination assays indicated that WDR6 but not WDR6‐R123A overexpression enhanced the ubiquitination of UVRAG (Fig 6Di). However, WDR6 or CUL4A knockdown has the opposite effect (Fig 6Dii). WDR6 recruited CUL4A but not CUL4B to ubiquitinate UVRAG (Appendix Fig S2D). Using mass spectrometry (MS) assay, we identified K176 of UVRAG as a primary ubiquitination site (Fig 6Ei) and with its mutation to K176R, ubiquitination was abolished (Fig 6Eii). Therefore, WDR6 promotes UVRAG ubiquitination at K176 by functioning as a substrate adaptor for the CUL4A E3 ligase.

### WDR6/CUL4A‐mediated ubiquitination and degradation of UVRAG promotes TNFα expression, MDSC recruitment, and HCC progression

**Figure 7:**
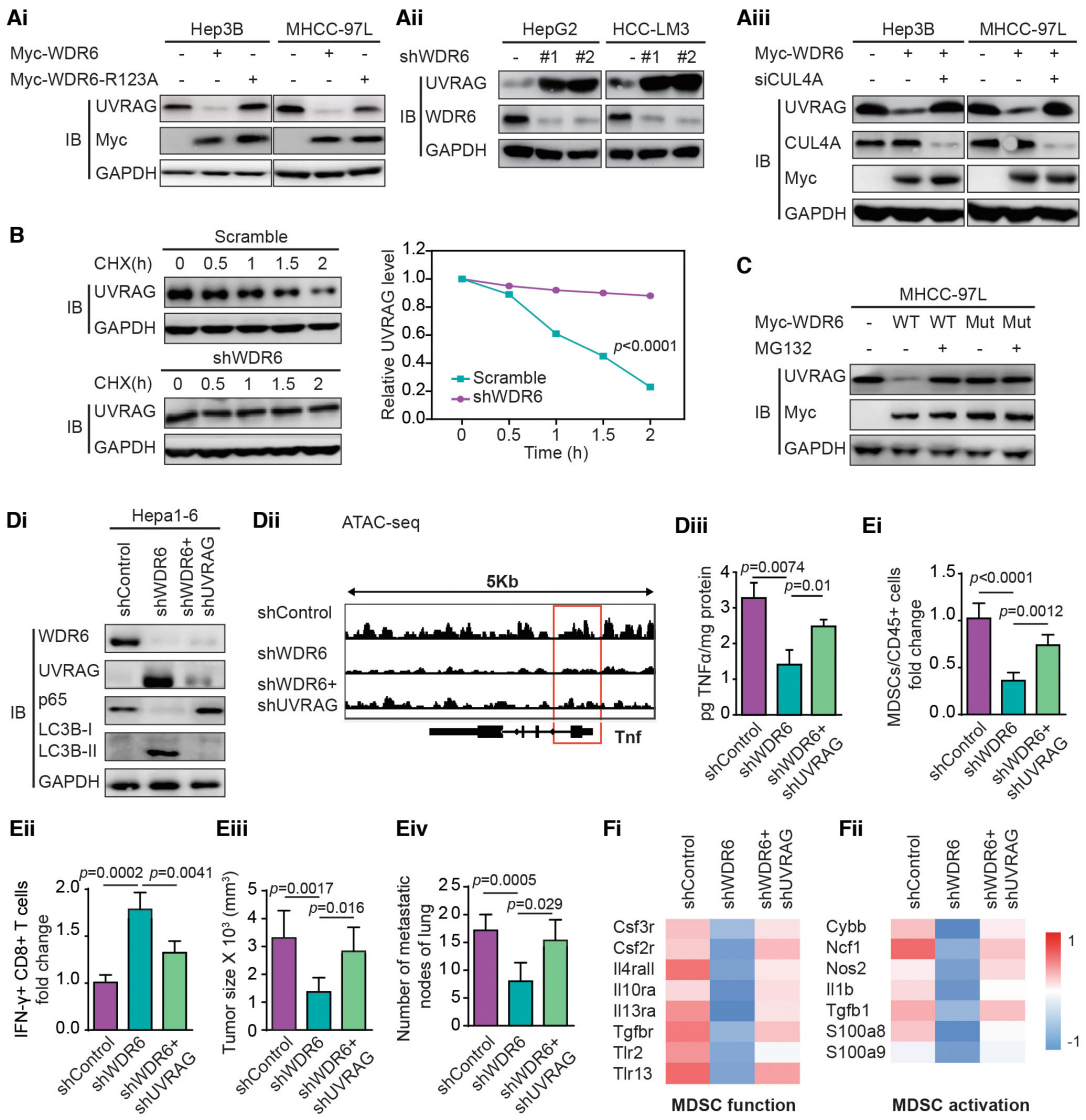
WDR6/CUL4A‐mediated ubiquitination and degradation of UVRAG promotes TNFα expression, MDSC recruitment, and HCC progression Ai–Aiii
*WDR6* or its mutant (Ai) or *WDR6* shRNAs (Aii) or WDR6 with *CUL4A* siRNA (Aiii) affected the levels of endogenous UVRAG in HCC cell lines.BCHX assays of UVRAG stability in HCC‐LM3 cells transfected with *WDR6* shRNA.CThe effects of WT‐WDR6 or *WDR6‐R123A* mutant on UVRAG levels in MHCC‐97L cells in the presence of MG132.Di–Diii
*UVRAG* knockdown in Hepa1‐6 cells reverted *WDR6*‐deficient‐induced chromatin accessibility of TNFα locus and TNFα secretion in culture medium (Diii). The cells in (Di) were orthotopically implanted into C57BL/6J mice. And the partial data in (Dii) were from Fig 4Ai.Ei–EivMDSC recruitment (Ei), IFN‐γ^+^ CD8^+^ T cells (Eii), tumor size (Eiii), and lung metastasis (Eiv) were recorded (*n* = 6/group).Fi–FiiHeat‐map showing the expression of altered genes related to functional signatures (Fi: MDSC function, Fii: MDSC activation) in MDSCs after *WDR6* alone or with UVRAG knockdown. Data information: All results are representative data generated from three independent experiments. Data are presented as mean ± SD. One‐way ANOVA (B) and unpaired two‐tailed Student's *t*‐test (Diii, Ei–Eiv) were used for statistical analysis. Source data are available online for this figure.

Next, we investigated the consequence of UVRAG ubiquitination by the WDR6‐CUL4A complex. As expected, overexpression of WT‐WDR6 compared to the *WDR6‐R123A* mutant deceased UVRAG abundance in Hep3B and MHCC‐97L cells (Fig 7Ai). Conversely, *WDR6* knockdown in HepG2 and HCC‐LM3 cells increased UVRAG protein levels (Fig 7Aii). However, *CUL4A* knockdown rescued the effect of WDR6 overexpression on UVRAG protein (Fig 7Aiii). Cycloheximide (CHX) assay revealed that knockdown of *WDR6* stabilized the levels of endogenous UVRAG (Fig 7B). Furthermore, MG132 treatment stabilized the levels of UVRAG protein in cells with WT‐WDR6 compared to cells with the *WDR6 R123A* mutant (Fig 7C). Therefore, WDR6/CUL4A‐mediated ubiquitination elicits UVRAG proteasomal degradation.

Importantly, introducing *shWDR6* into Hepa1‐6 cells significantly decreased p65 expression, chromatin accessibility at the TNFα locus, and TNFα secretion, while the levels of UVRAG and autophagy were enhanced (Fig 7Di–Diii). After these cells were orthotopically implanted into C57BL/6J mice, *WDR6* knockdown reduced MDSC recruitment and HCC progression, but increased IFN‐γ^+^ CD8^+^ T cells in the TIME (Fig 7Ei–Eiv). However, after a *shUVRAG* lentiviral vector was co‐introduced to inhibit UVRAG expression, the effects of *WDR6* knockdown were significantly suppressed (Fig 7D and E). Furthermore, loss of *WDR6* with or without *shUVRAG* co‐introduction extensively changed the gene expression profile of MDSCs, which was categorized into two subgroups. Although loss of *WDR6* inhibited genes involved in activation and function of MDSCs, these effects were reversed by *shUVRAG* co‐introduction (Fig 7Fi and Fii). These data indicate that UVRAG serves as a key effector downstream of WDR6 in HCC.

### A WDxR‐like peptide increases the efficacy of anti‐PD‐L1 therapy in HCC treatment

**Figure 8:**
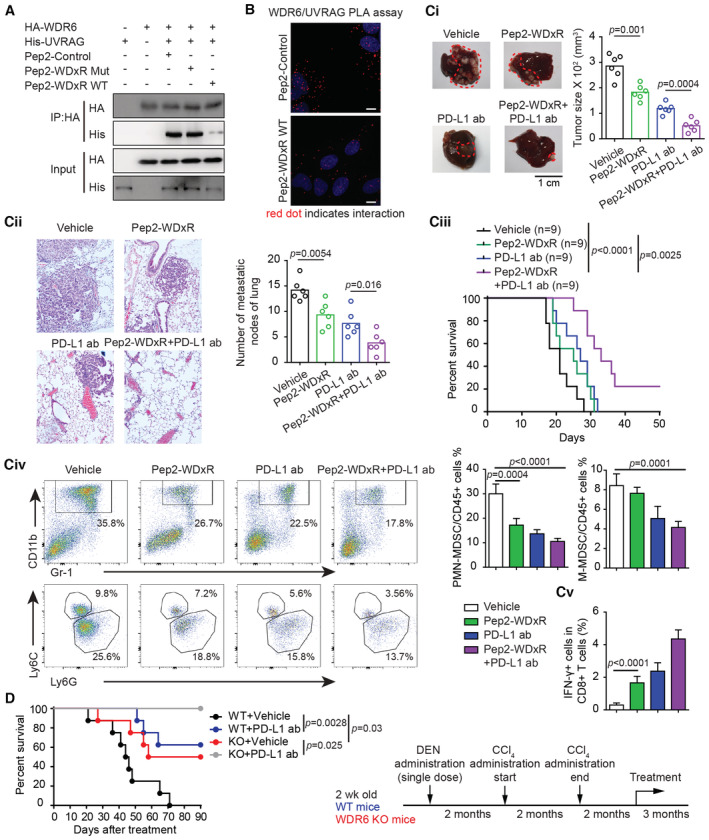
A WDxR‐like peptide increases the efficacy of anti‐PD‐L1 antibody against HCC AHEK293T cells with indicated constructs were treated with 5 μM Pep2‐WDxR or Pep2‐con or Pep2‐WDxA mutant for 16 h, respectively. Then co‐IP assay was used to assess the impacts of these peptides on the WDR6‐UVRAG binding.BPLA assay examining the co‐localization of WDR6 and UVRAG in HEK293T cells described in (A). Scale bars: 10 μm.Ci–CvBALB/c mice introduced with H22‐WDR6 overexpression cells were administered with Pep2‐WDxR or Pep2‐con with or without anti‐PD‐L1 antibody. The images and sizes of HCC (Ci), hematoxylin–eosin (H&E) staining and metastasis nodules of lung were presented (Cii). The survival analysis of the mouse after treatment was performed (*n* = 9/group) (Ciii). In the BALB/c mice from (Ci), flow cytometry was performed to analyze PMN‐MDSC, M‐MDSC, and IFN‐γ^+^ CD8^+^ T cells in tumors (Civ).DThe survival test of DEN‐ and CCl_4_‐induced HCC using *WDR6*‐knockout and *WT* mice. At 26 weeks, mice were administered with or without anti‐PD‐L1 antibody (*n* = 6/group). Data information: All results are representative data generated from three independent experiments. Data are presented as mean ± SD. One‐way ANOVA (Ci, Cii, Civ, Cv), were used for statistical analysis, and long‐rank (Mantel–Cox) test for survival comparison (Ciii, D). Source data are available online for this figure.

Since the WDR6/UVRAG/TNFα axis caused the accumulation of MDSCs and decreased the infiltration activation of T cells in HCC tissues, the efficiency of immune checkpoint therapy such as anti‐PD‐1 antibody should be significantly inhibited. Given that the WDxR motif determined the binding of WDR6 to UVRAG, blocking this binding with one WDxR‐like peptide might improve the effectiveness of anti‐PD‐L1 antibody by reprogramming the TIME in HCC. To test this hypothesis, we designed a peptide Pep2‐WDxR by fusing the WDxR to Pep2 as described previously (Li *et al*, 2014). Proximity ligation assay (PLA) indicated that Pep2‐WDxR‐WT disrupted the WDR6/UVRAG complex and co‐localization in HEK293T cells (Fig 8A and B). However, the Pep2‐mut peptide carrying R to A substitution lost its inhibitory capability (Fig 8A). We then used a syngeneic HCC model with H22‐*WDR6* overexpression to study the effect of Pep2‐WDxR peptide on the immune checkpoint inhibitor. As shown in Fig 8Ci and Cii, although a single treatment with Pep2‐WDxR peptide or anti‐PD‐L1 antibody markedly reduced tumor growth and lung metastasis, the combined treatments were more effective. A prolonged survival was observed in the combination group but not in the single treatment group (Fig 8Ciii). Consistently, either Pep2‐WDxR peptide or anti‐PD‐L1 antibody markedly decreased HCC‐associated MDSC abundance, but increased the abundance of IFN‐γ^+^ CD8^+^ T cells (Fig 8Civ).

We also studied the effects of targeting WDR6 with anti‐PD‐L1 antibody on the carbon tetrachloride (CCl_4_) and DEN‐induced HCC using *WDR6*‐knockout and *WT* mice. The results indicated that combined therapy was much better for the survival of the tumor‐bearing mouse than single treatment (Fig 8D).

Collectively, the Pep2‐WDxR peptide is helpful for enhancing the efficacy of anti‐PD‐L1 in treating HCC with WDR6 dysregulation.

### The combination of WDR6, pp65, and UVRAG levels indicates an important prognostic value for HCC

To reveal the clinical significance of the WDR6/UVRAG/NF‐κB axis, we performed IHC analysis, revealing an inverse correlation between WDR6 and UVRAG in HCC patients (*n* = 110) (Appendix Fig S3A and Bi, Appendix Tables S4 and S5). Additionally, upregulation of pp65 was associated with WDR6 increase (Appendix Fig S3Bii and C). The Kaplan–Meier analysis suggested that high WDR6 or high pp65 with low UVRAG expression in HCC predicted an adverse outcome (Appendix Fig S3Di–Diii). This indicated that combining these three parameters may improve the prognostic accuracy. Therefore, targeting this axis may be a novel strategy for treating HCC with WDR6 dysregulation (Appendix Fig S3E).

## Discussion

In this study, the primary findings are: (i) Functional loss or gain of *WDR6* does not significantly change the malignant state of HCC cells *in vitro* or in nude mice, while *WDR6* deficiency in Hepa1‐6 cells inhibits the orthotopically implanted tumors in immune‐competent C57BL/6J mice; (ii) The WDR6/UVRAG axis upregulates chromatin accessibility of TNFα locus and TNFα secretion by blocking the autophagic degradation of p65, which elevates intratumoral MDSC and reduces CD8^+^ T cells; (iii) TNFα upregulates the promoter activity of *WDR6* via NF‐κB, establishing a feedback loop, (iv) Clinically, WDR6‐mediated UVRAG degradation pathway is hyperactivated in HCC and closely correlated to poor prognosis; and (v) A WDxR motif‐like peptide disrupts the WDR6/UVRAG binding and inhibits HCC progression by reprogramming the TIME. Together, our experimental, mechanistic, and clinical evidence highlights a vital role of a WDR6/UVRAG/ NF‐κB loop in HCC development.

Although dysregulation of WDR6 has been reported in several human malignancies (Yafune *et al*, 2013; Xu *et al*, 2017; Savci‐Heijink *et al*, 2019), the direct signaling network linking WDR6 to a cancer‐propagating program remains unknown. In this work, we define an unrecognized molecular pathway by which WDR6 elevates TNFα production and intratumoral MDSC recruitment but reduces CD8^+^ T cells infiltration, thereby promoting an immunosuppressive and prometastatic microenvironment in HCC. This novel mechanism is consistent with the following facts: (i) the TIME has significant effects on tumor growth and metastasis, and expanding MDSCs often is an efficient strategy adapted by HCC cells to defeat immune surveillance (Houthuijzen & Jonkers, 2018); (ii) HCC is a typical inflammation‐associated tumor characterized by aberrant immune cell infiltration and cytokine or chemokine secretion (Keenan *et al*, 2019). Unsurprisingly, this novel finding confirms that targeting the WDR6/UVRAG/NF‐κB/TNFα loop not only inhibits HCC progression but also produces a synergistic effect with anti‐PD‐L1 therapy. Thus, future efforts aimed at targeting these markers may lead to the development of new treatments against HCC. Notably, while preparing this manuscript, another group reported the WDR6/WDR3/DDB1/CUL4 E3 ligase complex‐mediated ubiquitination of OSR1 kinase (Dhiani & Mehellou, 2020), further supporting our findings.

Another exciting finding is about UVRAG degradation. Almost all autophagy‐related proteins are regulated by posttranslational modifications (PTMs) (Feng *et al*, 2020). It has been reported that UVRAG can be ubiquitinated by SMURF1 at K517 and K559 (Feng *et al*, 2019). However, this K29 and K33‐linked UVRAG poly‐ubiquitination does not cause its degradation (Feng *et al*, 2019). Disrupting the UVRAG‐SMURF1 complex via PPxY motif inhibits UVRAG ubiquitination‐mediated autophagosome maturation and HCC development (Feng *et al*, 2019). The current study extends this previous finding about UVRAG by elucidating the WDR6‐mediated CUL4A E3 ligase recruitment to UVRAG, which leads to UVRAG ubiquitination‐dependent degradation and thereby changing the MDSC infiltration in HCC tissues. These data uncover a novel PTM of UVRAG, a novel ubiquitination site at K176, and a novel functional significance in reprogramming the TIME. Surprisingly, one study has reported that UVRAG is an activator of the DDB1‐CUL4A‐Roc1 ubiquitin ligase complex by promoting the neddylation and assembly of this E3 ligase complex in DNA damage repair (Yang *et al*, 2016), indicating an interplay between UVRAG and the CUL4A E3 ligase complex in the tumor‐associated background. Notably, our previous findings revealed that several E3 ligases (HECTD1, UBA1, and FBXL15) also were in the UVRAG co‐immunoprecipitation complexes (Feng *et al*, 2019). However, it is unclear whether these E3 ligases can induce the ubiquitination and degradation of UVRAG in a background‐dependent way. Further study is warranted for this unsolved question.

We also are interested in two previous observations: (i) A genome‐wide knockdown screening suggesting a possible role of WDR6 in starvation‐induced autophagy (McKnight *et al*, 2012); (ii) WDR6 co‐immunoprecipitation with LKB1, an important kinase upstream of AMPK (Xie *et al*, 2007). Although these reports do not reveal the role and action mechanisms of WDR6 in autophagy, these data indeed suggest that WDR6 is involved in autophagy regulation. These clues are consistent with our RNA‐sequencing data and the regulation of UVRAG by WDR6, supporting a hypothesis that WDR6 may modulate other autophagy‐related proteins besides UVRAG, LKB1, and AMPK. Given that WDR6 is a coactivator of CUL4A E3 ligase (Yang *et al*, 2016), we postulate that WDR6 may regulate LKB1 ubiquitination. Of course, further study is needed to clarify this hypothesis. Elucidating these questions will greatly contribute to a better understanding of autophagic regulation and improve tumor therapy.

Interference or inhibitory peptides (iPeps) have recently appeared as a novel tool for drug development (Sorolla *et al*, 2020). These iPeps are usually comprised of several critical residues of a protein which are necessary for protein–protein binding. By serving as dominant‐negative inhibitors, these iPeps can precisely hit their intracellular targets, thereby combining the advantages of chemical therapies and biologics (Lee *et al*, 2019). Precise targeting of the WDR6/UVRAG‐related pathological activity offers the advantage of minimizing potential undesirable side effects, which is of paramount importance for tumor therapy. Specific targeting of WDR6/UVRAG axis also appears more favorable than targeting UVRAG alone because UVRAG is ubiquitously expressed and required for critical physiological roles. In contrast, the frequent selective overexpression of WDR6 in tumors makes it an extremely attractive target for the development of small‐molecule inhibitors in anticancer therapies. However, so far, cell‐penetrating peptide treatment has major drawbacks for clinical use such as specificity and stability issues. Therefore, it is too early to expect that WDR6 can be a valid target in human HCC.

In summary, we define that WDR6 serves as a novel risk factor in HCC progression. Importantly, this study uncovers a close link between the WDR6/UVRAG axis and HCC immunosuppression, thereby providing the foundation for the development of small‐molecule drugs that specifically target the TNFα‐related promalignant functions of WDR6.

## Materials and Methods

### Mice

Liver‐specific *WDR6 knockout* (*KO*) mice were generated on the C57BL/6 background. A schematic genomic map of wild‐type, floxed, and deleted alleles were shown in Fig 3. Loxp sites (red triangles) were inserted in introns 2 and 3. Liver‐specific cre recombination (Albumin‐Cre) results in exon 2 deletion and *Wdr6* gene silence. Genotyping primers included: *Wdr6*: (F) 5′‐CTTTGGTGATGGTGGTGTTGG; (R) 5′‐GCTGAGGCTAATAATGGCTGAC; *Cre*: (F) 5′‐GTGTTGCCGCGCCATCTGC; (R) 5′‐CACCATTGCCCCTGTTTCACTATC. Animals were housed under specific pathogen‐free (SPF) conditions in a 12 h/12 h light/dark cycle with access to standard chow and autoclaved tap water. The use of mice in this project was agreed upon the Committee on the Use of Animals in Research and Teaching, the affiliated Hospital of Peking and Hebei Medical University. No statistical methods were used to predetermine sample size. At least five animals were used per group in all animal experiments. Animals were allocated into groups randomly, and the surgeries, harvest, and analysis of animals were blind to reduce subjective bias. No inclusion or exclusion criteria were conducted on the animals.

### Orthotopic implantation mice HCC models and flow cytometry assays

Firstly, mouse WDR6‐knockdown hepa1‐6 cells or ‐overexpression H22 cells or human HCC cells were generated. The selection was performed using puromycin. For orthotopic implantation, the luciferase‐labeled HCC cells with WDR6‐knockdown or ‐overexpression (1 × 10^6^) were implanted into the left‐lobe liver in the nude mouse (BALB/c nude, 5–7‐week‐old, female, purchased from the Shanghai Laboratory Animal Center at the Chinese Academy of Sciences), while hepa1‐6 cells with mouse WDR6‐knockdown (3 × 10^6^) in C57BL/6J mice (5–7‐week‐old, female, purchased from the Shanghai Laboratory Animal Center at the Chinese Academy of Sciences), and H22 cells with mouse WDR6‐overexpression (2 × 10^6^) in BALB/c mice (5–7‐week‐old, female, purchased from the Shanghai Laboratory Animal Center at the Chinese Academy of Sciences). For luciferase‐labeled mice, the Xenogen IVIS 100 Imaging System was used to get the bioluminescent images after D‐luciferin was intraperitoneally administered. For drug experiments, 3 days after the orthotopically implanted operation with H22 cells, BALB/c mouse was injected via i.p. using vehicle or Pep‐WDxR, with or without PD‐L1 antibody (10F.9G2, Bio X Cell, 2 mg/kg) or IgG antibody (4 mg/kg), respectively. For DEN and CCl4‐induced HCC model, i.p. injection of DEN (1 mg/kg) was performed for C57BL/6 *WT* or *WDR6‐KO* mice (2 weeks old). Eight weeks later, CCl4 (1.25 ml/ kg, 30% v/v in olive oil, Sigma‐Aldrich) was administrated twice weekly for another 8 weeks. In the 26th week, i.p. injection of anti‐PD‐L1 antibody (2 mg/kg) was performed twice weekly. Tissues were collected at the indicated time. The sizes of tumors were measured, and calculated as 0.5 × length × width × width, metastasis nodules were numbered, and histologic analysis was performed. Anesthesia was administrated to make mice suffer minimum under surgery. The gentleMACS dissociator from Miltenyi Biotech was utilized to dissociate tumor samples from mice following the producer's protocols. Then 70 μm cell strainers were used to filter the cell suspensions. The lysing buffer from Qiagen was used to remove the red blood cells in the samples. After anti‐mouse Fc Block and fluorophore‐conjugated antibodies staining, these samples were gated for CD45 staining for total leucocytes. To define specific populations, the following markers were used: T cells (CD3^+^, CD4^+^, and CD8^+^), MDSC (CD11b^+^Gr1^+^), NK cells (NK1.1), B cells (B220^+^), and dendritic cells (DCs) (CD11c^+^). Of course, an unstained sample was also included in the gating strategy of pooled cells as a negative control. BD LSRII Analyzer from BD Biosciences was used to examine the samples after processed samples were washed using staining buffer. In addition, UltraComp compensation beads were purchased from eBiosciences and compensation was performed for all of the fluorophores. Back‐gating was also carried out for positive control samples to validate the gating. The FlowJo was utilized to analyze the acquired data.

### MDSC isolation, T‐cell suppression, and deletion assays

Myeloid‐derived suppressor cell isolation kit from Miltenyi Biotech was used to isolate total MDSCs (CD11b^+^Gr1^+^). Briefly, after FcR blocking, a biotin‐conjugated Ly6G or Gr1 antibody was added to the samples. Then antibiotin microbeads were used to label the cells. Magnetic cell separation was performed by loading the samples into the MS separation column. The flow cytometry for the cells retained in the column was performed to examine the purity of MDSCs by identifying the population containing CD11b^+^Ly6C^+^/CD11b^+^Gr^+^ markers. Following the producer's instruction, a T‐cell isolation kit from Miltenyi Biotech was used to purify T cells. Isolated T cells were labeled for 10 min using 2 μM carboxyfluorescein diacetate succinimidyl ester (CFSE, Thermofisher) after washing and counting. The labeled T cells were subsequently co‐cultured in a 96‐well plate with different amounts of CD11b^+^Gr1^+^ MDSCs freshly isolated for 3 days as indicated. T‐cell proliferation was analyzed by flow cytometry based on CFSE‐fluorescence intensity. For the MDSC depletion experiment, IgG (BE0090) or α‐Gr1 (BE0075) was intraperitoneally every other day injected into the mouse (200 μg per mouse). For depleting the CD8^+^ T‐cell population in mice, the mouse was injected with α‐CD8 antibody (BE0117) or isotype‐matched IgG (BE0090) intraperitoneally (200 μg per mouse).

### CyTOF

The single cell suspension was processed as above. Then using reagents and protocols provided by Fluidigm, cells were labeled with a cocktail of metal‐conjugated surface marker antibodies, cisplatin, and iridium. Antibodies were labeled using MaxPar Metal Labelling Kits (DVS). With the Nolan lab bead normalizer package and beads, normalized the samples between runs. Then downloaded Release 3.4 of the cytofkit package from Bioconductor, and PhenoGraph analysis was performed after gated singlet live CD45^+^ events imported into cytofkit. Thus, on tSNE plots, 19 subpopulations of cells or clusters were displayed within the R package “Shiny”.

### Western blot and co‐immunoprecipitation

For performing Western blots, the cells or tissues were dissolved within RIPA buffer. After the samples were separated via SDS–PAGE gels, PVDF membranes were used to transfer the proteins. Then these PVDF membranes were blocked in blocking buffer (TBS + 5% milk), followed by primary antibody and HRP‐conjugated secondary antibody incubation, respectively. The results were observed with enhanced chemiluminescence (ECL) from Bio‐Rad.

For co‐IP, the lysate (800 μg) was first precleared and then incubated with the antibodies indicated in the cold room overnight. Protein A/G Plus‐Agarose was subsequently mixed with the lysates for 1 h to immunoprecipitate the targeted proteins at 4°C. After spinning and washing, 4× SDS loading buffer was used to resolve the pellets. Finally, gel electrophoresis and Western blot were performed as previously described (Ma *et al*, 2019).

### Cycloheximide (CHX) chase assay

Twenty‐four hours after transfection, indicated cells were incubated with 100 μg/ml CHX in DMEM containing 10% FBS. Then the cells were collected by trypsin treatment at different time points post CHX treatment, and lysates were prepared for immunoblotting assay.

### HCC patient tissues

The institutional review board of the University approved the use of patients' samples. The consent forms were signed to make the patients admit their specimens used in this project. All tissues were collected at the affiliated Hospital of the University from September 2016 to August 2018 through surgical resection. All procedures that involved human samples were approved by the Ethics Committee of Nanjing Drum Tower Hospital and adhered to the World Medical Association (WMA) Declaration of Helsinki and to the Department of Health and Human Services Belmont Report.

### Antibodies and reagents

The antibodies for flow cytometry analysis are purchased from Biolegend and listed as follows: mouse CD3 (100235), CD45 (clone 30‐F11), CD8a (100729), NK1.1 (108733), Gr1 (clone RB6‐8C5), CD4 (100422), CD11b (M1/70), Ly‐6G (127617), F4/80 (MB8), CD11c (N418), Ly6C (128015), and B220 (103247). Dilution factor is 1:200 for each antibody.

Other antibodies are listed as follows: anti‐UVRAG (Millipore, AB2960, 1:2,000), anti‐β‐actin (Sigma, A5316‐100ul, 1:3,000), anti‐pp65 (Cell Signaling Technologies, #13346, 1:1,000), anti‐TNF‐α (R&D Systems, MAB4101, 1:2,000), anti‐DDB1 (Abcam, ab109027, 1:2,000), IgG (R&D Systems, MAB005, 1:2,000), anti‐GAPDH (Santa Cruz Biotechnology, sc‐25778, 1:3,000), anti‐IL6 (Santa Cruz Technologies, sc‐28343, 1:500), anti‐CUL4B (Abcam, ab227724, 1:2,000), anti‐TUBA (Sigma, T6199, 1:4,000), anti‐WDR6 (Santa Cruz Technologies, sc‐100897, 1:1,000), anti‐SQSTM1 (Abcam, ab91526, 1:2,000), Goat anti‐Rabbit HRP‐conjugated secondary antibody (Bio‐Rad, 1706515, 1:5,000), anti‐WDR6 (LSBio, LS‐C441947, 1:2,000), anti‐mouse HRP‐conjugated secondary antibodies (Bio‐Rad, 1706516, 1:5,000), anti‐p52 (Cell Signaling Technologies, #4882, 1:2,000), anti‐Flag (Sigma‐Aldrich, F3165), anti‐p50 (Cell Signaling Technologies, #13681, 1:2,000), anti‐HA (Sigma‐Aldrich, 9658‐100, 1:2,000), anti‐c‐Rel (Cell Signaling Technologies, #67489, 1:2,000), anti‐LC3B antibody (Sigma‐Aldrich, L7543, 1:2,000), anti‐IL10 (Santa Cruz Technologies, sc‐8438, 1:1,000), anti‐His (Bethyl Laboratories, A190‐112A, 1:3,000), ani‐RelB (Cell Signaling Technologies, #10544, 1:1,000), anti‐p65 (Cell Signaling Technologies, #6956, 1:1,000), anti‐glutathione S‐transferase (GST) (Santa Cruz Biotechnology, B‐14, 1:2,000), anti‐CUL4A (Santa Cruz Technologies, sc‐377188, 1:500).

Reagents used were as follows: 3‐methyladenine (3‐MA) (Sigma‐Aldrich, M9281), Oligofectamine (Life Technologies, 12252‐011), FuGENE^®^ 6 Transfection Reagent (Promega, E2691), Cycloheximide (Sigma‐Aldrich, C7698), MG132 (Calbiochem, CAS 133407‐82‐6), Prestained Protein Ladder (Thermo Pierce, 26616), puromycin dihydrochloride (ThermoFisher, A1113803), Cell Counting Kit‐8 (CK04) from Dojindo, Protease inhibitor cocktail (Roche, 11697498001), Pierce BCA assay kit (Thermo Fisher Scientific, 23223).

### RNA sequencing, ATAC‐sequencing, gene ontology, and KEGG assays

The Illumina standard kit was utilized for the preparation of mRNA‐sequencing samples based on the manufacturer's instruction. The samples and RNAs were prepared using RNAprotect Cell Reagent (Qiagen) and an RNeasy Plus Micro Kit from Qiagen with in triplicate every group. The Bioanalyzer (Agilent Technologies) was used to evaluate the integrity and quantity of RNAs. The reverse transcription and cDNA amplification proceeded through the Kit from Thermofisher. Library preparation with the Nextera XT Kit from Illumina needs 200 pg cDNA. The Qubit and Tapestation were employed to assess library molarity and quality through a DNA High sensitivity chip that was from Agilent Technologies. Only genes that presented a significant fold change (more than 1.2) were thought about in the RNA‐sequencing assay. Using homemade scripts for the R software, the enrichment of gene ontology (GO) term was carried out. Gene sets were chosen based on various functional pathways and analyzed with version 2.2 of the GSEA package. The score for each gene set was calculated. Transposed DNA purified from HCC‐LM3 cells with *WDR6* knockdown was prepared with The SureCell ATAC‐Seq Library Prep Kit (Bio‐Rad) according to the producer's instructions. The samples were subjected to ATAC sequencing (ATAC‐seq) on a HiSeq X Ten platform (Illumina). The dual indexing and sequencing read length were analyzed following the producer's procedure (Annoroad).

### Quantitative real‐time PCR (qRT‐qPCR)

RT‐qPCR was conducted as described (Dituri *et al*, 2019). RNA was purified with TRIzol reagent. After using a NanoDrop ND‐1000 Spectrophotometer to examine RNA levels, reverse transcription was performed, cDNA was synthesized with a kit from Qiagen, and Real‐time System from Bio‐Rad was used for qRT‐PCR. The internal control was β‐actin. The standard delta CT method was utilized to get the final results. The primer sequences were as follows: β‐actin, (F) 5′‐TAACCAACTGGGACGATATG‐3′ and (R) 5′‐AAACAGGGA CAGCACAGCCT‐3′; TNF‐α, (F) 5′‐CAGGCGGTGCCTATGTCTC‐3′ and (R) 5′‐CGATCACCCCGAAGTTCAGTAG‐3′; WDR6, (F) 5′‐GTAAAACGACGGCCAGT‐3′ and (R) 5′‐TAATACGACTCACTATAGG‐3′; IL10, (F) 5′‐CACAAAGCAGCCTTGCAGA‐3′ and (R) 5′‐GAGCAGGCAGCATAGCAGTG‐3′; p65, (F) 5′‐GCAAGGAATAATGCTGTCCTG‐3′ and (R) 5′‐ATCATTCTCTAGTGTCTGGTT‐3′; UVRAG, (F) 5′‐GAGCTCCTGCGCCTCGCT‐3′ and (R) 5′‐TCACTTGTCGGAACTCCT‐3′; RelB, (F) 5′‐GTGAGGATCTGCTTCCAG‐3′ and (R) 5′‐TCGGCAAATCCGCAGCTC‐3′; IL6, (F) 5′‐AGTCCTTCCTACCCCAATTTC‐3 and (R) 5′‐TGGTCCTTAGCCACTCCTT‐3′; p50, (F) 5′‐GGACAGCAAATCCGCCCTG‐3′ and (R) 5′‐TGTTGTAATGAGTCGTCATCC‐3′;. C‐Rel, (F) 5′‐AAGACTGCAGAGACGGCTA‐3′ and (R) 5′‐TCACCACATTGAGGTCACA‐3′; IL1β, (F) 5′‐GCACTGTTCCTGAACTCAACT‐3′ and (R) 5′‐TTCAATTGGCCTGGTCATGA‐3′; p52, (F) 5′‐GCCACAGAGATGGAGGAG‐3′ and (R) 5′‐CCGAGTCGCTATCAGAGG‐3′.

### Peptide competition and mice treatment experiments

A peptide Pep2‐WDxR‐WT or Pep2‐mut through fusing the peptide (DWTWDVRW) or the peptide (DWTWDVAW) to Pep2 was generated as described previously (Li *et al*, 2014). The peptide synthesis was performed by R&D Company. To investigate the effects of these peptides, HCC cell lines were incubated with 5 μM of Pep2‐control, Pep2‐WDxR‐WT, or Pep2‐WDxR‐mut or for 16 h. Then, co‐IP of these lysates was performed as indicated. To determine the antitumor effect of these peptides *in vivo*, intravenously (i.v.) injected BALB/c mice twice a week with Pep2‐control or Pep2‐WDxR‐WT (5 mg/kg) in phosphate‐buffered saline (PBS, 200 μl) with or without anti‐PD‐L1 from 9 days after tumor inoculation till the time of the killing. The tumor growth and lung metastasis were also evaluated.

### Chromatin immunoprecipitation (ChIP)

The ChIP‐IT kit was purchased from Active Motif and used to perform ChIP following the manufacturer's protocol. 1% paraformaldehyde was added to the indicated cells to preserve protein–DNA interactions. After 10 min, 0.125 M glycine was utilized to quench this reaction. And RIPA buffer was taken to dissolve the samples on ice. Then these protein–DNA complexes were sonicated and sheared into small fragments of 200–500 base pairs. Then a p65 antibody or IgG‐dynabead mixture was incubated with the lysates overnight at 4°C to immunoprecipitate protein‐bound DNA sequences. NaCl was used to reverse crosslinking and protein was digested using proteinase K. Finally, DNA was purified and analyzed by qPCR.

### Immunohistochemical (IHC) staining

Immunohistochemical of the formalin‐fixed samples was conducted on 5‐μm sections. The samples were first dewaxed by xylene, rehydrated from the graded ethanol to water, and blocked using 30 ml/l H_2_O_2_ in methanol to disrupt the endogenous activity of peroxidase according to standard procedures. Then antigen retrieval in a pressure cooker was performed by digging tissue sections in the buffer of sodium citrate (0.01 mmol/l) at power level 7 for 10 min. After being incubated with goat serum for 20 min, the slides were covered using indicated primary antibody (1:100 dilution) or PBS as a negative control in the cold room overnight. Then, added a horseradish peroxidase (HRP)–conjugated secondary antibody (1:500 dilution) for 2 h, followed by incubating the sections using 3,3′‐diaminoberzidine (DAB, 0.02%) and H_2_O_2_ (0.005%), and counterstained them with hematoxylin. Light microscopy was utilized to observe the results. All slides' immunostaining was semi‐quantitatively calculated using a histological scoring system as described before (Keenan *et al*, 2019). All cases were divided into either WDR6‐high or WDR6‐low groups based on the scores.

### Cytokine measurement

According to the manufacturer's instructions, interleukin‐6 (IL‐6), TNF‐α, interleukin‐1beta (IL1β), and interleukin‐10 in the supernatants were measured with a proinflammatory 7‐plex ultra‐sensitive kit and ELISA kit, which were purchased from Thermofisher and MesoScale Discovery.

### Cell culture, shRNA, plasmids, and transfection

Two immortalized human hepatic cell lines (HL‐7702 and L02) were bought from China Cell Bank. The American Type Culture Collection (ATCC) provided hepa1‐6 murine hepatoma cell line, and four human HCC cell lines (HepG2, HCC‐LM3, Hep3B, and MHCC‐97L), and HEK293T cells. H22 cell line was a gift from Dr. Bing Wang (Rutgers University). All cultures were conducted in RPMI1640 medium or Dulbecco's modified Eagle's medium in an incubator with 5% CO_2_ at 37°C, supplemented with penicillin–streptomycin (1%) and fetal bovine serum (10%). All products for culture were bought from Invitrogen (Carlsbad). Mycoplasma contamination was examined routinely using PCR Mycoplasma Detection Kit every month.

For transfection, indicated cells were maintained onto 6‐well plates (1.5 × 10^5^ cells per well). When the cell confluence reached to 80%, indicated plasmids were transfected with Fugene 6 Reagent (Roche). To generate human *WDR6*‐ or *UVRAG*‐ or *TNFa*‐knockdown cells, lentivirus expressing shRNA against them were respectively transduced into indicated cells at multiplicities of infection (MOI) of 150 in the medium without serum but with Polybrene (Sigma, 8 μg/ml). Eight hours later, cultured the cells with fresh DMEM complete medium for another 72 h. Then puromycin. (Sigma) was utilized to establish the stably transfected cells. shRNA against *TNFα #1* (SHCLNV‐NM_013693) and *#2* (sc‐37216‐V) were purchased from Sigma and Santa Cruz, respectively. shRNAs against Human and mice WDR6 (sc‐78080‐V, sc‐155301‐V, and TR30021V) were from Santa Cruz and OriGene, respectively. UVRAG shRNA (sc‐76883‐V and HSH‐613266) were from Santa Cruz and FenicsBio. Plasmids such as CUL4A (#19907), CUL4B (#19922), and DDB1 (#19909) were purchased from Addgene. *WDR6* cDNA was generated by OriGene and then subcloned to pRK5 with a Myc‐tag from addgene. All mutants were constructed using a site‐directed mutagenesis kit purchased from Agilent and verified via sequencing. His‐ub, IκBα‐SR, and UVRAG were already described previously (Li *et al*, 2000; Chiba *et al*, 2009).

### Cell proliferation and migration analysis

Cells in different treatment groups were maintained in the 96‐well plate (3 × 10^3^ cells per well). Eighteen hours later, the proliferative ability of indicated cells was assessed through a CCK‐8 cell from Sigma. Briefly, the cells were incubated with CCK‐8 reagent (10 μl per well). The values of optical density (OD) at 450 nm were read by a spectrophotometer. Triplicate experiments were performed (Savci‐Heijink *et al*, 2019).

For cell migration experiments (Yafune *et al*, 2013), indicated cells (1 × 10^5^) in 100 μl DMEM containing FBS (0.1%) were put into the upper chamber, while the lower chamber included 600 μl DMEM containing FBS (10%) as the chemoattractant. Twenty‐four hours later, cotton swabs were used to detach the cells still on the upper surface. Then 4% paraformaldehyde was used to fix the cells which were on the lower surface. After staining was finished utilizing crystal violet (0.5%), the pictures were taken with a 400× objective lens (Nikon, Japan). Final results were counted and averaged from three independent experiments.

For analyzing the migration of MDSC cells, MDSCs isolated from tumors were replated into the upper chamber of an eight‐micrometer transwell system (BD Falcon). The bottom section contained the conditioned medium with or without TNFα or anti‐TNFα antibody or vehicle. MDSC cells were allowed to migrate in an incubator at 37°C. About 6 h later, the results were observed by flow cytometry.

### Statistical assays

BALB/c nude, BALB/c, and C57BL/6J mice with matched age were randomly assigned to groups. For liver‐specific *WDR6 knockout* (*KO*) mice, littermates were used as controls. No statistical methods were used to predetermine sample size. Animals were allocated into groups randomly, and the surgeries, harvest, and analysis of animals were blind to reduce subjective bias. No inclusion or exclusion criteria were conducted on the animals. No data were excluded from analyses. SPSS 21.0 software from SPSS Inc was utilized to conduct all statistical analyses. As comparing two independent groups, Student's *t*‐test assay was conducted, but the method of one‐way ANOVA was adopted to evaluate two more groups. The correlations between the levels of WDR6 and clinicopathologic variables were explored by using the Cox proportional‐hazard regression model. The analysis of the Pearson coefficient was performed to clarify the correlations between WDR6, pp65, and UVRAG in HCC patients. The Kaplan–Meier method was employed to calculate disease‐free survival (DFS) and overall survival (OS). The triplicate was required for all experiments, and all results were shown with mean ± standard deviation. The difference with *P*‐values less than 0.05 (*P < 0.05, or **P < 0.01, or ***P < 0.001) was significant.

## Author contributions

**Heng Zhang:** Conceptualization; resources; data curation; software; formal analysis; validation; visualization; methodology; writing – original draft. **Gang Chen:** Resources; software; formal analysis; supervision; validation; visualization; methodology. **Xing Feng:** Resources; data curation; formal analysis; validation; visualization; writing – review and editing. **Huiwen Song:** Resources; supervision; funding acquisition; validation; methodology. **Lingbing Meng:** Formal analysis; visualization; methodology. **Yao Fu:** Methodology; project administration. **Jun Yang:** Methodology; project administration. **Zhiwen Fan:** Methodology; project administration. **Youxiang Ding:** Methodology; project administration. **Zhijie Du:** Methodology; project administration. **Jianchao Wang:** Software; investigation. **Li Yang:** Resources; investigation. **Jun Zhang:** Resources; investigation. **Lixia Sun:** Resources; investigation. **Zhigang Liu:** Resources; investigation. **Zhiyong Zhang:** Conceptualization; supervision; funding acquisition; writing – original draft; writing – review and editing. **Quanhai Li:** Supervision; funding acquisition; writing – review and editing. **Xiangshan Fan:** Supervision; writing – original draft; writing – review and editing.

## Disclosure and competing interests statement

The authors declare that they have no conflict of interest.

## Supporting information



Appendix

Source Data for Appendix

Source Data for Figure 1

Source Data for Figure 2

Source Data for Figure 5

Source Data for Figure 6

Source Data for Figure 7

Source Data for Figure 8

## Data Availability

RNA‐, ATAC‐sequencing data presented in this study have been deposited into the Gene Expression Omnibus hosted at the National Center for Biotechnology under the accession number PRJNA848053 (https://www.ncbi.nlm.nih.gov/bioproject/848053). The mass spectrometry proteomics data have been deposited to the ProteomeXchange Consortium via the PRIDE partner repository with the dataset identifier PXD034521 (Project accession number).
