# Supplementary material for: Targeting WDxR motif reprograms immune microenvironment and inhibits hepatocellular carcinoma progression
Source: EMBO Mol Med. 2023 Mar 22;15(5):e15924. doi: 10.15252/emmm.202215924 (PMC10165360; doi:10.15252/emmm.202215924)
Supplement: Supplementary file 1 — Appendix [file EMMM-15-e15924-s003.pdf]

EMM-2022-15924

Targeting WDxR motif reprograms immune microenvironment and inhibits hepatocellular carcinoma progression

Table of contents

Appendix Figure S1 •• **2**

Appendix Figure S2 •• **4**

Appendix Figure S3 •• **6**

Appendix Table S1 •• **8**

Appendix Table S2 •• **9**

Appendix Table S3 •• **11**

Appendix Table S4 •• **41**

Appendix Table S5 •• **43**

Appendix Figure S1

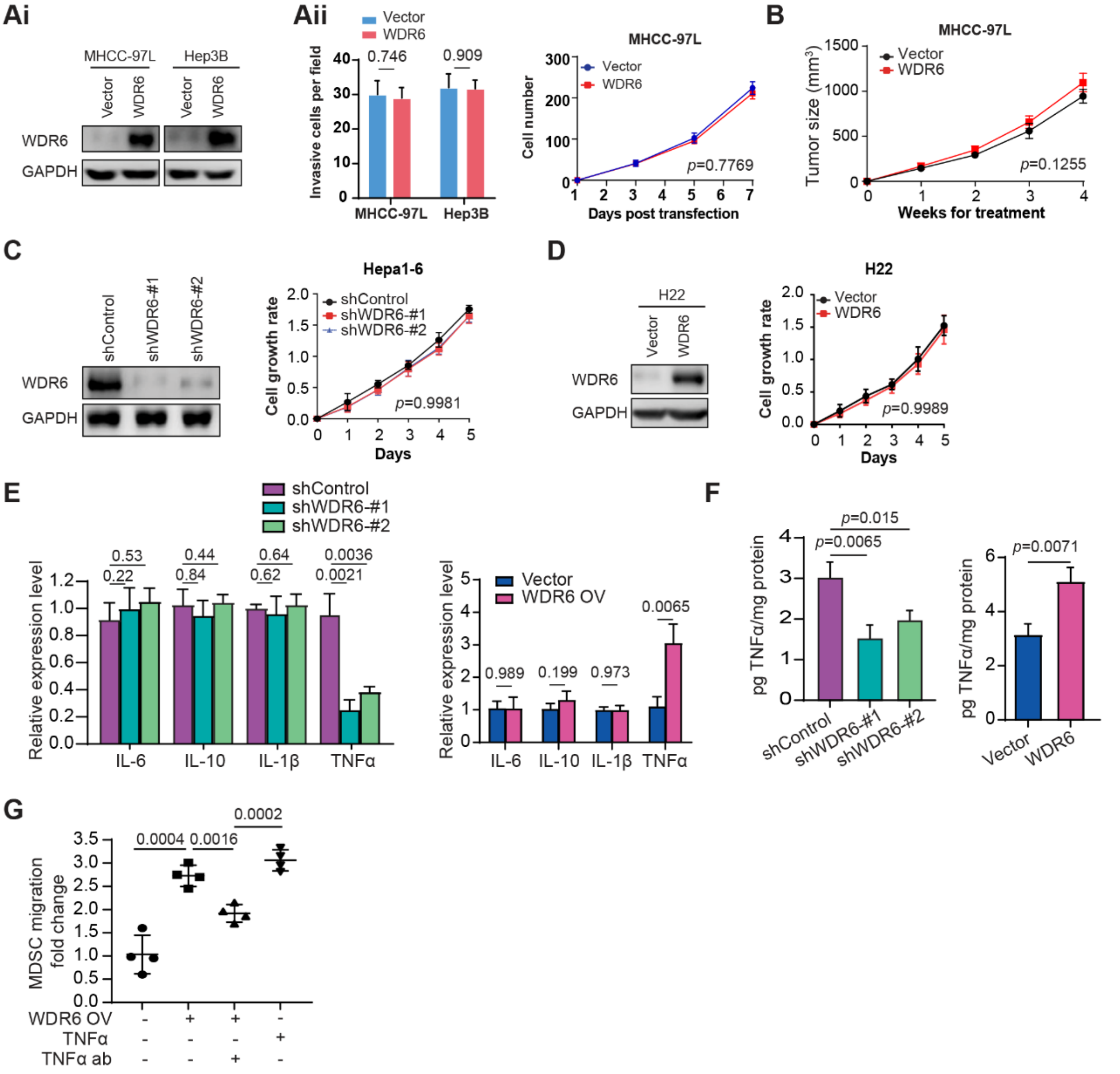

Appendix Fig. S1. WDR6 effects on HCC cell growth. (Ai) Western blots confirmed WDR6 overexpression in MHCC-97L and Hep3B infected with WDR6-expressing or empty vector. (Aii) The impacts of WDR6 overexpression on the invasive and proliferative ability of MHCC-97L and Hep3B cells. (B) MHCC-97L cells were transfected with or without WDR6. Then these cells were subcutaneously implanted into the nude mouse (n=6 per group). Growth rate of the MHCC-97L subcutaneous tumors were shown. (C) Western blots confirmed *WDR6* knockdown in Hepa1-6 cells

transduced with *shWDR6* or *shcontrol* lentiviral vector and its effects on the proliferative ability of Hepa1-6 cells**.** (D) Western blots confirmed WDR6 overexpression H22 cells and its effects on the proliferative ability of H22 cells**.** (E) qRT-PCR analyzing cytokines' mRNA levels in HCC-LM3 and MHCC-97L cells treated with or without *shWDR6* or WDR6, respectively. (F) TNFα ELISA assays were performed in the conditioned medium from HCC-LM3 and MHCC-97L cells with *WDR6* knockdown or overexpression, respectively. (G) The effects of the conditioned medium from MHCC-97L cells with WDR6 overexpression on the migration of isolated MDSCs in the presence of TNFα or TNFα antibody. All results are representative data generated from three independent experiments. Data are presented as mean ± SD. Two-way ANOVA (Aii, B, C, D) and One-way ANOVA (E-G) were used for statistical analysis.

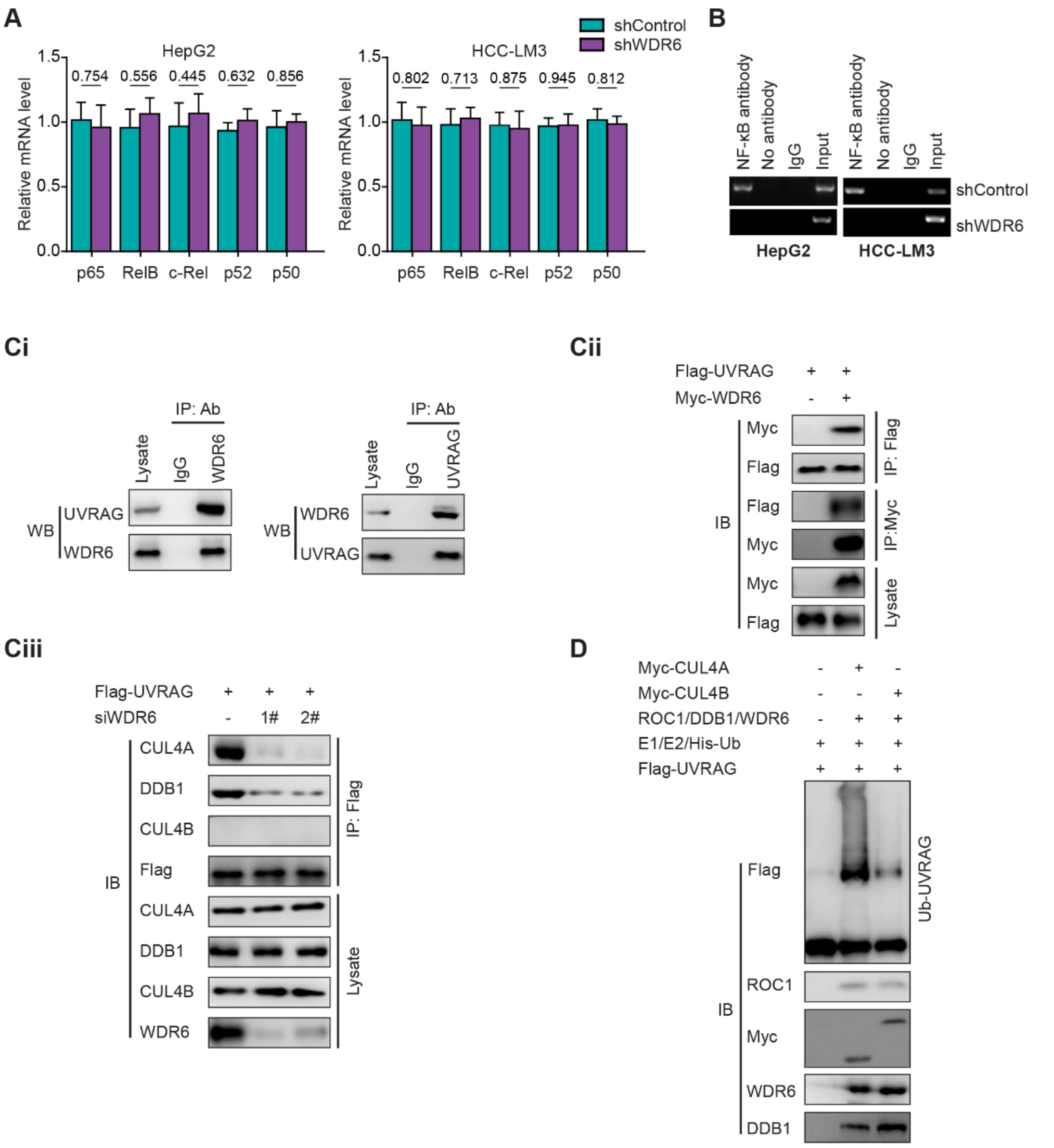

Appendix Figure S2

Appendix Fig. S2. (A) Relative mRNA level of p65, RelB, c-Rel, p52, and p50 with or *WDR6* knockdown in HCC cells. P values were shown. (B) ChIP assays of TNFα locus using HCC cells. (Ci) Co-immunoprecipitation (co-IP) assays for endogenous WDR6 and UVRAG from HCC-LM3 cells treated by MG132. (Cii) MHCC-97L cells were co-introduced with plasmids as indicated. Co-IP was performed with indicated antibody.

(Ciii) Co-IP analysis in HCC-LM3 cells transfected with the indicated constructs. (D) CUL4A but not CUL4B mediated UVRAG ubiquitination *in vitro*. All results are representative data generated from three independent experiments. Data are presented as mean ± SD.

Appendix Figure S3

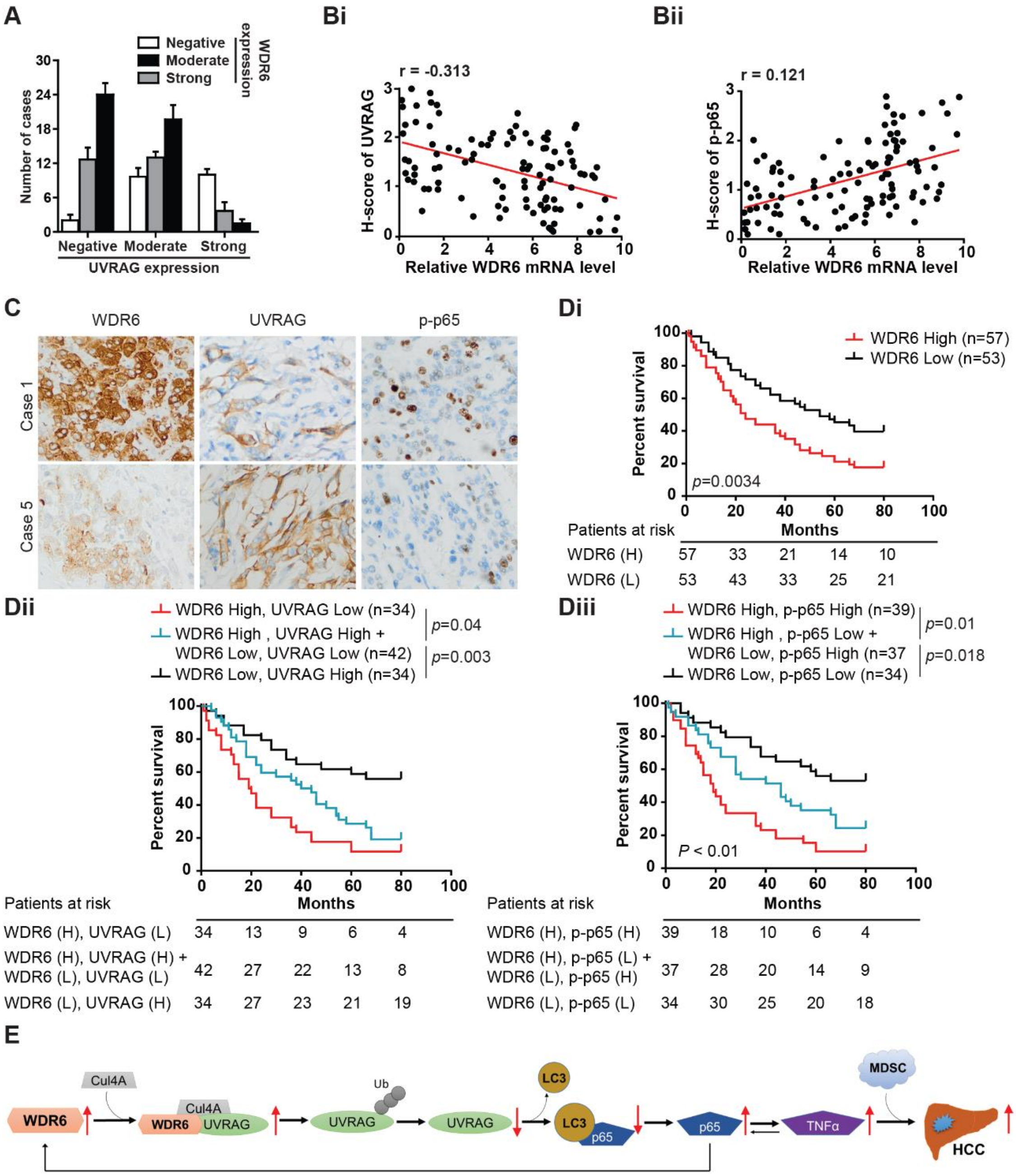

Appendix Fig. S3. Combination of WDR6, pp65, and UVRAG levels indicates an important prognostic value for HCC. (A) Tissue sections were analyzed from 110 HCC patients' specimens. Immunostaining of the sections was performed using indicated antibodies, which reveals an inverse relation between WDR6 and UVRAG expression levels. (Bi-ii) Correlative analysis between WDR6 and UVRAG or pp65 in

human HCC. (C) Representative photographs of 2 cases showing UVRAG, WDR6, and pp65 expression in HCC. (Di-iii) The OS of HCC patients with the profiles of WDR6, UVRAG, and pp65. (E) Schematic model showing the impact of WDR6/Cul4A/UVRAG/p65 axis on HCC growth. All results are representative data generated from three independent experiments. Data are presented as mean ± SD. One-way ANOVA (A), pearson coefficient (B), and long-rank (Mantel-cox) test (D) were used for statistical analysis.

Appendix Table S1. RNA sequencing of 4 pairs of HCC patients.

Normal tissue				Tumor tissue				
Patient 1	Patient 2	Patient 3	Patient 4	Patient 1	Patient 2	Patient 3	Patient 4	
4.27327	9.283425	18.29926	6.29228	41.2728	26.16345	32.93725	48.29115	FABP5
4.06395	5.6413016	1.3857195	6.15509895	83.5848596	101.3743016	35.803265	46.848133	AURKA
8.9985665	15.21236	11.01215	29.569856	33.65236	46.32584	62.541252	52.559544	SOX9
1.18279475	1.829925	2.56242275	1.6661275	26.0579262	16.3256295	11.292285	31.299223	LEF1
6.12815	11.81075	25.6273	21.10615	56.12615	91.1626	46.1626	58.28139	TKT
21.99138	31.3275	13.9968	24.75126	61.9536	55.3275	37.32912	42.7287	ATPIF1
22.8376	7.908	16.9792	9.70048	33.8032	57.0036	42.65928	64.4468	WDR6

Appendix Table S2. Relationship between Intratumoral WDR6 Expression and Clinicopathologic Features (n=201)

variable	Relative WDR6 Expression		P-value
	Low (n=99)	High (n=102)	
**Age**			NS
≤60	52	58	
>60	47	44	
**Gender**			NS
Male	92	89	
Female	7	13	
**HBsAg**			NS
Positive	89	90	
Negative	10	12	
**Tumor size**			**0.003**
≤5cm	58	38	
>5cm	41	64	
**Distant metastasis**			**0.004**
Yes	6	20	
No	93	82	
**Tumor stage**			NS
I-II	73	81	
III-IV	26	21	
**Intrahepatic metastasis**			NS
Yes	22	31	
No	77	71	
**AFP(μg/L)**			NS
≤20	21	27	
>20	78	75	
≤1000	49	59	
>1000	50	43	
**Anti-HCV**			
Positive	4	6	NS

Negative	95	96	
**Liver Cirrhosis**			NS
Yes	63	58	
No	36	44	
**Vascular invasion**			**0.023**
Yes	49	67	
No	50	35	
**Edmondson**			
I-II	16	11	NS
III-IV	83	91	

Note: HCC patients were divided into WDR6 high group and low group according to the analysis of IHC detection. Differences among variables were evaluated by χ^2 or Fisher's exact χ^2 -test.

Appendix Table S3. RNA sequencing of HCC tissues with high and low WDR6 levels.

gene_name	WDR6 high S1_fpkm	WDR6 high S2_fpkm	WDR6 high S3_fpkm	WDR6 low S1_fpkm	WDR6 low S2_fpkm	WDR6 low S3_fpkm	LOG2FC
**CDK13**	3.287838	3.37627	3.296495	661.1323	581.2067	863.5697	7.723994
**IQCN**	0.100993	0.088856	0.096337	0.959418	1.426841	1.904833	3.90632
**HSPA6**	1.31012	0.956948	1.190202	8.073689	9.408453	15.07936	3.235462
**SMG1P7**	1.192894	1.004337	1.183645	4.152145	3.969006	6.115774	2.074169
**RPSAP47**	7.204379	7.327817	6.753021	37.66573	14.91085	16.66483	1.701783
**RELB**	1.998244	1.833701	1.307543	4.582156	5.268954	5.521265	1.580643
**MAP2K7**	13.87188	11.89025	11.50967	33.26556	36.22512	38.22562	1.53108
**DHRS2**	0.9103	0.929171	1.21379	2.895367	3.17801	2.580959	1.503072
**NFKBIA**	8.306018	7.63109	7.71121	20.36547	23.25412	22.23156	1.477471
**JUNB**	30.13236	23.66304	24.6147	66.32585	67.25546	69.33559	1.371778
**FOS**	3.81446	2.92186	3.009453	7.956555	8.584567	8.655663	1.370391
**RANBP3**	12.95407	12.24782	12.25372	33.26552	29.66659	31.25842	1.3304
**MT2A**	25.13709	22.34418	21.96177	71.41536	46.33541	53.25156	1.300114
**RELA**	14.38852	12.25378	12.29834	30.36558	32.25155	33.26954	1.300054
**TNFAIP3**	2.745474	2.50028	2.522857	5.988556	6.458212	6.655845	1.298041
**NFKB2**	12.62621	11.89588	10.45297	26.25415	28.56951	30.26552	1.282649
**EGR1**	3.040396	2.755766	2.655712	6.39585	7.258114	6.528156	1.255734
**TUBBP1**	2.381439	2.106683	1.821593	8.59458	3.108625	3.345304	1.253974
**HMOX1**	66.0868	59.72022	50.25348	114.2327	126.6462	165.3883	1.206357
**POLE4**	13.39246	11.1851	10.41347	26.32583	25.58512	27.96582	1.190791
**FADD**	10.08355	7.903734	8.394549	22.22511	18.22451	19.25585	1.178319
**MYBL2**	154.4172	134.0256	132.2004	312.2583	311.2582	320.5624	1.16631
**LSM12P1**	25.21915	21.76897	23.32143	59.74497	61.14326	36.15767	1.159394
**SLC25A4**	9.680071	9.217733	8.683931	22.52126	18.59225	20.26585	1.15404
**PTGES3P1**	8.268753	7.002029	8.124844	29.94248	11.61599	10.45032	1.152517
**E2F2**	20.03152	18.01984	17.63892	44.25154	39.26855	40.26885	1.152385
**PTGS2**	1.761607	1.16998	1.431001	3.26999	3.011582	3.365951	1.144975
**RHOB**	68.93939	65.89328	63.01692	119.458	152.4965	160.5477	1.128303
**SPATA5L1**	4.375461	4.575101	4.116751	8.245155	9.316829	10.6992	1.112859
**IL18R1**	1.716035	1.583365	1.701738	3.362596	3.56229	3.695112	1.086455
**IRF1**	1.147466	0.90022	0.927208	2.36699	2.022356	1.895555	1.079052
**CRTC2**	19.0427	19.34267	19.5441	40.25822	39.26821	40.28156	1.048355
**PPP1R3F**	2.29517	2.380036	1.690611	3.478267	4.506704	5.039845	1.032845
**TCIM**	1.55578	1.454705	1.165356	1.787035	3.534569	3.155619	1.021525
**ATF3**	37.69367	32.98692	32.53515	51.97807	74.63663	80.45267	1.004437

**SMAD7**	12.41719	12.07829	11.67819	16.9065	25.55001	29.62301	0.99465
**ATF4**	288.6888	267.2467	255.9829	523.2551	552.2251	536.9996	0.989874
**MICAL2**	0.252746	0.241708	0.222748	0.474041	0.477941	0.445054	0.961916
**WEE1**	9.93837	9.452554	9.836151	19.09151	16.99371	20.7935	0.960584
**HIST1H1C**	64.28247	58.54684	66.62065	146.3834	106.3378	109.8915	0.936613
**KLF6**	6.0659	7.370914	7.354861	11.68474	13.54056	14.43839	0.931813
**CD40**	4.375517	5.505821	4.397111	8.56549	9.255846	9.366589	0.929127
**HSP90AA2P**	1.330326	1.598547	1.766943	4.047218	2.6162	2.263674	0.926814
**AC078778.1**	3.018751	3.785907	4.919247	8.943857	6.473094	6.763304	0.919823
**CREB3**	24.44586	21.61865	22.79966	45.62447	40.25965	44.21583	0.917795
**INSIG1**	49.40083	59.85172	52.21124	105.8714	97.06052	100.7196	0.911206
**IL1B**	0.143332	0.124447	0.14242	0.254893	0.268545	0.245565	0.906664
**ARHGEF7**	4.311366	4.638222	4.443797	8.329854	8.02546	8.562699	0.895669
**MIR1244-2**	48.7262	52.37914	59.13683	159.6258	61.25371	73.77816	0.878786
**HSPB8**	3.64625	4.35583	4.239736	7.520721	6.748322	8.236024	0.878432
**ALMS1-IT1**	1.422972	1.993049	1.591965	2.648232	3.177075	3.306147	0.866614
**KLF2**	1.575944	1.421339	1.791698	2.293315	2.846965	3.562375	0.861737
**INHBA**	1.759453	0.840522	0.998498	2.128427	2.124727	2.27995	0.860384
**USP42**	5.040785	4.667197	5.187442	9.689567	7.76207	9.278504	0.843598
**IRAK1**	132.1509	110.4005	102.1055	200.6922	206.2516	211.3622	0.84316
**RPSAP15**	33.88732	30.42932	32.36578	78.06024	46.03105	45.34459	0.809414
**FOSL2**	4.009536	3.4992	3.536332	5.327325	6.308997	7.709713	0.808636
**AC016739.1**	17.57876	16.75169	15.33678	43.74868	19.25467	23.8299	0.805953
**HMGCS1**	9.288181	10.04238	10.6529	18.58877	17.03919	16.72027	0.803974
**TRIB1**	4.066159	3.838198	4.464735	7.752496	6.594873	7.235594	0.803153
**PIK3AP1**	0.773253	0.571701	0.612083	1.14587	1.13571	1.095221	0.786985
**NKAIN1**	1.349443	0.960248	1.107129	1.584563	2.329109	1.95866	0.781279
**RPS29**	14.69046	13.43652	13.41832	31.09988	22.38511	17.80458	0.779006
**MAP2K1**	32.42487	28.72214	28.1052	50.25923	49.56822	53.26584	0.778451
**RPP40**	4.410423	4.320263	4.022779	9.622715	5.559051	6.626479	0.773984
**DCLK1**	0.249533	0.273983	0.248456	0.530472	0.462112	0.32719	0.773671
**KCNJ14**	1.090646	0.937083	0.843258	1.308688	1.747794	1.832637	0.768028
**MSL2**	8.426582	8.570377	9.078277	14.92541	13.49609	15.97844	0.767877
**LINC00641**	3.332379	3.932666	4.081423	7.65613	5.741092	5.821112	0.76024
**NLN**	2.894739	3.558366	3.399224	6.335957	5.1493	5.102915	0.751618
**JUN**	157.989	160.1138	146.7907	167.7677	296.0581	306.7196	0.72898
**GCLM**	6.672721	6.737421	6.410606	14.43796	8.330903	10.08175	0.728909
**FOSL1**	4.056597	2.518228	2.289952	3.623044	4.923703	6.079282	0.722382
**C11orf96**	5.262651	3.244587	2.812928	6.939341	6.460974	5.23332	0.719014
**CYP1B1**	1.045319	1.245715	0.923803	2.186144	1.261295	1.818467	0.711936
**PFDN4**	12.15181	10.46945	11.00657	24.41768	13.95387	16.63141	0.709853
**SNRPEP4**	20.14665	15.9578	18.64876	35.05992	25.90091	28.50769	0.708437
**ODC1**	75.928	65.2389	68.71001	130.2698	97.81365	113.724	0.70364

**GFRA1**	0.676318	0.915648	0.760857	1.249811	1.28002	1.29991	0.702854
**LPP**	0.596657	0.633724	0.581924	1.060654	0.929851	0.956903	0.701621
**HSP90AB1**	343.7871	368.5097	355.4834	613.3957	526.6964	593.2898	0.698975
**LATS2**	3.33467	3.000836	3.130321	5.062884	4.506121	5.792701	0.698538
**CEBPB-AS1**	2.094772	2.198695	2.148047	2.502389	3.508696	4.389939	0.691254
**GADD45B**	13.24904	11.09292	11.4461	17.16919	19.90188	20.42497	0.683984
**SYCP2**	0.611944	0.68856	0.681925	1.276927	0.819105	1.088141	0.68365
**PHLPP1**	2.382525	2.753468	2.729904	4.193614	3.868819	4.538282	0.679822
**HSPD1P1**	2.35676	3.001156	2.861208	4.72273	4.472816	3.965657	0.679235
**AC010343.1**	42.44729	45.19198	46.54677	63.62623	56.65826	94.22887	0.676833
**PRKCB**	0.3269	0.316653	0.381829	0.536051	0.530383	0.57258	0.676668
**PQLC3**	2.568261	3.236615	3.081682	5.085615	4.143156	4.975231	0.676601
**NDUFAF4**	8.851834	7.786678	7.395168	18.88267	8.906154	10.59844	0.67557
**IQCH**	1.134155	1.210578	1.228259	2.042038	1.711257	1.95282	0.675376
**HIST1H2BD**	8.533777	6.635285	7.08732	11.41158	10.66708	13.43403	0.674115
**SEC61G**	54.32523	50.64208	50.17343	110.2617	66.10793	70.87235	0.672346
**PNO1**	9.860332	9.173545	8.871418	17.42507	12.63941	14.40028	0.672124
**WNK4**	1.584118	1.253423	1.282335	1.891128	2.354898	2.306794	0.669515
**RPL26P19**	15.53485	15.73605	16.00646	31.45787	19.67526	24.00831	0.668459
**TSC22D2**	3.597888	3.589489	3.987879	5.531036	5.327979	6.897111	0.668009
**LNP1**	2.301982	1.827367	1.960572	3.086645	3.292971	3.281488	0.665764
**STXBP6**	1.18269	1.284243	1.300879	2.346889	1.624666	1.981377	0.659873
**HSPH1**	12.48671	13.58282	13.89016	24.81565	17.4829	20.73731	0.657627
**FAM72B**	5.437833	6.097549	6.338496	8.062741	9.900271	10.21211	0.656569
**SLC16A3**	0.50567	0.642505	0.495099	0.730821	0.813354	1.045772	0.656349
**AL392172.1**	7.887158	6.327372	6.269574	12.05287	9.908951	10.30191	0.655409
**AC099850.3**	16.47382	16.93724	18.95598	34.89609	20.13496	27.39843	0.654501
**IDI1**	9.889623	11.08426	10.08716	19.03887	14.05924	15.78831	0.654327
**BOLA3**	9.701899	8.206583	7.306962	15.13042	12.62901	11.7527	0.647988
**GEM**	4.34316	3.572879	3.829852	5.878627	5.796976	6.729315	0.647935
**GABBR2**	0.576597	0.600191	0.543077	0.799322	1.011541	0.871794	0.641367
**SMIM19**	3.090759	2.902543	3.728793	5.808744	4.658837	4.694986	0.641175
**SHISAL1**	1.133876	1.15411	0.999299	1.877375	1.495918	1.744558	0.638642
**AP001324.1**	14.27287	14.16267	16.24488	29.91956	17.6612	21.85068	0.635946
**GNG2**	0.660869	0.704662	0.711962	1.286098	0.999146	0.941131	0.635071
**MT1X**	8.426283	6.422689	6.490885	8.453534	13.74626	10.89145	0.632899
**S100A11**	61.3428	64.21481	61.47118	102.4729	87.26014	100.2078	0.632499
**SLIRP**	16.24802	14.82318	14.99942	31.58191	19.02161	20.7108	0.630345
**CPEB2**	2.535044	2.875068	2.727798	4.132743	4.014999	4.443509	0.629691
**IL6R**	5.191943	4.358607	4.735259	7.082071	6.969465	8.029353	0.628216
**ITGA9**	0.603148	0.672379	0.671227	0.876816	1.068284	1.053331	0.623136
**FAM72D**	3.023789	3.286198	3.347923	4.870652	4.812364	5.136778	0.617742
**GTF2F2**	4.516449	5.950088	4.788242	8.995064	6.573258	7.829473	0.617111

**CAP2**	2.694424	3.554738	3.212734	4.930753	4.294665	5.256899	0.614091
**HSPB1**	62.03266	60.44209	58.17935	76.37441	100.7285	99.08359	0.612413
**SLC16A9**	4.800932	5.403188	5.127123	9.297331	6.485861	7.623562	0.61045
**SLC43A2**	1.638738	1.913245	1.409354	1.993318	2.733847	2.844646	0.609909
**VTA1**	9.532636	9.496931	9.52662	18.15517	11.9411	13.46937	0.609387
**RAPGEF2**	2.74388	2.979761	3.198629	4.551847	4.348103	4.69234	0.607306
**DOP1B**	0.892755	0.919725	0.878935	1.345323	1.297317	1.452477	0.60554
**MGST1**	1.714785	1.731959	1.511261	3.513061	1.846043	2.184333	0.605463
**SLC30A1**	10.36972	7.593787	7.676447	14.52611	10.61838	13.84528	0.604702
**AL355032.1**	22.45799	19.54789	18.92587	28.3361	27.36091	36.92914	0.604225
**RABL3**	1.981619	2.522529	2.598151	3.940873	3.17179	3.68182	0.603936
**ZBTB10**	6.367198	7.232757	6.82375	10.31103	9.916172	10.77989	0.602354
**DUSP1**	27.3822	25.63504	24.91848	32.11655	43.86157	42.29172	0.601726
**SRGAP1**	0.208211	0.245858	0.223437	0.36322	0.325162	0.338738	0.600298
**METTL5**	12.26203	11.34125	12.00445	20.70554	15.94838	17.32419	0.600184
**TIPARP**	4.900836	5.453001	5.467983	8.886523	7.193207	7.885592	0.599033
**PRR11**	8.882379	9.684685	11.06654	15.72541	12.9042	16.2327	0.59827
**AKAP7**	1.994581	1.929075	2.137429	3.997501	2.441357	2.715173	0.594831
**NRAS**	23.2318	23.17064	26.89798	45.41477	29.89253	35.35939	0.594328
**HSP90AA1**	182.545	206.3407	205.4857	345.7023	257.1689	292.7431	0.591513
**MSMO1**	15.54887	15.86867	15.1399	25.73122	20.85904	23.42979	0.588756
**FOPNL**	12.83695	11.74541	13.70192	23.55657	14.98017	18.93542	0.586111
**GLO1**	155.277	146.2125	143.3593	280.0769	178.3154	209.4023	0.586089
**RPF2**	13.90746	12.27973	12.59165	22.55385	16.61699	19.02184	0.585568
**AC144652.1**	3.666091	4.323165	3.366726	4.565993	6.439665	6.027773	0.584917
**CDKN3**	14.50184	13.7803	13.30483	24.54767	18.18836	19.61919	0.584379
**SNAI2**	6.770164	8.127818	8.319482	12.35676	10.9564	11.46484	0.582965
**RPS26**	91.25142	90.14471	91.42961	183.8981	98.88513	125.7984	0.582644
**RWDD4**	4.529214	4.626427	3.621492	8.202961	5.190138	5.730292	0.581774
**PSMA3**	24.61361	24.43141	25.23452	48.49263	28.59406	33.99675	0.580607
**DDX10**	2.517167	2.562755	3.052335	4.619012	3.869595	3.667873	0.579998
**FAM72A**	2.121225	2.591452	2.634173	3.934162	3.476359	3.558027	0.578175
**SF3B6**	99.04505	91.66392	95.62747	154.5624	128.503	143.9781	0.576672
**ZCCHC2**	1.517487	1.581058	1.818012	2.651332	2.131286	2.548	0.576286
**UBC**	93.35999	91.10003	88.15257	157.3919	110.4372	137.1413	0.570964
**BACH2**	0.918257	0.791803	0.806529	1.273636	1.022805	1.437956	0.569405
**NME1**	15.99442	15.22876	14.08214	28.14584	20.2488	18.80792	0.568836
**ZDHHC14**	0.964312	0.973914	0.939673	1.390291	1.360127	1.499152	0.562301
**DNAJC25**	9.008976	9.154625	9.156256	15.1506	12.02813	13.16026	0.562225
**WNT3**	4.863505	4.820309	4.79186	7.498972	6.690167	7.181884	0.562026
**LSM3**	16.90501	17.33716	16.78202	31.29201	21.1891	22.82293	0.561546
**MRPS35**	18.34971	16.37548	18.51516	30.16145	22.43075	25.9111	0.560234
**LIN52**	4.978037	5.890416	6.031174	8.859942	7.476645	8.575739	0.559868

**SPATS2L**	1.743223	1.944225	1.93914	2.88219	2.434076	2.967647	0.558052
**SPRED1**	2.471283	1.653311	1.927078	2.998769	2.749564	3.157237	0.557374
**GNG12**	15.11596	12.23398	12.99276	24.14637	16.08572	19.09506	0.556384
**HSPA1B**	59.58411	69.64034	59.4673	73.09597	93.64967	110.6965	0.556156
**PAK1IP1**	13.49759	11.2257	12.00316	22.11697	14.71204	17.14368	0.55541
**AL353625.1**	1.889732	2.132974	1.830764	2.788221	2.953096	2.857826	0.554901
**KPNA2**	73.62965	81.17516	78.97467	129.6826	96.11506	117.4439	0.554076
**KLF7**	1.864556	1.858084	2.023281	2.668672	2.81556	2.935403	0.551219
**UTP20**	3.456702	3.484922	3.668573	6.683909	4.226923	4.619617	0.549648
**GAS2L3**	2.148402	2.827127	2.568161	4.447593	3.175363	3.402618	0.547512
**CTGF**	3.4624	3.294475	3.732563	4.946462	4.835748	5.525727	0.545342
**HAUS2**	5.397685	4.961311	4.977448	8.065485	6.437242	7.845874	0.54322
**C3orf14**	5.442611	6.194386	6.380085	11.80008	6.849145	7.578682	0.541737
**MEX3C**	3.336626	2.809738	3.813439	5.068481	4.277827	5.152331	0.541728
**DUSP8**	5.599829	4.062116	3.820906	6.004087	6.067533	7.546672	0.541074
**SNX10**	8.493348	8.177441	8.472723	15.95037	9.938824	10.68466	0.540626
**OAS3**	0.941187	0.959863	1.052766	1.657177	1.202206	1.436673	0.540433
**GADD45A**	42.07357	36.4784	33.45887	37.82201	64.43018	60.49068	0.538956
**PTTG1**	35.62029	35.4516	32.9878	53.71326	45.84297	51.17487	0.534566
**KCMF1**	4.956617	4.765607	4.659453	7.966364	6.309022	6.517085	0.531829
**NFE2L2**	5.306671	4.453544	5.818628	9.82487	5.592694	7.086212	0.530579
**RPF1**	26.06555	25.44314	24.03695	42.45537	33.35144	33.30394	0.530373
**ME1**	7.163377	7.949861	7.900423	14.24342	8.60136	10.39058	0.530228
**DNPEP**	6.667706	6.034634	6.373252	8.28736	9.953279	9.292169	0.529424
**NQO1**	2.4888	2.328626	2.195177	3.71717	3.089754	3.313862	0.529299
**DCAF4**	4.313526	3.9197	4.282126	6.982459	5.830682	5.248312	0.529215
**MRPL47**	14.41399	13.03045	15.28781	24.6827	17.16738	19.78922	0.528525
**CHORDC1**	6.129156	5.709323	5.739779	9.891142	7.169675	8.286543	0.528043
**TXNL1**	2.569399	2.409903	2.649966	4.128804	3.174231	3.695932	0.527752
**PDPN**	8.219928	8.846065	8.647369	13.42056	11.18775	12.46084	0.527701
**PARVA**	1.182867	1.042843	1.156466	1.858184	1.484612	1.529666	0.526699
**SAT1**	43.81902	45.15773	46.93513	63.6426	68.24265	63.80257	0.525882
**TTC27**	5.391754	5.856361	5.020202	9.179936	6.466035	7.774347	0.525696
**HSPA8**	126.4204	136.3224	125.2234	247.4186	134.4026	176.6991	0.525679
**POLR2K**	27.44841	23.64426	24.81695	44.53696	31.03331	33.59015	0.524095
**PRKCE**	1.11596	0.972166	1.117017	1.470266	1.535702	1.60164	0.523629
**AC016705.2**	1.331086	1.416959	1.574976	2.030777	1.875591	2.304552	0.522767
**DHRS7**	17.7498	17.70125	16.43994	27.25024	23.22978	24.064	0.522608
**BICRA**	4.694506	4.26559	4.305362	4.280327	7.1389	7.635511	0.522475
**ETS1**	1.117305	1.071894	1.09786	1.500676	1.481699	1.738365	0.522216
**RPP30**	8.519716	7.443196	7.865334	14.354	9.12539	10.74102	0.522184
**MRPL1**	8.926135	8.108098	7.216498	13.3437	10.44141	11.04061	0.522125
**PHYH**	4.823439	5.507925	5.686087	8.632767	6.850116	7.498446	0.520818

**STK3**	0.971682	1.070502	0.987084	1.770113	1.300526	1.271891	0.519566
**SUB1**	12.25121	11.93755	11.77523	21.72632	14.48964	15.33402	0.519419
**MTMR2**	5.886293	6.366561	6.867277	11.08693	7.834011	8.44888	0.517494
**CABLES1**	3.234473	3.461333	3.551846	4.60774	4.863465	5.189289	0.51664
**LMCD1**	0.915379	0.886207	0.912774	1.330587	1.170037	1.382121	0.516465
**BLVRA**	12.85456	11.66046	12.54273	18.82955	16.98254	17.15596	0.515347
**COPS2**	5.229353	6.008204	5.806688	10.67203	6.305302	7.382968	0.515247
**JAZF1**	2.127696	1.913518	2.128134	3.275927	2.76391	2.763804	0.512982
**TXNRD1**	21.75893	19.71373	19.50936	30.26192	25.09179	31.59765	0.511825
**PFDN1**	24.83609	22.11976	22.18979	36.88122	27.87566	33.78696	0.511127
**DTNB**	1.826733	2.024872	2.042383	3.068847	2.644311	2.686233	0.51104
**ACOT7**	12.6703	12.74746	11.72898	18.13356	17.59887	17.11654	0.50864
**ME2**	2.392764	2.698843	2.752605	4.273758	3.373982	3.507704	0.508047
**C15orf41**	1.106247	1.018076	1.19094	1.867498	1.439308	1.407256	0.507847
**TTC7B**	1.811776	1.837757	1.832727	2.910395	2.388983	2.491951	0.507099
**SRM**	65.73736	55.55359	53.96661	93.51467	79.76521	75.75208	0.506854
**PITPNC1**	1.42357	1.543981	1.669216	2.159017	2.215794	2.210498	0.506132
**MXD1**	1.909832	2.17723	1.791223	3.038968	2.459408	2.847882	0.505734
**PSMD1**	15.81993	16.94403	17.39248	25.63085	20.18793	25.38925	0.505605
**FDFT1**	12.41187	12.61583	12.62108	18.9453	16.84236	17.65654	0.50543
**TNFRSF10D**	12.59484	11.89432	12.0141	19.99248	14.48777	17.33602	0.50538
**PIBF1**	5.303931	5.898563	5.671058	9.632616	7.334707	6.977009	0.504921
**USP31**	2.575685	2.3334	2.458391	3.470398	3.122508	3.847097	0.50288
**YWHAZ**	60.15739	58.41759	62.12227	97.23134	74.22286	84.43798	0.501962
**ANKRD18B**	1.900442	2.192754	2.341301	3.555676	2.774129	2.781232	0.501788
**ISOC1**	26.00016	25.88453	25.52408	44.18634	32.13615	33.25189	0.501342
**MRTO4**	28.59309	21.54147	23.40599	39.80581	30.52188	33.74001	0.500911
**FARSB**	19.5381	18.60304	19.80205	29.65888	26.44557	25.73081	0.498083
**LDLR**	5.002178	4.925521	4.530442	6.800298	6.891392	6.72323	0.497742
**CORO1C**	14.30048	14.92971	15.27603	22.89737	17.31601	22.61421	0.497391
**FBXW7**	2.769728	2.693222	2.897159	3.417513	4.187033	4.184025	0.495795
**LPIN1**	2.157487	2.468759	2.464702	3.622455	3.042483	3.330289	0.495261
**DBF4**	7.09255	7.388744	7.44669	13.50606	8.412725	8.969361	0.49428
**CYB5B**	13.61226	13.90406	13.36275	23.31233	16.14478	18.06962	0.49287
**UBE2T**	46.60517	39.13009	41.79784	71.24465	52.46451	55.69145	0.492313
**NUAK1**	3.028874	3.164314	2.969751	4.705173	3.535398	4.648881	0.492309
**LARP1B**	4.621489	4.119628	4.712008	6.312115	6.32764	6.284542	0.492298
**PSMD6**	12.14416	11.90493	11.80267	19.49567	14.35133	16.56046	0.491593
**RWDD1**	8.217016	7.092718	8.163483	14.48024	8.469431	10.0434	0.491147
**UFM1**	11.62894	11.79177	11.90377	20.27027	14.17611	15.16632	0.490041
**RIOK3**	9.363094	9.310343	9.804947	14.21508	12.4697	13.31166	0.490005
**DDX21**	45.9889	46.70711	49.01202	74.11821	59.81578	64.97529	0.489189
**PPP3CA**	6.646841	7.579803	7.079619	10.15884	9.910167	9.829241	0.488783

**DARS2**	18.98205	20.51858	21.03292	32.36137	24.22225	28.32171	0.48812
**POLR3GL**	6.228377	6.639219	5.61276	8.711387	8.707383	8.480953	0.486944
**ALAS1**	38.27749	32.03988	29.03617	46.8249	43.34439	49.0197	0.486402
**NAA15**	6.918464	7.929877	7.936059	13.08249	8.951881	9.874651	0.485918
**CAMK4**	0.848061	0.867893	1.011197	1.402649	1.210034	1.201316	0.48391
**TUBA1C**	47.40046	45.75877	43.11256	77.90252	52.02728	60.53288	0.483021
**PCGF5**	4.104898	4.102656	3.938983	7.217971	4.863444	4.893778	0.482883
**LDLRAD3**	4.502228	5.240116	4.674367	7.011984	6.259259	6.870426	0.482441
**STIP1**	64.51001	58.76821	56.24091	80.8328	82.53023	87.28306	0.481514
**EIF4E3**	2.590192	2.70466	2.7162	4.147082	3.243787	3.788748	0.480807
**RPS23**	69.07745	62.39203	64.17855	100.7014	89.5762	82.74436	0.480756
**GATA6**	11.01423	9.451813	10.51755	14.71717	12.25355	16.226	0.479417
**SHISA2**	13.04634	15.14811	14.55386	21.21983	18.84822	19.52045	0.479167
**RPL24P4**	35.39862	30.81091	34.77188	44.09555	39.66014	56.9493	0.478584
**GLRX3**	20.95254	19.44775	20.34614	32.49125	26.11664	26.02714	0.478455
**CETN2**	18.87346	18.20752	17.28968	29.21357	21.99977	24.45451	0.476852
**MYO1B**	4.662709	4.517648	4.912099	7.463979	5.551081	6.594398	0.476627
**GABPB1**	2.651178	2.458344	2.205828	3.946325	2.941152	3.287031	0.47596
**ATP5F1E**	14.65914	13.81341	14.25332	19.98539	20.34937	19.0895	0.475942
**MRPL33**	20.27997	16.38403	15.70052	26.75626	21.6109	24.4489	0.475667
**ZNRF2**	2.681429	2.467128	2.755828	3.599903	3.873933	3.517628	0.475658
**TXN**	233.8594	208.4264	201.5085	376.6946	239.6706	278.8544	0.475642
**AHSA1**	48.83378	46.06273	42.50781	57.85244	62.33238	70.83495	0.475295
**RBMX**	53.24398	57.73001	59.07087	90.47212	67.04394	78.83071	0.47499
**KIF20A**	12.16462	13.95269	14.29299	20.75427	15.86483	19.53477	0.474662
**RUNX1**	1.617001	1.181986	1.347656	2.178019	1.795483	1.787451	0.474364
**TANC2**	1.199989	1.377944	1.446249	2.010127	1.644929	1.934845	0.474127
**AK6**	7.047011	7.781814	5.526953	10.60554	8.594844	9.073286	0.474021
**ALKBH1**	5.058053	5.253392	4.403124	7.803421	5.665761	6.964711	0.473719
**SQLE**	21.99025	25.2981	21.14018	36.04942	28.20249	30.76859	0.473641
**HIGD1A**	36.9797	33.96676	34.06368	63.7044	38.77458	43.30673	0.473321
**SKA1**	10.21643	9.47622	9.640728	16.40091	10.75699	13.51964	0.47169
**CALM1**	29.84773	28.46954	29.04833	48.53558	34.30538	38.29824	0.471529
**CAMK2D**	2.953153	3.058264	2.700809	4.171193	4.023301	3.88463	0.471403
**VAMP3**	25.13548	25.48428	26.66364	45.31114	28.41039	33.41728	0.471251
**AIDA**	9.175021	9.134527	8.751969	15.17303	10.43621	11.88182	0.470304
**CFL2**	10.92032	10.23287	10.74846	18.96576	11.84782	13.38008	0.470209
**TMA7**	23.83064	19.65721	21.25303	34.72133	29.51559	25.34698	0.468562
**BCL2**	1.046684	1.07707	0.910723	1.345478	1.316196	1.534098	0.467488
**BX679664.3**	43.97586	36.87049	38.92918	48.70486	64.53618	52.36288	0.467404
**TRIM71**	3.59791	3.184246	3.690938	4.77154	4.387642	5.31423	0.466717
**MND1**	8.932886	7.451355	7.616373	12.14684	9.83234	11.18564	0.466582
**QRSL1**	3.445019	3.336548	3.509348	5.38147	4.132012	4.698316	0.465718

**ENC1**	3.112153	2.988945	3.266725	4.802934	4.099187	4.034057	0.465626
**RPS27**	423.6052	439.2665	417.3309	607.7053	600.2772	558.9128	0.464844
**SOCS2**	2.37945	2.249161	2.245008	3.337315	2.954302	3.194081	0.464684
**RANBP9**	13.23624	12.72657	12.59431	19.10645	15.90459	18.18822	0.464409
**RRP15**	4.287638	4.387972	4.748848	7.37179	5.29523	5.845187	0.463613
**PPID**	11.52576	11.60252	11.96792	15.80421	14.79443	17.78579	0.463228
**TAF4**	3.902324	3.862841	4.230399	5.892769	5.13459	5.4972	0.462111
**HSPA4**	36.48453	38.03291	37.81255	60.86669	44.10897	49.68877	0.461398
**UBE2E1**	34.85042	32.83854	33.49309	53.80455	38.29903	47.20748	0.461356
**KARS**	30.32972	30.34078	30.42597	46.45763	38.45796	40.4681	0.460883
**ATP6V1E1**	23.63615	23.57742	22.88401	39.7535	25.93557	30.70285	0.459547
**PKNOX1**	3.154023	3.050225	3.199524	5.085782	3.751637	4.089296	0.459044
**OLMALINC**	2.15314	2.459514	2.23789	3.509153	2.937159	2.970234	0.45898
**DPH3**	10.50631	10.05948	9.903033	17.92367	11.68098	12.2703	0.458754
**NEDD4L**	1.953325	1.846797	1.907213	2.609157	2.491709	2.742757	0.458703
**RHOBTB1**	3.659012	4.784165	4.208032	6.235611	5.516648	5.624033	0.457845
**HSPA5**	218.8975	205.7897	197.5847	300.8174	266.0318	287.4555	0.457205
**RABEPK**	10.12294	9.918901	8.517868	12.6024	14.06838	12.53635	0.457135
**ZC3H15**	23.17	23.30227	23.46467	38.68932	27.37576	29.94242	0.457093
**RHOA**	218.0399	206.3728	199.5638	330.2856	254.6856	270.8104	0.455751
**NDC80**	8.176623	7.931719	8.549192	13.17669	9.375808	11.25917	0.455492
**EIF3I**	135.678	125.9479	127.6787	198.7135	159.1372	175.6763	0.454662
**CREB5**	3.085337	2.838345	3.0443	4.369726	3.890937	4.028917	0.45458
**GLUD1**	29.25589	29.69563	30.29767	43.99676	37.81802	40.47715	0.454418
**ARRDC3**	5.178957	6.099583	5.603257	8.665499	7.031917	7.432285	0.454278
**RNF19B**	13.53089	11.59639	13.05904	18.92027	15.15056	18.21099	0.453254
**TOMM22**	54.21214	47.46015	49.60743	79.13685	62.31753	65.63929	0.453065
**ABTB2**	4.045236	4.097627	3.697343	4.346359	5.767343	6.089893	0.45262
**MSN**	30.00614	31.76147	30.55734	40.92868	40.02067	45.36641	0.452242
**EIF3M**	17.54893	16.5407	17.51739	29.49508	19.50541	21.60063	0.452124
**CCNB1**	51.20489	50.30746	49.98844	80.98184	59.37767	66.85043	0.451768
**SMS**	70.22719	64.84903	66.83394	99.40948	82.80717	93.91491	0.451642
**DCAF13**	3.919471	3.825385	3.906512	6.27717	4.66456	4.988196	0.45124
**PDLIM1**	20.4305	18.77593	19.0434	27.41063	25.19849	26.86442	0.448221
**CENPW**	47.91081	45.07153	38.82425	68.86298	58.03892	52.81267	0.447285
**PALLD**	5.948781	6.027414	5.828819	8.64712	7.371231	8.253415	0.446996
**NCAPD2**	29.62025	31.96057	33.12262	45.09094	39.99239	44.01545	0.446987
**NUP37**	8.990769	8.753222	8.800973	14.72878	9.858583	11.58549	0.446469
**USP14**	12.18656	12.64203	13.13886	19.57956	15.08385	17.034	0.445329
**FKBP5**	5.299218	5.014894	5.111299	7.288756	6.42349	7.282188	0.444698
**NPM1P27**	27.57884	29.86376	31.44965	36.68619	43.37002	40.91078	0.444484
**TPMT**	7.71912	7.469186	7.402011	11.12637	8.917575	10.68664	0.443971
**PAQR5**	2.079514	1.884625	2.224499	2.746098	2.603341	3.06573	0.44337

**MRPL3**	42.44153	39.45131	39.85445	63.20471	48.81879	53.37923	0.442093
**SEC61B**	64.79217	59.35823	64.0649	83.52511	87.65618	84.5107	0.442023
**NUDT2**	14.06949	15.17262	14.10984	17.22013	19.33152	22.30293	0.441058
**GNB4**	4.94114	5.257673	5.421559	7.781957	6.57791	6.843284	0.44085
**CTNNAL1**	20.96422	20.14019	19.78634	32.71961	23.1947	26.70968	0.440338
**KLF9**	7.646045	8.85151	8.307425	10.21311	11.11046	12.33244	0.440234
**NPM1**	299.1801	299.2553	298.8044	459.3097	352.6481	405.0324	0.439752
**MRPL13**	10.99112	10.24223	10.47595	16.39295	12.73265	13.83643	0.438156
**RRP12**	8.697536	6.991928	6.245123	9.76079	10.0304	9.91722	0.437664
**TAF1D**	12.00742	12.67518	12.39534	17.98174	16.54638	15.65879	0.43675
**MRPL39**	28.7757	26.81419	28.83162	44.6071	35.11373	34.5073	0.436235
**LIMS1**	5.077512	6.181017	6.326596	9.271411	6.56715	7.951835	0.436024
**WWTR1**	2.052719	2.376348	2.243185	3.388056	2.640874	2.990735	0.4349
**PARN**	9.249145	10.36542	10.48498	13.93356	12.4041	14.29369	0.432852
**CMSS1**	5.334104	5.141935	5.069807	7.199197	6.760642	7.021916	0.432606
**EIF3H**	14.88614	16.47455	16.7834	24.84819	18.48665	21.58578	0.431318
**RBM3**	59.68688	55.50023	54.03226	84.12126	67.83756	76.21089	0.431213
**AP3M1**	8.500578	9.044238	9.121456	14.04591	10.12779	11.77819	0.431051
**IDS**	12.65954	13.04752	14.3118	19.91646	15.87273	18.16049	0.430935
**LIN28B**	10.6158	9.343708	9.70463	15.89438	11.81556	12.27053	0.430575
**GYG1**	6.867208	7.376809	7.334224	10.5	9.0382	9.540611	0.430391
**ASAP1**	5.33258	5.563216	6.113412	8.701466	7.171713	7.042752	0.430035
**STMP1**	9.106158	8.944051	9.301619	12.06474	11.80827	12.97633	0.430002
**ATP5MD**	63.67392	57.26516	57.88433	84.00721	75.92423	80.90544	0.429521
**C16orf87**	3.815721	4.020436	4.431245	6.083553	5.210256	5.209462	0.427922
**BZW1**	21.05808	21.50819	21.98936	32.72476	25.85907	28.24034	0.427554
**HPRT1**	86.5744	87.2014	91.58038	137.8541	100.3261	118.6953	0.427491
**TIMP3**	10.18689	10.8536	10.31684	14.29284	14.00254	13.87326	0.427367
**MYL12B**	32.90275	29.33762	31.08437	44.39653	37.49642	43.60239	0.427302
**SNRPD1**	21.82322	20.50235	20.38044	34.88137	22.59296	26.83485	0.427086
**AIFM2**	3.377503	3.067972	3.228639	4.485758	4.387899	4.132905	0.427038
**NUDT4**	4.439913	4.103427	4.497704	7.175143	4.832243	5.520667	0.426606
**TEAD1**	5.072612	5.934826	5.923999	8.293433	6.995279	7.466197	0.426473
**TBC1D12**	2.815822	3.476253	3.159985	4.095643	3.988408	4.613394	0.425838
**SRP54**	15.40584	16.65011	17.17185	23.98411	20.07886	22.05362	0.425539
**COBL**	0.891698	0.975596	1.038645	1.31209	1.295007	1.292478	0.424312
**REPS1**	6.473951	6.362184	6.644755	10.8444	7.025717	8.271375	0.424282
**UBE2V2**	8.637554	8.197774	8.757243	13.23902	10.15092	10.94601	0.423995
**GMEB1**	3.211892	3.042225	3.351879	4.445591	4.308437	4.132312	0.423835
**ACAP2**	4.305833	4.169918	4.43743	6.567243	5.371274	5.382732	0.423699
**FOXO3**	8.300197	9.098693	9.400012	11.38846	11.55676	12.99551	0.423446
**RAB18**	5.336296	6.101938	5.895161	8.772023	7.118864	7.349102	0.423055
**SPRYD7**	6.166974	5.511451	5.505056	8.48681	7.391109	7.155442	0.422703

**IRF2BP2**	32.65862	27.88442	28.53367	35.57944	37.17863	46.64083	0.422669
**GTPBP4**	17.08191	16.9603	17.05748	23.41617	21.93423	23.142	0.422629
**TOMM20**	120.4189	121.1778	124.5118	177.1011	148.8705	164.5386	0.422012
**RSL1D1**	17.09569	16.66718	19.51915	22.77729	20.52431	28.06361	0.421572
**KMT5B**	3.388664	3.104609	3.432654	5.068904	3.899808	4.323558	0.421314
**EIF2S1**	14.73037	13.38614	13.52214	20.36218	16.50493	18.88589	0.421126
**DRG1**	44.5736	43.80545	39.66265	68.11993	52.19092	51.12473	0.421053
**ARID1A**	9.456198	9.380115	9.738829	11.68811	12.46376	14.09943	0.420748
**UTP11**	11.29602	10.71195	11.2936	15.28758	13.67078	15.61178	0.420487
**CACYBP**	42.43935	39.27007	39.40144	61.72986	48.39652	51.9117	0.420005
**ACSL3**	10.33286	11.72541	11.06595	16.29996	13.68224	14.33422	0.419955
**TOMM70**	18.20497	18.6275	18.40015	28.89661	20.74654	24.24078	0.41974
**CCT6A**	73.56126	69.32325	69.23127	103.2924	85.074	95.23892	0.419033
**GNG4**	7.154932	7.799466	7.388335	10.19471	9.615072	10.03226	0.41754
**AFAP1**	1.853407	2.124284	2.069343	2.913509	2.486609	2.674726	0.417207
**CPNE3**	13.26225	14.61692	14.29027	23.17229	16.22352	16.88285	0.41639
**ANXA2**	15.39284	15.04944	15.42564	21.82504	18.57119	20.80507	0.416077
**HSPD1**	139.7127	142.7601	144.5573	196.8807	173.9857	198.8862	0.415998
**ARCN1**	36.74952	37.7059	40.1769	55.25903	42.49867	55.14719	0.415621
**OLA1**	14.55545	14.34482	14.33392	22.18258	15.88606	19.59576	0.415508
**FAM177A1**	2.747568	2.874414	3.092049	3.606342	3.96651	4.049606	0.415503
**COPS4**	10.60859	10.99659	10.94294	17.50538	12.53636	13.36102	0.415212
**SNU13**	33.47105	31.691	29.48078	45.35653	38.43496	42.38882	0.414922
**TBC1D9**	6.685782	6.899396	6.720621	8.963651	8.042785	10.05958	0.414591
**MAP3K9**	3.170712	2.775418	3.047297	3.659977	3.703507	4.621614	0.414299
**PRIM2**	5.063216	5.338622	5.149094	7.741429	6.280132	6.685829	0.413145
**PAK1**	5.588931	6.306964	6.552038	8.674534	8.016306	7.873563	0.41311
**PPA1**	25.50176	25.88793	26.86013	40.75182	29.75498	33.67607	0.412959
**FBXO3**	3.122744	3.33646	3.266429	5.074759	3.750567	4.123117	0.412915
**OSTC**	66.54974	64.57222	59.88175	96.00824	73.58564	84.6359	0.412532
**YWHAB**	20.07709	19.72395	21.83819	32.76841	22.01492	27.16667	0.410895
**PSMD12**	19.88245	18.65151	20.23818	32.19709	21.82289	24.10947	0.410734
**TMEM167B**	4.915535	5.465887	4.877828	6.66859	6.535389	7.080928	0.410723
**AKIRIN2**	39.1811	35.29298	36.99069	50.27477	46.11912	51.74369	0.41035
**SRP14**	50.20386	49.79184	48.1837	77.45532	59.99482	59.46544	0.410232
**GSPT1**	23.91828	22.41405	23.21797	36.40232	25.86243	30.10736	0.409401
**ERG28**	16.50253	18.63278	16.51194	27.46338	20.37091	20.73864	0.408948
**MAP3K20**	2.05547	2.177878	2.051444	2.942198	2.70951	2.687986	0.40813
**SELENOK**	33.93934	29.95703	29.61274	45.47264	41.15248	37.40856	0.407553
**PPP4R3A**	16.53828	15.59027	15.94577	23.69747	18.60811	21.46075	0.407528
**TIPRL**	24.41579	23.23965	23.88156	31.55393	30.81955	32.50288	0.407359
**PARD3**	3.259074	3.063013	3.445949	4.182644	4.148669	4.620581	0.407023
**TTC1**	27.78248	24.29869	24.11705	39.15748	29.57539	32.29965	0.40699

**HPCAL4**	3.979678	4.158476	4.052101	5.384051	4.87557	5.895323	0.406247
**UPP1**	13.66237	10.93396	11.19431	15.41962	15.78841	16.21614	0.40604
**COX7A2**	55.52727	52.12024	48.22269	82.3598	64.26706	59.84493	0.405599
**SGTB**	4.895886	5.332615	5.553848	7.786011	6.637362	6.479554	0.405393
**NUP107**	9.987186	10.19822	9.841487	16.13726	11.64184	11.98636	0.40526
**SKA2**	26.19591	24.73671	27.63532	39.59276	30.1129	34.32736	0.405029
**PLK1**	25.34197	25.60498	24.89972	34.6806	30.96632	34.73615	0.404358
**CCND2**	4.924082	4.713453	5.131994	6.194393	6.804435	6.539632	0.403693
**WDR12**	6.714714	6.62052	6.566977	10.11566	7.967461	8.242037	0.403514
**FOXO1**	3.797659	4.039186	3.976317	5.468267	4.486978	5.669535	0.403441
**MAPK6**	4.966904	5.33448	4.983614	7.952696	5.9513	6.309263	0.403186
**EMC2**	4.176232	4.642171	4.524777	6.342498	5.614975	5.673605	0.402018
**ARRDC4**	16.68264	14.94981	16.03686	22.60125	20.28166	20.04901	0.400731
**NDUFAB1**	51.47112	47.59923	48.227	70.01028	55.82471	68.51894	0.399955
**POMP**	57.56714	51.26393	53.57269	70.45129	71.41238	72.32681	0.399309
**CCNB2**	22.774	23.43004	23.07773	35.10999	25.95944	30.27968	0.398915
**CENPL**	4.033602	4.512722	4.594169	6.389125	5.169811	5.766151	0.398843
**PNP**	20.04891	19.51733	18.34467	27.47024	21.6342	27.22624	0.398428
**RNF217**	2.260048	2.385213	2.525727	3.341653	3.00305	3.103022	0.397795
**TPX2**	49.73026	51.28872	51.77441	73.15902	59.42641	68.70234	0.397677
**PDK1**	9.332511	9.07513	9.41533	14.85506	10.40452	11.38708	0.397405
**SNRPB2**	26.42623	26.38875	23.39124	36.0282	29.28577	34.99712	0.3965
**PPP2R5C**	6.70029	6.773961	7.112811	10.63281	7.626762	8.833781	0.396201
**KRT18**	14.06562	13.80878	11.9071	18.31984	15.94239	18.08554	0.396031
**AURKA**	19.94226	21.50728	20.88707	28.77356	23.94436	29.27976	0.395503
**ENOPH1**	33.93002	33.5714	32.11731	46.20118	39.98572	44.69677	0.393796
**TIMM10**	25.27184	24.35259	23.38021	31.87881	31.82907	32.18545	0.393442
**TCP1**	74.83719	73.13028	75.36735	107.1429	88.99435	97.10562	0.392888
**EIF3D**	65.90928	64.62503	65.07818	92.76235	78.10069	85.94778	0.392708
**ARF1**	188.6742	184.4569	175.5264	254.9832	220.5358	244.7812	0.392692
**MRPS9**	15.27464	16.78862	15.65713	23.06815	17.9068	21.66526	0.392483
**XRCC5**	54.97202	55.61444	56.40999	84.76183	62.06391	72.35461	0.392301
**EIF4E**	3.612008	3.250227	3.350799	5.009628	3.985238	4.409124	0.392251
**CAPN2**	17.76641	17.77739	18.34043	24.05186	22.53399	24.12969	0.392164
**CETN3**	8.119819	9.289715	9.554012	11.86913	10.46238	13.0499	0.391981
**DNAJA1**	79.13385	76.80688	77.12282	120.2632	81.778	103.7627	0.391884
**DUSP16**	5.271739	5.437243	5.754308	6.927458	7.041579	7.629031	0.39165
**PA2G4**	47.32817	44.7163	44.82524	60.05917	54.96095	64.38608	0.390427
**VBP1**	64.11892	56.4717	59.27899	93.19198	65.39123	76.98898	0.389218
**C12orf75**	16.80771	14.0845	14.40849	19.04015	18.20035	22.07989	0.388995
**ALYREF**	85.28356	84.20545	81.99552	103.576	112.2105	113.5256	0.388986
**CLIC1**	63.23872	56.83	53.62938	70.5441	74.21376	82.67983	0.388889
**GNAQ**	7.006563	7.433122	7.616047	11.40016	8.531313	8.946779	0.38883

**SNRPE**	81.78686	71.27234	73.94744	101.2195	99.42122	96.53062	0.38856
**CKAP5**	23.87352	28.47473	29.06498	39.43317	32.54603	34.56619	0.388133
**MTFR1**	10.72883	9.923309	9.935032	14.98511	12.18825	12.84536	0.387748
**HBS1L**	10.05872	9.550162	9.522617	13.59879	11.92571	12.58889	0.387718
**OGFOD1**	8.67488	8.545754	8.794349	11.22268	10.48277	12.32252	0.387378
**CRABP2**	115.4439	99.31891	99.0094	131.7137	134.2037	144.2989	0.386667
**PNPT1**	7.822352	8.788782	8.786015	12.87602	10.08889	10.23373	0.386457
**NCL**	109.4234	118.8779	118.4326	134.9698	149.804	168.3021	0.385924
**UGP2**	5.113791	4.734702	4.44256	6.965065	5.475029	6.229005	0.38554
**MAPRE2**	10.48955	10.82716	11.66545	15.42807	13.38537	14.26835	0.385392
**CCT8**	91.0823	88.0415	89.57226	130.8215	102.3564	117.6689	0.384866
**ELMSAN1**	4.434602	4.743248	4.440718	5.031218	6.034933	6.715307	0.384799
**NUP88**	8.507986	8.110749	8.353898	11.30474	10.07224	11.21735	0.384273
**CTNNA1**	20.7681	21.16253	21.18274	29.52299	23.82471	28.97132	0.38328
**TMEM14B**	15.23733	14.35887	13.54391	22.05171	17.06811	17.12204	0.382614
**ZNF326**	12.22444	12.01592	12.50861	17.66242	14.80067	15.41871	0.381774
**ZBTB43**	3.571069	3.618078	3.69528	4.765305	4.26438	5.151269	0.381689
**UBE2E3**	11.51806	11.20285	12.0133	16.45092	13.91968	14.88132	0.381622
**ESD**	15.87485	15.48255	16.33564	23.72034	17.79003	20.62033	0.381528
**PDP1**	5.293186	4.676003	5.256879	6.992586	6.096175	6.742784	0.381254
**CDC20**	56.83044	54.99326	53.33163	70.21446	69.06615	75.82066	0.381193
**MYL12A**	11.27055	10.69917	11.16939	16.56298	12.82208	13.77535	0.381174
**EIF5**	27.17932	27.51308	29.02223	42.69643	30.24999	36.08059	0.381134
**THAP9-AS1**	16.64381	17.74901	18.54183	23.56199	22.63094	22.74145	0.381011
**BUB1**	16.62002	19.19063	19.48817	27.57656	21.19944	23.22087	0.380686
**PTGES3**	120.0018	119.0717	120.3841	171.7255	139.38	156.791	0.380368
**RYBP**	7.298558	7.410885	7.394711	11.10109	8.186776	9.481497	0.380216
**ZPR1**	7.951499	7.479162	6.95487	10.91376	8.482851	9.736827	0.380109
**CKS2**	99.47721	98.88872	100.9182	108.783	137.801	142.9116	0.380092
**LEF1**	4.745763	5.014848	4.918442	6.709845	5.97786	6.412344	0.379817
**FAM98B**	6.185774	6.729855	6.744961	9.595293	7.999357	7.98709	0.379808
**UBXN2A**	4.615746	5.001328	5.337065	7.305424	5.928469	6.223093	0.379743
**LRP8**	3.693286	3.345369	3.217953	4.760401	4.264063	4.318292	0.379503
**COX17**	15.54441	13.81663	13.78422	18.83744	17.99914	19.27779	0.379168
**YY1**	18.9521	18.26784	18.2542	25.34205	21.57221	25.2235	0.378939
**FAF1**	7.258706	6.831423	7.106348	9.974304	8.310565	9.269523	0.378458
**NENF**	64.69309	56.07863	54.39415	83.00038	75.82209	68.8709	0.378371
**PES1**	41.32119	36.89146	36.70084	45.05396	49.6612	54.59312	0.377746
**SNRPG**	50.49429	48.70071	49.5957	62.96137	68.68906	61.65056	0.377565
**ELK4**	3.408029	3.769046	3.802144	5.327422	4.328725	4.597964	0.376603
**WDR3**	7.187199	7.928681	8.003301	10.62034	9.291354	10.0965	0.376266
**CTNNBL1**	10.707	10.18644	9.226843	12.58962	13.3419	13.15492	0.375933
**JMJD6**	8.772534	7.93227	7.785942	10.99551	9.700218	11.06994	0.375231

**CCT5**	49.95648	47.72212	47.46878	67.73007	56.20709	64.25723	0.374705
**PDE8A**	5.617764	5.472021	5.831761	7.93583	7.029162	6.972765	0.374555
**ACP1**	28.39259	26.86937	26.56195	36.92669	32.73525	36.36352	0.373816
**SLC16A1-AS1**	4.840409	5.105261	5.367042	6.06926	6.618846	7.151365	0.373644
**PDCD5**	38.34203	35.32337	37.55657	44.65355	51.73734	47.70925	0.37363
**AP2B1**	16.98132	19.4893	20.28859	25.34878	22.49238	25.67894	0.373284
**PTBP3**	9.313373	10.0179	10.00246	14.84057	11.0045	12.13669	0.372746
**GTF3C6**	51.7092	47.6646	46.73802	56.82862	66.46393	65.76193	0.371729
**SOGA1**	4.011349	4.332491	4.057232	4.915527	5.450308	5.674633	0.371251
**CS**	27.11383	28.5466	29.23709	40.42406	32.97256	36.39902	0.371026
**ABLIM1**	5.02812	4.963711	5.332126	6.783932	6.125245	6.905741	0.370798
**PGBD5**	2.420823	2.204118	2.110266	2.796047	2.799306	3.112136	0.370535
**VDAC2**	49.07953	45.82338	45.21625	71.02517	51.71128	58.36573	0.37015
**SNRPC**	95.82289	84.68108	82.52379	111.6454	107.237	121.0343	0.369966
**TRAPPC1**	50.93788	49.4635	50.90869	72.4402	60.5994	62.46849	0.36972
**DPH5**	11.91568	11.02679	10.91197	15.03327	13.87329	14.83197	0.36956
**XRCC6**	185.1459	189.103	176.6144	256.1997	214.1691	241.2945	0.3695
**TPM3**	42.14602	38.52838	36.38784	53.12274	47.15274	50.90134	0.368961
**BEND4**	5.121889	5.216309	5.23686	7.613983	6.119407	6.373841	0.368477
**MCUB**	8.503465	7.755238	7.826985	11.29665	9.339335	10.45505	0.368322
**FTL**	584.3888	561.3555	516.1623	747.8185	635.5867	761.3766	0.367992
**MAP3K21**	11.97309	12.21671	12.77791	15.2136	15.48493	16.98907	0.367348
**TSN**	20.39044	18.93271	20.16063	27.0527	22.45737	27.17576	0.366464
**SMNDC1**	9.065727	9.301684	9.849148	13.92171	10.87097	11.56972	0.365905
**ORC1**	13.01376	13.66879	14.10649	19.8399	15.53957	17.17531	0.36564
**AIMP1**	11.58949	12.73491	12.07	17.61951	14.60624	14.66123	0.365471
**NBDY**	23.61328	22.01937	23.27359	31.57858	26.37308	30.77912	0.3648
**RNASEH2B**	1.931198	1.828591	1.811293	2.582387	2.277415	2.314014	0.364783
**ZNHIT6**	6.934067	7.246661	7.044105	9.326493	8.401752	9.58281	0.363732
**DNAJC7**	9.835741	9.911174	9.836658	13.66226	10.98999	13.40985	0.363559
**B2M**	29.9962	29.44367	26.77945	42.59789	32.79004	35.50241	0.36305
**ZCCHC14**	9.327492	10.51999	11.06806	14.31099	11.95292	13.48961	0.36275
**CREB3L2**	10.25484	11.53548	11.50499	14.11692	13.05973	15.634	0.362651
**CSNK2A1**	5.927174	5.691453	6.065193	7.640077	6.981275	8.115828	0.362623
**MELK**	24.43751	25.68315	25.18186	36.048	29.44298	31.32673	0.362573
**USB1**	7.288558	6.909664	6.946088	8.583636	8.846502	9.748204	0.362188
**PRDX6**	79.53682	83.12386	80.39987	105.8463	98.88933	107.624	0.361892
**NAA20**	20.12406	18.08969	18.89181	22.665	25.33236	25.36371	0.361384
**PSMA5**	15.99611	15.30206	15.27549	21.91245	18.8821	19.01913	0.360961
**PITPNB**	19.44517	20.09869	20.40033	30.23739	22.27145	24.45367	0.360536
**SET**	127.9217	118.0393	120.1402	172.2364	143.8871	153.8767	0.360419
**TCEANC2**	5.360963	5.551774	5.888522	7.209067	6.827797	7.520148	0.359588
**PUS7**	9.535594	9.654251	10.30272	14.34594	11.63476	11.84958	0.35919

**COPZ1**	39.14104	37.31186	35.83071	54.36939	43.96372	45.6296	0.358548
**HDDC2**	13.55336	12.01119	12.76817	17.86341	15.90919	15.35262	0.357887
**VDAC1**	70.17141	71.85508	69.8271	90.29897	86.22078	94.9741	0.357852
**GTF2A2**	12.03802	11.54424	10.58293	13.62347	15.84395	14.2858	0.356862
**RAB11A**	21.45109	22.11316	22.52776	31.006	25.94941	27.67291	0.356665
**SYAP1**	14.56602	14.44783	14.64737	19.60854	17.29489	19.00018	0.356589
**EHD4**	5.542966	5.12371	4.6897	6.67469	6.226478	6.760564	0.356553
**LARS2**	10.74897	10.53164	9.652865	14.04656	11.8438	13.70747	0.356252
**ELOC**	10.86757	10.29033	9.58005	13.69639	12.83575	12.81081	0.356084
**NUP155**	16.50129	18.75975	18.39089	25.75668	20.47085	22.4015	0.355189
**CIAO2A**	15.34345	15.3283	13.98921	20.95361	17.75394	18.41822	0.355128
**TMEM68**	3.01479	3.050715	3.02389	3.907133	3.847665	3.871143	0.355091
**STRAP**	67.36962	64.59238	66.80796	94.26219	75.09118	84.79614	0.354578
**SIPA1L2**	4.312121	4.627792	5.003121	5.829555	5.756642	6.240497	0.354495
**HIP1**	5.109337	5.446269	5.61939	6.995929	6.43208	7.239424	0.353594
**COX6C**	37.01589	37.9229	35.57949	52.41836	43.34706	45.43494	0.353459
**LARP4**	9.951515	11.14577	11.73537	15.55265	12.39406	13.9954	0.353268
**ILF2**	135.0789	129.3868	127.2731	185.8514	151.9884	162.1749	0.352079
**MED10**	16.87691	16.41804	16.51152	21.23177	21.89298	20.3804	0.350541
**MAPK1IP1L**	12.28247	12.84051	14.0899	18.04673	14.61883	17.31904	0.350156
**DYRK1A**	3.243126	3.379473	3.487415	4.499458	4.025775	4.355432	0.349422
**CPSF2**	5.905328	5.775161	5.915858	8.56834	6.57333	7.273647	0.349209
**TFDP2**	7.658904	8.413002	8.061093	10.96709	9.52992	10.24027	0.348982
**RPL37**	51.69357	49.4469	48.77678	57.14384	66.74548	67.03143	0.348808
**RRM2**	20.50105	19.45465	20.30294	28.12763	22.67398	25.93305	0.34871
**KRAS**	7.773739	7.996805	8.834351	11.39375	9.675118	10.25878	0.348491
**MRPL21**	22.77688	19.56373	20.07219	27.23504	26.59686	25.6233	0.3483
**PSMC2**	22.17531	21.47132	20.02657	30.66137	24.59962	25.78712	0.348092
**AGPS**	10.39062	10.61055	10.63353	15.12289	11.82559	13.30783	0.347707
**RMND5A**	14.82123	13.8484	14.60702	20.39752	16.43906	18.22014	0.347329
**RPSA**	393.8395	368.5935	356.4765	546.6221	433.2875	441.5421	0.345272
**PSMD10**	43.50642	42.74024	42.71254	60.80237	49.84901	53.15161	0.345047
**RACGAP1**	18.33738	19.77822	19.82084	26.44145	21.85691	25.21691	0.343573
**EIF3J**	21.07669	19.48218	21.4601	28.01502	24.10681	26.55763	0.343278
**RPL38**	91.79735	90.33789	92.25152	103.2844	129.19	115.4451	0.342543
**MCCC2**	11.84037	12.47971	12.34962	16.84724	13.47121	16.15987	0.341969
**GNAI3**	3.962791	3.788833	3.97219	5.596892	4.329192	4.929348	0.341548
**HOMER1**	5.299983	4.994717	5.079967	6.434868	6.476626	6.561594	0.340927
**EZR**	43.60772	39.85084	38.80259	49.72592	49.71911	55.3557	0.340446
**ATP5PF**	43.80366	37.13123	37.74797	54.18585	48.90465	47.16666	0.340322
**IFT57**	13.05975	12.64062	13.00786	17.77666	16.17309	15.04772	0.340067
**TBC1D4**	8.191442	8.989162	9.026487	11.37898	10.56694	11.22186	0.339825
**ST13**	88.94925	91.51424	93.59848	120.2453	104.8777	121.7239	0.339797

**CCT2**	55.8118	53.69559	52.48954	72.3993	62.94379	69.55866	0.338966
**FAM98A**	9.761559	9.517172	10.22361	13.0593	11.50152	12.75141	0.338819
**MAP4K4**	9.14831	9.41309	10.07867	11.47559	12.09445	12.65032	0.338766
**ATP5PB**	41.43957	40.81048	41.94268	53.80242	48.34702	54.91088	0.338738
**PSMD14**	8.497934	8.13599	8.253881	11.69575	9.317346	10.45628	0.338509
**RDH10**	9.076394	8.335093	8.758987	11.55376	10.86466	10.67176	0.338463
**PSMD11**	17.94543	17.2607	17.85777	23.43902	20.86491	22.76264	0.337863
**RAB2A**	13.7284	13.30332	12.7663	16.99358	15.77377	17.52324	0.337592
**RPS13**	191.7667	170.0841	167.3769	254.433	202.1431	212.027	0.337262
**SRP9**	226.3081	209.7634	217.3787	310.5481	240.2395	274.5529	0.336912
**PMAIP1**	103.9072	87.07548	88.54162	122.545	112.8029	117.5733	0.336372
**RPL26L1**	45.01739	43.03054	43.63868	51.23447	56.06667	58.93488	0.336124
**FKBP4**	54.03463	48.80161	47.32789	55.15846	65.85325	68.44576	0.335334
**SETD7**	8.710798	7.93687	8.849702	11.73031	9.953478	10.46645	0.334481
**CBFB**	19.80192	17.75018	19.15798	24.96395	22.07793	24.45831	0.334342
**PSMB3**	80.95387	75.48917	74.57332	107.6777	89.92065	93.50477	0.333535
**ARFGEF3**	2.940579	3.421452	3.278022	4.015736	3.99755	4.127937	0.332801
**ANXA5**	63.23358	60.74374	59.22215	80.91284	70.71405	79.06106	0.332527
**PRPS1**	73.63514	67.58509	65.61036	85.69163	85.79844	88.89801	0.332214
**CCT4**	106.9537	105.2851	105.3096	132.0322	126.6591	141.0507	0.332093
**HTATIP2**	12.41471	12.39757	12.12289	14.66575	15.87074	15.94076	0.33153
**TMSB4X**	58.06139	54.15889	55.67905	67.73453	66.07584	77.46417	0.331523
**HMGB3**	32.39336	34.10114	33.1672	40.12845	40.82232	44.43668	0.331282
**LRRC59**	41.52732	38.80992	37.41655	48.95347	47.09402	52.04477	0.330723
**COPA**	27.5229	28.79121	29.1519	36.83266	32.70275	37.78812	0.328544
**TBCB**	21.93655	22.07424	20.86674	27.24365	27.79959	26.40837	0.328224
**TCOF1**	32.05295	29.18736	28.56675	33.87379	38.38247	40.48507	0.328116
**FUBP1**	35.69396	38.45031	38.94152	46.95591	45.26443	49.73188	0.327988
**LAS1L**	19.45255	18.40618	18.94219	22.83694	24.75898	23.69307	0.327765
**JPT1**	36.22148	33.76889	33.67051	41.17538	44.93678	43.95096	0.32734
**SSBP1**	29.03959	25.03537	26.45772	34.50816	32.1604	34.35962	0.327111
**RSL24D1**	30.15892	28.09887	29.07312	39.84876	33.6967	35.83685	0.324815
**UTP18**	21.18756	21.30267	20.35323	27.05837	24.7762	26.86967	0.324679
**UQCRQ**	80.667	70.84569	70.03399	93.78291	93.97627	89.54398	0.323853
**PRMT3**	8.73254	8.637149	8.58153	11.55304	10.20506	10.7155	0.323465
**BCCIP**	16.10566	17.21722	16.20412	20.64778	19.33872	21.98427	0.323373
**ZIC2**	29.56142	28.7253	29.33334	33.81005	35.01238	40.80333	0.323254
**USP39**	20.40808	20.12671	19.61436	27.61235	23.15761	24.47005	0.322956
**PSMB1**	77.5328	69.50912	70.55702	93.75731	81.21332	97.00354	0.321798
**UBE2R2**	19.44724	18.53169	19.08724	23.2557	22.54673	25.51435	0.321606
**STOM**	15.56431	16.63936	17.08303	21.01622	19.38245	21.18791	0.321417
**CCT3**	146.8788	141.6093	137.657	190.7136	159.8762	181.648	0.320726
**UBE2H**	8.802795	9.005676	9.146824	10.61017	11.35391	11.70152	0.320706

**LAP3**	13.01373	13.44807	13.08236	16.08959	16.87456	16.36636	0.319015
**NUP93**	9.623489	10.02972	9.526614	13.20414	11.10416	12.05947	0.317689
**SNHG3**	18.41748	17.67688	16.78305	23.10456	21.25631	21.53264	0.317485
**PLEKHB2**	13.90586	13.38763	13.71033	17.97029	15.38391	17.73825	0.317352
**MAPRE1**	74.17088	68.3685	70.13231	95.53595	79.41575	89.90391	0.316578
**SF3A3**	20.59629	21.38687	22.29779	26.72764	24.33594	28.96136	0.316058
**CCNY**	9.471903	9.079301	9.290064	11.96848	11.10577	11.58546	0.316035
**NAMPT**	12.66848	12.75449	12.65446	16.34244	15.12839	15.89299	0.314849
**SSB**	36.06118	35.37487	37.40984	49.26766	41.80295	44.22166	0.313792
**AP2S1**	72.54879	64.76752	63.0606	79.62552	87.16067	82.22214	0.313478
**GFPT1**	11.47013	11.0284	12.23431	15.46971	13.90164	13.7454	0.311948
**PHB**	36.94116	33.77496	32.59316	43.27224	45.083	39.76305	0.310506
**MTCH2**	33.82679	31.99288	31.44517	40.69952	37.57133	42.3305	0.310256
**ENAH**	15.97767	17.18485	17.29555	20.32422	20.37749	21.8111	0.309067
**CAPNS1**	34.93827	34.19374	31.49156	43.88034	39.23172	41.53702	0.308904
**H3F3B**	80.01216	75.96764	73.70201	101.6903	90.7123	92.09949	0.308803
**ELF4**	24.72739	23.24985	23.63014	30.07225	27.19466	31.41667	0.308559
**CNN3**	38.80393	36.1576	37.27263	46.60449	44.40972	47.98025	0.308516
**RPS28**	71.16717	66.38662	62.15505	76.71322	85.38375	85.10317	0.307781
**AAGAB**	17.7518	17.99288	18.46326	22.92661	21.04276	22.87537	0.30231
**SMU1**	11.59964	11.72733	11.96127	14.24402	13.8144	15.43614	0.301647
**SUMO1**	62.15119	60.19891	59.17135	79.05494	71.12194	73.50874	0.301332
**GLUL**	25.72857	28.24921	26.70395	34.65622	32.54501	32.20991	0.301165
**ACLY**	77.67368	75.40348	71.95052	90.53282	89.82	96.23072	0.297613
**AK2**	60.08959	55.28581	55.91921	70.55143	69.80678	70.00744	0.296419
**GAPDH**	680.5704	647.3037	612.809	821.0443	738.579	822.741	0.295829
**RAD23B**	47.0119	45.62985	48.47869	53.98012	56.876	62.36675	0.295702
**EPB41L2**	15.47629	15.4688	16.15471	18.94153	18.42339	20.43472	0.29534
**SNRPD2**	107.4665	99.38387	95.89186	125.5551	122.0079	123.6337	0.294094
**NAA50**	36.67518	34.71484	35.4541	43.53619	41.48789	44.25135	0.27494
**TBC1D9B**	30.18199	29.29863	28.28994	23.67989	24.44707	25.29692	-0.25749
**DAG1**	46.5518	48.58547	42.65384	38.24028	37.97943	38.48724	-0.26453
**QARS**	50.10483	48.55666	44.81441	38.81687	38.94475	41.13014	-0.27116
**TIMELESS**	35.21271	34.61287	34.18023	28.86206	27.75753	29.44471	-0.27318
**PHGDH**	72.57522	64.81165	60.48291	52.52712	53.99056	55.67572	-0.28684
**PLEKHG4**	15.82537	14.97294	14.77798	11.80545	12.6788	12.8732	-0.28689
**HMGB1**	60.93835	58.79172	61.13337	49.8649	48.7965	49.14031	-0.29124
**GBA2**	19.34062	18.27196	18.20465	15.0992	14.75581	15.73972	-0.29184
**ARPIN**	9.552588	10.01753	9.968954	7.685931	8.471277	7.933392	-0.29415
**ANKRD52**	22.51066	21.85148	21.63669	15.89622	18.87477	18.94159	-0.29718
**KAT2A**	34.67399	32.98484	32.03166	27.37632	26.65185	26.7336	-0.30378
**ARL10**	21.40234	22.02323	23.79166	17.46265	18.41744	18.53703	-0.30477
**POLE**	26.53971	24.37036	25.02662	21.36335	19.97885	20.0299	-0.30721

**SLC19A1**	10.81133	9.360111	8.93067	7.563938	8.031387	7.920815	-0.30747
**TSC2**	10.03724	9.152667	9.139476	7.242966	8.180512	7.462252	-0.30785
**SPG7**	8.173779	7.058493	7.423164	6.005599	6.633905	5.624085	-0.31089
**GGA3**	14.8977	13.97123	14.28382	11.00111	11.82942	11.92079	-0.31239
**LUC7L2**	16.42306	17.61603	17.3762	12.60625	14.90928	13.81581	-0.31496
**FAM57A**	27.32989	25.68593	24.09037	22.51384	19.24778	20.22184	-0.31496
**UNK**	13.52495	12.1557	12.62293	10.27271	9.637017	10.87145	-0.31543
**PPP1R3E**	11.28454	11.15001	12.17571	8.711265	9.789089	9.311546	-0.3155
**CLTCL1**	4.599923	4.830968	4.791505	3.836042	3.783153	3.809028	-0.31556
**CREBZF**	40.07734	40.9096	39.85519	32.47451	31.62058	32.97547	-0.31602
**NUP210**	44.91619	43.48483	40.2778	34.0658	35.2348	33.97177	-0.31732
**CTBP1**	19.12011	17.31135	15.98453	12.86285	15.02582	14.164	-0.31781
**PIP5K1C**	9.04975	8.423026	8.865511	6.706853	7.213185	7.199614	-0.31858
**RETREG2**	12.95492	12.09272	11.73443	9.891224	9.262443	10.33769	-0.31871
**ZNF629**	15.17583	14.56534	14.58909	12.72628	10.81733	11.99867	-0.31876
**CENPT**	16.13648	13.97369	14.45628	11.08954	12.50339	12.1157	-0.31968
**MFSD12**	18.92083	16.47657	15.65866	13.18484	13.94923	13.65201	-0.32401
**FRMD8**	14.00185	12.82936	12.26549	10.41257	10.36802	10.42193	-0.32538
**CUL9**	5.659696	4.731416	5.092805	3.987919	4.275557	4.091985	-0.32562
**PROSER3**	7.498808	6.536169	7.136319	5.618897	5.682351	5.584407	-0.32631
**E2F1**	63.82434	52.02862	53.60598	40.8869	48.14784	46.07586	-0.32679
**NAA40**	18.22923	16.77478	16.73074	13.5601	13.4796	14.19308	-0.32734
**PHKG2**	10.56352	9.167436	9.823946	8.579385	7.645076	7.307565	-0.32877
**TEX264**	24.06151	23.15105	20.97685	16.13385	19.86173	18.27228	-0.32945
**PPP1R26**	15.40587	14.75951	14.54175	10.6655	11.95267	12.95883	-0.32956
**LAMP2**	48.40249	39.95391	43.51155	33.78095	35.7911	35.30014	-0.33046
**ERBB2**	12.64784	12.19051	11.76561	8.468596	9.997941	10.62577	-0.33136
**SLC26A2**	9.100977	10.06326	9.38634	8.475734	7.119093	7.095884	-0.33142
**FZD7**	13.78953	13.32331	12.88862	10.50707	10.66566	10.6125	-0.3317
**TMCO6**	11.70003	11.02642	11.97392	8.845071	9.228045	9.455939	-0.334
**PLXNB1**	5.725718	4.974619	5.345735	3.863327	4.297962	4.561298	-0.33483
**CYB5R1**	13.79349	13.09769	11.66944	9.235923	10.64305	10.68883	-0.33512
**CC2D1A**	18.95586	18.10435	17.68455	12.70131	15.11579	15.54893	-0.33616
**WBP1L**	5.57042	6.133547	5.489274	4.972013	4.169445	4.469267	-0.3371
**ORAI2**	7.05089	6.331065	5.853684	4.722337	5.210818	5.286533	-0.33784
**CGN**	9.407019	9.861283	8.537403	6.708866	7.849305	7.43851	-0.3381
**CLK2**	41.22179	38.8905	39.28745	30.7658	31.90813	31.77874	-0.33814
**C1QTNF6**	7.936425	9.792991	9.469278	6.64828	7.508974	7.339386	-0.33943
**ATM**	4.263803	4.586007	4.786027	4.152872	3.443268	3.180708	-0.33947
**MLST8**	19.30677	18.56778	18.75723	13.85609	15.11714	15.75366	-0.34047
**CCDC130**	14.27973	12.94523	13.53331	10.01863	11.45517	10.69573	-0.3414
**FTX**	1.565539	1.465217	1.538551	1.314424	1.178551	1.111441	-0.34221
**SFI1**	8.951797	7.943774	8.098613	5.767003	7.336909	6.592985	-0.34362

**STK36**	11.2405	10.55995	9.920641	8.607542	8.131697	8.254061	-0.3439
**CYB561D1**	6.388585	6.583241	6.106412	4.575042	5.20625	5.248578	-0.3441
**MEN1**	32.99955	31.40668	28.58896	21.65029	26.31106	25.28832	-0.34433
**H2AFX**	116.8504	104.8762	99.27812	77.12531	83.34443	92.37461	-0.34434
**MRI1**	21.62499	20.61216	19.01011	14.68439	17.2082	16.3311	-0.3449
**SLC12A6**	2.908805	3.175832	3.15265	2.583473	2.379005	2.308162	-0.34539
**SLC38A7**	6.336959	6.129693	5.361074	4.94229	4.462096	4.621898	-0.34599
**SLC7A5**	249.763	201.2019	178.7333	157.9913	166.5965	170.692	-0.34642
**COL4A5**	3.188389	3.294178	3.47469	2.74806	2.519416	2.558914	-0.3474
**TMEM214**	24.01798	21.92807	20.52896	17.31932	16.83843	18.04461	-0.3487
**PABPC1L**	24.45528	21.22456	21.967	15.44775	19.61432	18.01453	-0.34995
**SGSM2**	11.77779	10.62908	10.34754	7.486698	9.449061	8.74273	-0.35113
**PIGO**	12.04444	12.31029	11.0279	9.425181	9.51353	8.794526	-0.35143
**TMEM198B**	7.282729	6.83667	7.998639	5.586999	5.906624	5.827096	-0.35272
**SETD6**	8.775215	8.700235	9.421264	6.76274	7.168388	7.13116	-0.35277
**TRIM45**	8.491447	8.330676	8.206917	6.007864	6.915535	6.670759	-0.35318
**TMEM39B**	18.60787	16.34973	15.57336	14.73263	12.17603	12.61063	-0.35461
**AP5Z1**	6.0473	5.231412	5.335725	4.165349	4.479865	4.335782	-0.35604
**THOP1**	31.21277	29.14215	27.07978	19.00253	25.59313	23.69938	-0.35643
**NLRX1**	12.50498	12.04631	11.66979	8.234468	10.00614	10.04844	-0.35659
**FZD6**	16.2838	16.24985	16.58514	15.24007	11.18516	11.92783	-0.35693
**CBX2**	24.63796	24.24663	22.92663	17.30186	18.83131	19.89595	-0.35803
**POLL**	10.79371	9.422912	9.713532	7.199574	7.776061	8.374413	-0.35817
**FURIN**	35.36849	33.75533	30.23484	22.42411	27.75833	27.24749	-0.35975
**GOLGA8A**	31.78063	29.56101	30.86528	26.96497	24.79602	20.04273	-0.36082
**LAMA5**	9.09549	8.097061	7.744319	5.332219	7.038378	7.047841	-0.36085
**CELSR2**	14.19716	14.7264	12.21143	9.845964	10.99843	11.18456	-0.36099
**CERCAM**	24.21166	23.58465	22.42869	14.66858	20.79385	19.20454	-0.36132
**TMEM63B**	16.66385	15.85658	14.02016	11.65891	13.07474	11.48457	-0.36177
**ZDHHC8**	19.30391	18.84809	17.65481	12.30766	15.36364	15.70394	-0.36357
**LZTS2**	17.40254	15.96788	15.68553	10.45853	14.21184	13.45684	-0.36361
**PKN3**	30.52606	28.8824	29.04276	19.19725	25.05535	24.47276	-0.36404
**DYRK1B**	7.171189	7.000863	7.279807	5.354546	5.568339	5.738198	-0.36462
**AC018638.4**	25.17835	27.24942	28.48808	22.54505	19.47583	20.81514	-0.36483
**PLD3**	44.64101	45.31207	38.25838	29.76441	35.67185	34.10796	-0.36512
**RNF207**	4.773386	4.342748	4.419369	3.346266	3.837479	3.321568	-0.36563
**TXNRD2**	6.995802	6.815426	6.20876	4.346375	5.891284	5.288474	-0.36674
**ID3**	26.07306	33.47127	33.24783	23.47083	24.07811	24.38594	-0.36731
**NOTCH3**	16.05314	14.73783	13.21765	9.472225	12.86228	11.74352	-0.36894
**JRK**	7.870824	7.685877	7.327279	5.502993	6.09568	6.119415	-0.36912
**CSPG5**	8.732495	9.158136	7.422937	6.938106	5.839262	6.805189	-0.37034
**PLXNA3**	16.56981	13.4937	12.4523	10.38325	11.11564	11.3867	-0.37054
**THAP7**	29.52981	26.1112	25.08675	19.58192	20.71938	22.06129	-0.37238

**KIF1A**	15.31019	13.32376	11.49673	9.024858	11.14644	10.79249	-0.37412
**ZNF276**	8.509265	7.130176	7.682331	6.061255	6.224682	5.704653	-0.37443
**BRF1**	6.529795	6.255502	6.007693	4.257676	5.34506	4.893459	-0.37452
**POMT1**	20.00451	19.10109	18.38006	13.62639	15.49159	15.17698	-0.37606
**GPR153**	6.133086	6.166636	5.941031	4.187092	4.882063	4.983581	-0.37631
**TSPYL4**	21.06428	21.45615	21.50865	14.98434	16.52554	17.80203	-0.37679
**NAGLU**	24.57107	25.52346	23.76397	16.19362	20.35029	20.31589	-0.37736
**ZNF891**	2.306595	2.559452	2.69324	2.123331	2.021313	1.672556	-0.37793
**NOP9**	9.047247	8.678349	8.543844	6.241398	7.090109	6.883343	-0.37797
**RRNAD1**	17.3171	17.92638	16.51584	11.33446	14.12103	14.37118	-0.37808
**GOLGA8B**	17.00006	13.49041	17.52233	12.81493	11.90865	12.19949	-0.3789
**OBSL1**	24.26709	22.68108	22.40677	15.23361	19.76847	18.32958	-0.37901
**WSCD1**	3.218105	3.125553	2.774365	2.336182	2.251757	2.41671	-0.38041
**PACS2**	5.865663	5.713524	5.399756	4.130452	4.774141	4.135333	-0.38081
**TMEM147-AS1**	14.09475	11.99099	13.11273	8.485381	10.4659	11.14344	-0.38129
**CSNK1G2**	49.50069	43.26003	43.31664	27.91245	38.44182	38.01753	-0.3827
**AHRR**	13.10521	12.08802	11.64641	9.486453	8.605043	10.10501	-0.38574
**LTBP4**	21.93788	20.35423	18.75105	12.7129	18.12418	15.88405	-0.38575
**TTC31**	9.582491	8.748543	8.725167	6.122065	7.445453	7.138788	-0.38589
**BBS2**	6.391718	7.229975	6.9813	5.349193	4.856128	5.558988	-0.38619
**TMEM143**	8.622935	7.460786	7.720191	6.678531	6.027409	5.496617	-0.38706
**KLHL29**	4.556076	4.365672	4.416893	3.187409	3.466352	3.543753	-0.38739
**SZT2**	3.321354	3.432392	3.38973	2.328769	2.816971	2.607381	-0.3877
**KCTD15**	18.60563	16.79423	16.44869	12.4724	13.75053	13.40269	-0.38787
**HAUS5**	22.50115	20.34629	20.93887	14.15523	18.10403	16.44828	-0.3891
**CSAD**	5.458507	5.490196	5.624652	3.989164	4.805369	3.85871	-0.38936
**SEMA4C**	13.85012	12.98828	11.6016	8.14857	10.585	10.57666	-0.3912
**FASTK**	36.17305	32.86403	30.68566	21.07742	28.85328	26.04853	-0.39232
**RAVER1**	13.28181	12.62552	12.05682	7.908015	9.731966	11.24667	-0.39423
**HDHD3**	17.62026	16.4935	14.05538	11.91704	11.47159	13.26092	-0.39431
**PIGV**	8.025754	8.442988	7.705237	6.048925	6.383588	5.935928	-0.39623
**LRP1**	9.768859	9.186898	8.357808	6.523578	7.327415	6.893936	-0.39686
**TP73-AS1**	8.332917	8.358098	7.940513	5.627079	6.583878	6.492022	-0.39724
**ADCY6**	15.74605	14.81802	13.47964	10.33038	12.01359	11.08967	-0.39764
**CTNS**	12.42814	11.60327	10.58861	9.409843	8.59906	8.259915	-0.39826
**TECR**	34.04907	33.10206	28.34247	23.91338	24.45195	24.07525	-0.39861
**GPR137**	13.86165	12.98846	13.34502	7.997419	11.17929	11.30749	-0.39896
**PCNX3**	20.73962	18.78436	18.17492	14.05783	14.65549	14.98465	-0.40098
**PNPLA2**	22.95955	20.64187	19.63142	14.69725	16.22278	16.94522	-0.4017
**TPBG**	8.227234	7.670309	6.788982	6.200487	5.441493	5.52755	-0.40199
**HS6ST1**	18.38325	17.44208	16.30782	11.46578	14.53968	13.44845	-0.40203
**LMF2**	63.71552	53.28105	50.1716	33.01074	46.17751	47.2537	-0.40283

**RPP25L**	35.92991	34.4988	29.43588	23.11643	25.5248	26.89314	-0.40284
**KRI1**	23.1394	20.97854	21.28711	13.81609	17.53502	18.07533	-0.40412
**TYK2**	24.48206	21.31329	22.00431	15.70786	18.4825	17.02194	-0.40479
**ZBTB40**	11.18385	10.84137	11.95505	8.752553	8.522562	8.390907	-0.40484
**DGCR5**	3.752697	4.223666	3.907489	3.001959	3.123547	2.849712	-0.40498
**PRDM16**	2.487526	2.704381	2.153423	1.852249	1.81522	1.879243	-0.40519
**MTG1**	6.086525	5.352541	5.696052	3.724314	4.384292	4.827692	-0.40553
**PPIEL**	4.813643	5.074583	4.567267	3.398534	4.143666	3.360711	-0.4069
**TMEM223**	11.82377	10.32694	10.94596	8.580454	7.953163	8.402447	-0.40845
**SREBF1**	19.31879	15.42862	14.48543	11.99809	12.8001	12.29066	-0.40864
**DGCR2**	40.37088	39.70129	34.81833	28.34505	29.23678	28.96043	-0.40878
**RABL2A**	5.21335	4.667188	4.168603	3.370024	3.511246	3.697849	-0.40926
**VEGFA**	34.07124	29.48426	28.04907	18.97186	24.78879	25.20981	-0.40944
**NPTX1**	13.71498	13.66748	13.38651	10.30928	9.361196	11.0215	-0.40961
**ZNF512B**	26.15257	23.72299	23.24971	14.72232	20.48945	19.83321	-0.40976
**HOOK2**	8.060954	7.101403	7.484737	4.98499	6.412027	5.649042	-0.40989
**POLD1**	38.80919	34.39893	33.63318	22.27499	29.37676	28.7643	-0.40991
**MINK1**	16.82702	15.87092	15.3878	9.741608	13.71158	12.73807	-0.40997
**CFAP44**	2.256605	2.333866	2.480087	2.070161	1.78901	1.46115	-0.41031
**ATG2A**	9.618482	8.556535	8.846664	6.381618	7.009233	6.938353	-0.41056
**TMEM234**	8.957559	8.104026	8.038283	5.345877	7.327774	6.199999	-0.41131
**PHYKPL**	17.58318	16.17217	15.69136	11.28536	13.52081	12.3609	-0.41185
**ULK3**	32.95148	29.89142	30.56179	20.65604	25.19948	24.35051	-0.4119
**MAZ**	43.01634	36.12694	36.24103	22.55782	31.26778	32.89138	-0.41206
**CROCCP2**	27.55997	24.40606	24.4377	15.43174	21.59341	20.38805	-0.41226
**C14orf132**	4.437564	5.31338	5.29119	3.300818	3.82165	4.16568	-0.4142
**INPP5J**	5.767054	5.442234	5.824845	3.91534	4.616872	4.234645	-0.41603
**CCDC40**	2.279444	2.273509	2.146896	1.4994	1.847457	1.669895	-0.41737
**MAN2A2**	14.80741	13.6365	12.8462	9.511607	11.04379	10.35296	-0.4178
**SLC5A3**	7.666835	7.906662	7.667564	6.255724	5.885815	5.252203	-0.41811
**JMJD4**	6.864598	5.922942	6.223127	4.984765	4.452012	4.785374	-0.41867
**MAN1B1**	14.20862	14.09525	13.2211	9.361516	11.77732	9.918832	-0.41903
**FAM83H**	9.961105	8.336341	7.517845	5.943568	6.946173	6.411438	-0.41954
**SLC9A5**	5.998368	5.75907	5.996813	3.493699	4.91995	4.85481	-0.42016
**IQCC**	11.11779	10.93569	9.626493	8.043408	8.368238	7.252696	-0.42086
**AATK**	4.918746	4.497616	4.164953	3.00755	3.843096	3.292519	-0.42112
**ATP13A1**	20.23818	16.60058	15.00308	13.1977	13.01084	12.44492	-0.42352
**LBX2**	6.686995	6.684003	7.087405	4.556762	5.617569	5.073948	-0.42405
**HOXD9**	39.92621	32.32489	38.83507	21.7109	30.72287	30.30467	-0.42505
**SLC35B2**	33.45857	31.30574	29.10938	24.81495	22.45159	22.60662	-0.42598
**ZNF707**	5.733695	5.29795	5.289518	3.266607	4.511829	4.369617	-0.42602
**CCDC8**	21.04094	21.67459	19.44124	12.13006	17.66288	16.46993	-0.42606
**TRMT2A**	28.53493	23.9381	23.71	16.0437	20.18259	20.40747	-0.42781

**REEP4**	39.93351	36.95327	33.27426	24.06695	30.12747	27.69183	-0.42792
**DUXAP8**	7.275898	7.149133	7.466829	4.590352	5.965671	5.716838	-0.42793
**AC011815.1**	4.869304	4.982436	4.618622	3.648408	3.154799	3.944291	-0.4291
**SLC38A10**	21.45168	20.62819	17.96646	11.80478	16.83448	15.95756	-0.42914
**SHMT2**	102.9821	87.30985	83.11004	60.23161	71.06508	71.71136	-0.42949
**SLC35E2A**	3.519137	3.502273	3.6873	2.734156	2.618681	2.592873	-0.43054
**LRFN1**	13.85782	13.20636	13.13277	8.803867	10.18225	10.83486	-0.43076
**PIANP**	6.691673	7.301011	6.871851	4.540829	5.33223	5.596476	-0.43162
**P3H4**	38.33431	35.14073	30.37011	24.38258	26.10244	26.33766	-0.43483
**KLC3**	9.836965	9.536737	8.8814	5.541079	8.401966	6.959147	-0.43486
**CTSF**	11.288	10.85029	9.86617	6.628214	8.384788	8.661801	-0.43492
**MEGF8**	18.74024	18.25782	16.57181	9.438617	15.57448	14.59338	-0.43569
**RNF123**	12.26707	11.02105	11.27458	7.883546	8.991281	8.675228	-0.43589
**ENOSF1**	7.913098	7.218861	7.795044	5.822814	5.985608	5.139644	-0.43593
**SLC35E2B**	12.52773	12.89141	12.49002	9.133537	9.244164	9.642798	-0.43606
**GHDC**	12.64248	12.97398	10.49944	7.223094	9.738495	9.73331	-0.43607
**TRABD**	50.46738	43.38013	45.18405	26.03641	37.66684	39.03911	-0.43638
**SDHAP1**	6.915083	7.826559	6.903954	5.238598	4.92615	5.827607	-0.43669
**PCED1A**	36.33204	33.94068	30.38121	19.1624	28.06939	27.066	-0.43801
**TRMT12**	6.472638	6.648295	7.074593	5.572587	4.640536	4.691662	-0.43826
**LRRN2**	4.263634	4.129522	3.919573	2.73086	3.097096	3.257793	-0.43847
**PEX16**	7.560404	6.862652	6.600085	4.154129	5.571419	5.787202	-0.43852
**TMEM259**	64.78558	58.76136	54.67082	33.89445	52.41128	45.19203	-0.4386
**SPIN3**	2.626233	2.359259	2.816891	2.028434	1.88139	1.839396	-0.44055
**SLC16A13**	19.49098	18.89214	16.35274	11.06227	14.1083	15.15256	-0.44088
**IPO5P1**	5.379797	5.115832	5.208105	4.353648	3.358152	3.854332	-0.4412
**FBF1**	5.72004	4.47818	5.149673	3.016299	4.408387	3.875005	-0.44176
**RHEB**	40.86688	34.44719	33.02481	28.47489	25.71015	25.57035	-0.4419
**CASC10**	3.94995	4.426278	3.758541	3.168399	2.975747	2.786665	-0.44228
**AC055811.4**	7.9694	7.981089	7.943825	5.876634	5.424999	6.278643	-0.44271
**HCN3**	12.39237	11.60365	11.43792	8.014182	9.889552	8.165504	-0.44278
**GABARAPL1**	12.67769	16.46632	16.10592	10.83326	11.42016	11.02871	-0.44317
**TMEM38A**	7.27288	6.809691	5.839972	4.795217	4.764744	5.090008	-0.4435
**DOC2A**	5.639606	5.889189	5.331537	3.52727	4.784573	4.082125	-0.44399
**SEC31B**	3.573121	3.386823	3.654996	2.292278	2.680159	2.822861	-0.44542
**SH3YL1**	2.176056	2.082021	2.255015	1.766342	1.632152	1.383648	-0.44569
**PLCXD1**	34.18427	36.36523	34.76323	22.70598	27.98465	26.54658	-0.44731
**AGAP4**	6.205862	5.951978	6.815797	5.3589	4.457411	4.094643	-0.44777
**SUOX**	4.016964	4.12028	3.900422	3.045341	2.939699	2.840413	-0.44781
**ZNF514**	7.3764	7.288926	8.478227	5.326144	6.060979	5.57893	-0.44796
**SLC6A6**	10.16113	9.403235	9.092164	6.371442	7.335688	7.296574	-0.44822
**GALT**	6.998201	6.100432	6.343345	4.307907	5.08219	4.859011	-0.4483
**MSTO2P**	11.81446	10.44571	12.90031	7.293038	8.98209	9.486952	-0.44871

**ZNF331**	2.572753	2.2062	2.519332	1.855906	1.704654	1.786414	-0.44884
**LPIN3**	8.443483	8.040909	7.548364	5.088601	6.701186	5.809467	-0.44949
**SLC4A2**	36.55582	34.28174	31.52755	21.05674	28.12506	25.72924	-0.45047
**STAG3L2**	10.51464	9.439148	8.690987	6.051545	7.713949	7.196367	-0.45051
**PC**	3.319251	3.199726	2.737695	2.200488	2.211268	2.361289	-0.45069
**JMJD8**	19.65119	15.53019	15.29081	11.03046	13.32686	12.56185	-0.45112
**EMILIN3**	18.26579	15.6759	15.34436	12.57946	10.91362	12.5414	-0.4518
**DGAT1**	25.24395	21.24431	18.95658	13.63441	18.00365	16.20817	-0.45187
**FMN2**	5.365129	4.806227	4.581767	3.185457	3.983118	3.614347	-0.45227
**IFT140**	7.877654	8.186707	7.573804	5.247399	6.32489	5.692732	-0.45327
**RGL2**	12.85861	10.64355	10.08872	6.056928	9.004513	9.464937	-0.45374
**SLC46A1**	2.788339	2.545216	2.54276	1.791464	1.843928	2.112446	-0.4545
**MFSD10**	36.2071	31.64326	31.71253	19.82733	26.27124	26.55767	-0.45452
**AC018638.2**	39.73253	42.70365	49.23564	29.62259	28.95754	37.4796	-0.45494
**HERC2P2**	22.88335	20.7319	24.18323	17.04924	16.11062	16.28918	-0.45531
**TUBGCP6**	22.032	19.51471	19.88182	13.27571	16.53167	14.93442	-0.45729
**LINC00205**	17.10414	17.16553	15.75296	9.594426	13.1489	13.68141	-0.45766
**TTYH3**	34.50594	32.5911	28.96385	18.35369	26.13667	25.45502	-0.45772
**THOC6**	23.61929	20.09302	17.32912	15.49422	15.32337	13.60968	-0.45834
**AL845472.2**	5.29058	5.23325	4.979664	3.06624	4.376263	3.84073	-0.45841
**FKRP**	13.39886	11.02053	9.91903	8.104286	8.109365	8.767578	-0.45898
**BORCS8**	4.474461	4.167827	3.800293	2.63341	3.291953	3.117748	-0.46039
**GRAMD4**	10.93115	11.45394	10.84956	6.093321	9.3168	8.727969	-0.46138
**DDX12P**	38.4604	33.4257	35.83418	22.03885	32.15152	23.96568	-0.46286
**WDR27**	8.085094	7.886387	9.035446	5.826175	6.65966	5.655443	-0.46305
**ZNF444**	11.1925	9.562587	9.617799	5.760986	8.159634	8.103072	-0.46373
**POU4F1**	32.45788	28.16959	29.52803	20.1477	23.70067	21.5203	-0.46382
**PKD1**	13.92625	12.03306	12.07497	7.576893	10.35398	9.646333	-0.46382
**ZNF780B**	2.194629	2.331622	2.612012	1.962777	1.748229	1.464495	-0.46387
**SLC52A2**	19.56547	18.14237	16.19284	10.22059	14.67452	14.1795	-0.46407
**SLC26A11**	14.21716	13.54234	13.27384	8.698836	10.57337	10.47411	-0.46409
**GABRE**	1.588911	1.710257	1.692719	1.214623	1.307488	1.094011	-0.46514
**FBXL8**	4.2887	4.26731	4.536111	2.873121	3.025739	3.579532	-0.46598
**DVL1**	34.28428	31.17017	30.38203	16.49122	28.06733	24.80401	-0.46642
**SPC24**	24.99614	23.38785	21.52633	15.13418	18.84535	16.59389	-0.46713
**IFFO1**	13.62485	13.00012	12.2034	7.509952	10.68835	9.884879	-0.4674
**NSUN5P1**	13.68142	11.62135	11.86915	8.037898	9.829469	9.008532	-0.4679
**SMOC1**	11.46041	10.59224	10.54645	8.12501	7.879646	7.561964	-0.46809
**PIEZO1**	26.17267	23.57728	21.68348	15.04764	19.3417	17.24669	-0.46822
**SDK1**	1.085294	1.078942	1.367176	0.810953	0.840974	0.90033	-0.46847
**CBX8**	7.451001	7.001327	7.094736	4.0036	5.669808	5.886406	-0.46967
**PLEKHM1P1**	6.126169	5.302581	5.701041	3.961783	4.504848	3.901077	-0.46993
**ARFRP1**	6.220658	5.023579	5.058555	3.123537	4.419565	4.22646	-0.47006

**GATA2-AS1**	14.3675	12.13228	13.38442	8.730427	9.692468	10.35108	-0.47105
**TECPR1**	4.552101	4.306486	4.255636	2.500137	3.688365	3.268804	-0.47163
**POLN**	1.51827	1.336249	1.587608	1.042126	1.075453	1.08461	-0.47219
**MST1**	5.658971	5.473332	4.900214	2.88603	4.696772	3.971287	-0.4726
**SCNN1D**	7.974753	7.902543	8.106586	4.076532	6.880383	6.308195	-0.47421
**MOGS**	41.56089	36.06111	32.54092	19.26633	30.46965	29.54322	-0.47462
**ESPNL**	3.613831	3.640676	3.45378	2.314661	2.871182	2.517392	-0.47519
**CCS**	21.29995	20.14175	20.22872	13.58324	17.5618	13.21507	-0.47532
**AL133325.3**	13.4371	11.94342	13.10556	9.390028	9.370215	8.919584	-0.4755
**CNTNAP3**	1.747807	1.937933	1.824676	1.452698	1.296413	1.212906	-0.47593
**DDR2**	3.163781	3.347389	3.114882	2.538822	2.158457	2.223759	-0.47596
**CDK10**	14.39763	11.83245	11.54437	7.442037	10.21147	9.476616	-0.47752
**COL5A1**	4.124646	4.422935	3.972294	2.594478	3.329509	3.062102	-0.47845
**PITPNM1**	8.761821	7.746802	7.370527	4.925459	6.222122	5.988691	-0.4787
**C2orf48**	6.414545	5.526615	5.88615	4.203827	4.682163	3.897228	-0.47984
**CYB5D2**	14.04958	16.20575	15.29725	10.12271	10.98449	11.54871	-0.48019
**ABCA7**	7.310198	5.802127	5.522855	3.802663	5.235684	4.318819	-0.48041
**ITGA8**	2.657548	2.940051	3.004761	2.20056	1.776765	2.184197	-0.48145
**SH2B1**	13.95343	12.52241	13.47174	7.007077	11.33883	10.26477	-0.48155
**EVI5L**	7.032768	5.813433	5.656236	3.162795	5.226438	4.856288	-0.48221
**PTOV1**	21.05151	18.78407	18.76293	10.39096	15.88488	15.66875	-0.48238
**SLC10A3**	20.57171	19.28064	17.09178	11.35118	14.32219	15.06063	-0.48331
**ACP2**	17.67256	15.78952	14.40245	10.94365	10.70573	12.52306	-0.48612
**LENG8**	83.54017	72.97333	76.1493	41.07848	66.25431	58.75599	-0.48629
**CPNE1**	16.84995	18.0708	17.54931	12.53528	13.14928	11.75352	-0.48699
**AP006621.5**	19.41516	19.88273	17.73021	10.89659	15.05334	14.73818	-0.48707
**CES3**	9.111918	9.004387	8.702498	6.223402	7.048682	5.857841	-0.48741
**GGT1**	6.330084	5.619158	6.075999	4.044463	4.474247	4.320609	-0.48945
**AP1G2**	9.050564	8.478801	8.048921	5.749278	6.455722	5.998877	-0.49067
**CCDC57**	12.26591	11.08492	10.92995	6.85955	9.322964	8.210968	-0.4909
**RAD51-AS1**	7.779836	8.121488	8.486483	6.111732	5.896591	5.339715	-0.49139
**SLC37A4**	17.09698	16.80284	16.17323	9.938178	12.74839	12.93167	-0.49142
**AC073957.3**	1.822172	1.723037	1.748593	1.128371	1.222061	1.414571	-0.49165
**SCAND1**	24.1035	21.19046	20.16891	10.88157	17.7423	17.93157	-0.49173
**PGPEP1**	3.77665	4.013426	3.254223	2.035061	3.199192	2.61874	-0.49199
**TNK2**	6.595714	6.117733	6.110301	3.133945	5.424206	4.824789	-0.49216
**GPAA1**	48.92908	42.61338	37.21212	24.63743	33.82795	32.93816	-0.4943
**MIGA2**	8.778425	9.231771	8.633938	4.998626	7.343002	6.565258	-0.49491
**NFKBID**	4.729424	4.082868	3.915724	2.581805	3.326314	3.120793	-0.49538
**COMTD1**	22.44527	18.04518	19.17219	10.31827	14.97086	17.01379	-0.49607
**ABTB1**	3.205429	2.86838	2.832466	1.949991	2.262823	2.101915	-0.4961
**PIDD1**	15.07164	14.51553	14.34214	7.401472	12.45806	11.23901	-0.49834
**CLCN7**	17.68594	15.81313	14.61448	10.33143	12.64873	11.0774	-0.49847

**TMEM132A**	19.55026	17.16281	14.69371	9.18569	14.76592	12.41666	-0.49928
**CALCOCO1**	7.260456	6.783763	6.783959	4.160854	5.528554	5.036608	-0.50017
**PCDH7**	9.81778	9.342052	9.089351	7.303077	6.185363	6.480815	-0.50043
**VPS9D1**	7.842545	7.730255	7.241687	4.893866	5.852751	5.365463	-0.50181
**ANO8**	10.80648	10.26612	10.13109	5.19667	8.600023	8.232952	-0.50227
**PPFIA4**	17.34888	16.39274	15.84395	9.903606	12.97466	12.05029	-0.50551
**CEP131**	18.34066	18.56606	16.71008	8.128491	15.33486	14.28889	-0.50612
**SLC1A1**	2.451131	2.75191	2.338112	1.933645	1.740132	1.635521	-0.50626
**PORCN**	7.258387	7.165785	6.633758	4.907923	5.053384	4.863971	-0.5063
**EML5**	0.860619	0.961276	0.994102	0.611955	0.661022	0.709154	-0.50659
**SGSH**	12.53773	11.04524	11.42234	7.324823	9.454229	7.855079	-0.50692
**SPDYE6**	10.05953	9.923992	9.804295	7.370669	7.140063	6.444722	-0.5074
**PLEKHJ1**	19.43122	16.49859	15.49658	10.81171	12.41908	12.92147	-0.50842
**IL9RP3**	10.33841	8.464796	10.0619	5.466332	8.177824	6.647226	-0.50846
**CDC42BPG**	3.539451	3.880345	3.304108	1.89296	3.054945	2.59012	-0.50857
**POLM**	9.040948	9.082906	8.962123	5.367989	6.742131	6.928471	-0.50862
**ZBTB12**	11.37989	10.7995	9.336012	5.609587	8.282629	8.259555	-0.50864
**MRNIP**	16.75316	14.57229	14.60433	10.92528	11.19463	10.14927	-0.50927
**MRC2**	18.41438	17.03034	15.5162	8.977077	14.68878	12.12378	-0.50985
**MXRA8**	18.39796	17.19723	16.47106	8.363272	14.63507	13.56618	-0.5099
**TONSL**	25.24771	21.89559	20.84059	14.31042	16.77713	16.6538	-0.50995
**ZNF696**	8.083549	7.255911	6.814012	3.840687	5.962929	5.728177	-0.51231
**FBXL6**	15.01102	11.35254	12.01949	6.84058	10.8121	9.233934	-0.51358
**CROCCP3**	3.240405	3.034958	3.566033	2.03011	2.59568	2.267493	-0.51367
**KIF26A**	13.70592	12.78086	12.3482	6.935699	10.70949	9.542691	-0.51439
**TMEM250**	41.07404	35.30932	34.43325	18.78431	30.64971	28.12728	-0.51477
**NPDC1**	48.58568	45.43759	40.59737	20.21358	39.39051	34.50842	-0.51644
**MTA1**	36.8523	34.12007	33.99725	21.08578	27.60964	24.63063	-0.51757
**MYO15A**	0.576826	0.53935	0.566542	0.315508	0.457773	0.401466	-0.51844
**PSKH1**	13.52916	13.72456	12.24012	8.502714	9.344759	9.720721	-0.51862
**EML3**	10.29908	10.01236	9.766375	5.326043	8.052992	7.59898	-0.51982
**EPS8L2**	3.786961	3.256047	3.586014	1.921429	2.885581	2.606114	-0.51986
**SHROOM1**	8.726206	7.235063	7.451959	4.378524	5.818697	6.116459	-0.52124
**MTMR11**	3.501804	3.500238	3.508516	1.994312	2.843387	2.464308	-0.52547
**ACAP3**	14.48291	12.05713	13.03426	6.84164	10.72553	9.910195	-0.52632
**RFNG**	17.21254	16.19354	14.97916	8.136804	12.80912	12.64399	-0.52654
**TMEM104**	8.749562	8.161626	6.636552	5.023637	5.39547	5.924195	-0.52689
**CCDC184**	5.902863	5.444875	5.005892	3.411721	3.333269	4.602351	-0.52726
**STX4**	13.96487	13.33216	12.80196	5.983743	11.53473	10.30513	-0.52726
**PAQR4**	16.36215	15.09581	13.97572	7.414253	11.5684	12.53179	-0.52775
**PCK2**	53.50537	47.42913	44.01957	28.03371	37.37313	35.0255	-0.52937
**SLC25A29**	15.53435	14.03631	13.25579	7.545106	11.47556	10.64381	-0.52977
**SYT3**	3.24914	3.456417	3.466963	2.428293	2.285659	2.330643	-0.53009

**LAMB2**	40.64266	36.46167	32.76967	24.21509	26.81283	24.98743	-0.53149
**DGKQ**	12.11327	9.94016	11.35601	5.629339	9.021993	8.447902	-0.53241
**SEMA3G**	2.50172	2.272486	2.551795	1.269881	1.952655	1.839059	-0.53343
**EFNB3**	5.853398	7.201413	5.706669	3.035915	4.875688	5.043898	-0.53421
**BRSK2**	14.04082	11.59085	10.8269	6.65849	9.460596	9.045888	-0.53484
**CHST14**	14.86845	14.74509	12.90474	9.564472	10.27288	9.500825	-0.5353
**ATG16L2**	5.664756	4.441456	4.323871	3.09424	3.547189	3.314877	-0.5354
**TLE2**	4.505699	3.498578	4.073002	2.500559	3.20125	2.61364	-0.53843
**TM7SF2**	12.89081	12.86306	10.68692	6.820636	9.126978	9.088419	-0.54155
**CCNL2**	57.01352	52.80301	53.98688	37.71884	37.56512	37.20596	-0.54217
**TPCN2**	6.537888	5.770153	5.415713	4.101632	4.203391	3.852502	-0.54383
**CBSL**	10.4548	7.473552	8.072017	5.647764	6.367187	5.806473	-0.54492
**ZNF316**	16.45027	12.93652	12.99048	6.733242	11.47015	10.82475	-0.54584
**TRIM46**	4.446048	3.966501	4.309136	2.703164	3.158627	2.842805	-0.54744
**ABCC10**	10.26172	8.844797	8.389883	5.178961	6.996669	6.594671	-0.55079
**CTC1**	5.176122	4.677801	4.648168	3.253868	3.442553	3.196229	-0.55183
**AC022098.1**	4.549919	4.098246	4.387182	2.244891	3.536074	3.108543	-0.55225
**MIR210HG**	15.08823	13.75966	14.24814	8.06635	10.92614	10.39578	-0.55231
**ARSA**	14.60522	14.48411	13.70019	6.654449	11.4966	11.00732	-0.55335
**MIEF2**	9.764356	9.427017	8.265402	5.167562	7.294105	6.24305	-0.55376
**LMF1**	1.576567	1.74193	1.601346	1.002868	1.330225	1.017159	-0.55434
**RECQL5**	10.3987	9.332611	9.67275	6.377258	6.963552	6.639205	-0.55746
**SLITRK2**	1.441091	1.434082	1.740572	0.94282	1.182455	1.010601	-0.5577
**PNPLA7**	1.682917	1.373212	1.560236	0.854006	1.336267	0.942935	-0.55912
**TSNAXIP1**	2.073628	2.289417	2.483966	1.378914	1.619653	1.647877	-0.55935
**STX1B**	3.84719	4.445546	3.878925	2.20256	3.519319	2.537545	-0.55941
**ASB16-AS1**	12.11611	9.03184	10.66483	6.477534	7.207014	7.896443	-0.55985
**MBD6**	12.56236	11.33559	12.33143	5.663239	9.277337	9.617239	-0.56098
**ALDH16A1**	7.327445	6.823026	6.666695	3.658525	5.722903	4.719096	-0.56202
**CIAO3**	4.122043	3.820706	3.722971	2.220188	2.781368	2.898229	-0.56239
**TMEM175**	13.72832	12.58419	12.19471	7.765826	9.452563	8.823499	-0.56429
**AC118344.2**	3.732893	3.39604	3.340538	2.019716	2.571539	2.487721	-0.56458
**FZD9**	7.512985	7.012909	6.412193	4.476213	4.837817	4.841168	-0.5648
**AMH**	31.5907	25.55023	27.25362	13.17288	21.99967	21.84645	-0.56571
**PMEL**	6.360774	5.958924	4.882924	4.394848	3.52078	3.705261	-0.56591
**WDR90**	15.07764	12.4073	13.04296	7.373766	10.53291	9.430805	-0.56804
**PIGQ**	15.8558	14.00095	13.42769	7.824608	10.78379	10.50306	-0.57226
**SCN1B**	1.77658	1.435387	1.500484	0.796119	1.190473	1.176869	-0.57497
**LRFN3**	7.507501	6.944431	5.560722	4.980693	3.995625	4.453615	-0.57546
**MCOLN1**	8.347541	7.575054	7.512349	5.463711	5.434663	4.823332	-0.5759
**B3GALT6**	32.61988	28.04837	24.99035	18.50731	19.62089	19.24155	-0.57831
**HEXDC**	11.8651	10.12504	10.12756	5.744237	8.075398	7.671315	-0.57964
**NEIL1**	11.69407	10.83513	10.41638	5.594023	8.662428	7.787728	-0.57969

**CARMIL3**	2.297014	2.138888	2.289012	1.270363	1.697047	1.531842	-0.57983
**CNPY4**	11.34044	11.48515	10.2617	5.460848	9.013108	7.632286	-0.58182
**GIGYF1**	32.36593	28.52548	30.22845	15.23639	23.27593	22.35611	-0.58207
**C19orf57**	8.060419	9.398105	8.393794	4.601615	6.440413	6.203828	-0.58404
**AP5S1**	2.694332	2.222371	2.489783	1.417724	1.618774	1.901486	-0.58487
**ASH1L-AS1**	5.964468	6.161223	6.965932	3.784384	4.417881	4.522855	-0.58526
**B4GAT1**	79.22437	78.88422	65.48018	49.03651	50.40806	49.49621	-0.58611
**SAMD11**	19.47515	19.61691	19.44294	9.265716	14.41552	15.30098	-0.58648
**SAMD10**	5.487408	5.008738	5.877902	2.701315	4.00719	4.193317	-0.58684
**DOLK**	16.91512	14.27679	12.34189	11.52448	8.139286	9.312674	-0.58726
**STUM**	1.518869	1.873722	1.811044	0.931511	1.376886	1.153394	-0.588
**ZNF879**	2.133261	1.929366	1.998561	1.692953	1.202152	1.132487	-0.58968
**LRRC45**	23.91477	23.00764	22.51128	13.25685	15.52151	17.34844	-0.59003
**TMPRSS6**	2.385505	1.88153	1.875422	1.118405	1.431797	1.527017	-0.59123
**UNC5B**	15.81138	14.54948	14.81807	8.417763	10.71421	10.84948	-0.59158
**SLC7A3**	27.18375	23.49345	19.66189	16.578	15.89277	14.19812	-0.59187
**ZNF358**	14.92414	15.18726	13.70322	6.659521	10.54312	11.86527	-0.59199
**AC090114.2**	2.191312	2.069488	2.094023	1.118299	1.660728	1.436525	-0.59213
**AARSD1**	2.136516	1.722104	1.845155	1.249436	1.272365	1.259481	-0.59304
**AP001107.9**	3.129943	2.76788	3.421028	1.937088	2.216069	2.022814	-0.59349
**NME3**	42.35391	39.55099	37.22752	19.03426	31.62083	28.18284	-0.5956
**HEXIM1**	42.50611	35.12788	36.97709	23.32946	25.93309	26.54402	-0.59635
**TMEM198**	8.692669	9.332486	7.628236	3.753506	6.389642	6.823039	-0.59649
**MAPK8IP3**	15.78783	14.61405	15.10789	7.782382	11.93566	10.37668	-0.59667
**TSPEAR-AS2**	6.428065	5.772118	6.455159	4.014627	4.093865	4.220661	-0.59752
**AMDHD2**	5.234415	4.80459	4.443663	2.264521	4.009353	3.275599	-0.60083
**NXPH4**	21.74062	22.55981	19.33951	10.59136	15.9913	15.36661	-0.60129
**AC253536.3**	12.65793	14.11652	13.82853	7.822509	8.928575	10.0051	-0.60171
**DNASE1L1**	9.706053	9.028427	8.679858	4.63504	6.368555	7.055176	-0.60223
**RABEP2**	4.049464	4.303489	4.046227	2.061671	3.057158	3.048375	-0.60233
**SPACA6**	2.595699	2.493678	2.278277	1.102298	2.034721	1.714823	-0.60267
**PAQR8**	7.371078	8.262297	8.96339	5.834076	5.387523	4.960367	-0.60408
**D2HGDH**	9.919162	8.750115	9.20454	4.712069	7.754192	5.867273	-0.60443
**SDHAP3**	22.57244	21.49618	21.80229	14.3916	13.96652	14.96296	-0.60457
**SLC22A17**	11.24605	10.1671	10.58527	5.728585	7.963324	7.347405	-0.60491
**MIB2**	10.70965	8.628385	8.52996	4.870878	7.003715	6.420365	-0.60716
**OSTN**	1.952951	2.88591	2.534095	1.764432	1.575618	1.496761	-0.60819
**ZNF784**	4.258595	3.910717	4.542162	2.580942	2.83525	2.91714	-0.60917
**C3orf80**	3.823141	4.425892	4.007003	1.99292	3.269709	2.771531	-0.60927
**CFD**	10.47052	10.64992	10.78877	5.938955	6.868529	8.104598	-0.60964
**ZDHHC1**	9.673221	8.42895	7.697636	4.575617	6.212783	6.081463	-0.61291
**PBXIP1**	14.67281	14.96289	12.9612	7.005611	10.71113	10.07849	-0.61591
**FNDC10**	18.46325	17.69885	16.26269	9.988294	12.55035	11.64583	-0.61691

**ADAM11**	14.28021	14.95204	14.14312	6.615079	11.87502	9.788141	-0.61718
**PHETA1**	5.512297	4.623619	4.631038	2.174946	3.952907	3.492792	-0.61817
**TMEM115**	45.12635	43.35547	35.84458	20.87318	28.07049	31.9729	-0.61963
**MORN1**	4.08711	3.651508	3.729861	1.813764	2.957439	2.688209	-0.62054
**CHST2**	7.013392	5.633862	5.639163	3.707231	4.202382	3.964071	-0.623
**CHTF18**	31.01644	28.56886	27.56201	13.61861	23.51185	19.42959	-0.62367
**PRSS30P**	7.256064	6.15907	6.328819	2.557563	5.335776	4.920276	-0.62373
**TEDC1**	6.713544	5.975998	5.299353	2.38688	4.972241	4.298855	-0.62579
**LINC00894**	3.397945	3.960743	4.009746	2.154164	3.229844	1.965455	-0.62932
**SPPL2B**	21.4669	18.24673	17.94033	9.662376	14.6991	12.87137	-0.63084
**POLR3H**	15.10257	12.80271	12.38409	8.071119	9.284506	8.646326	-0.63178
**AC012313.3**	2.07571	1.67073	1.625215	1.266921	1.329982	0.865921	-0.63342
**AC110285.2**	9.47531	8.89023	10.56548	3.629206	7.686238	7.305665	-0.63568
**BX255925.3**	25.20815	23.4894	22.20823	10.87592	17.62468	17.0973	-0.63694
**FAAP100**	20.02319	17.5989	17.0394	10.12704	13.22885	11.763	-0.63828
**CHST12**	4.377638	4.200634	3.365105	2.580139	2.76651	2.32611	-0.63839
**POLR2J3**	5.170203	4.844629	5.031331	3.396086	3.250234	2.996762	-0.64183
**BAHCC1**	9.765737	9.567322	9.271594	4.894805	6.659503	6.777553	-0.6419
**FAM173A**	13.40346	10.48541	8.52974	4.731175	9.149559	6.860294	-0.64434
**DISP2**	0.904631	0.765301	0.653413	0.519549	0.429345	0.536673	-0.64519
**KCNC3**	6.24478	5.428813	5.739077	2.798315	4.404757	3.917074	-0.64696
**TFRC**	19.54715	23.76336	22.82652	16.24305	12.77609	13.21167	-0.64716
**RECQL4**	54.518	47.48905	45.59217	26.12113	36.06841	31.98292	-0.64831
**OBSCN**	2.218324	2.273736	2.176669	1.149844	1.695841	1.406861	-0.64908
**SYP**	12.38093	10.87691	9.740255	4.527873	8.675601	7.816383	-0.65063
**CCDC157**	1.662009	1.638033	1.593409	0.78254	1.213086	1.114763	-0.65376
**STAG3L3**	5.165386	4.910683	4.501287	1.977172	3.904579	3.37894	-0.65454
**THBS3**	5.319382	5.037545	5.035959	2.843926	3.922391	3.01079	-0.65478
**PKMYT1**	32.07258	27.87659	27.26704	12.32739	21.85362	21.16269	-0.65618
**LAMP1**	41.02512	39.26589	38.26512	26.36713	25.64564	23.20293	-0.65646
**MELTF-AS1**	8.441372	8.494321	9.136091	3.50907	7.290232	5.740838	-0.65652
**ZNF865**	10.93233	9.352969	8.818651	4.700918	6.43284	7.313566	-0.6578
**TMEM80**	15.7057	16.70963	14.80606	9.12023	11.20175	9.599591	-0.65825
**SSPO**	0.482709	0.471751	0.510918	0.203952	0.371488	0.352655	-0.65893
**SMPD3**	3.183991	2.825715	3.027335	1.254936	2.536714	1.931678	-0.659
**SRPK3**	3.515566	3.113281	3.504038	1.230497	3.046524	2.136014	-0.65997
**MIR34AHG**	2.72822	2.80906	2.498043	1.353727	1.934219	1.788456	-0.66255
**NPEPL1**	1.117957	1.01408	0.969463	0.509312	0.910489	0.535472	-0.6656
**AC087741.1**	4.136933	3.560525	3.615612	2.084292	2.663958	2.381519	-0.66606
**IGFBP5**	4.334007	7.210294	5.482567	2.783786	3.825119	4.113537	-0.66718
**PRRT3**	5.482647	5.466709	5.059698	3.284364	3.612157	3.18413	-0.6673
**AL391244.1**	20.67934	17.24263	18.23103	8.789279	13.93632	12.63012	-0.66742
**TAF1C**	17.60465	16.24273	16.46876	8.845133	11.49267	11.32008	-0.66846

**FOSB**	1.624896	1.379969	1.421338	1.032288	0.840619	0.908969	-0.67001
**RHPN1**	19.51805	16.6232	15.71588	8.256561	12.98543	11.31029	-0.67178
**AC138932.1**	4.455973	4.262184	4.383502	2.063556	3.417244	2.687345	-0.68167
**TM9SF1**	3.336121	3.147785	3.104983	1.517016	2.1452	2.303405	-0.68469
**MT-RNR1**	3736.229	2920.719	2509.433	1690.318	1716.938	2283.579	-0.68771
**HOXD-AS2**	33.58968	26.66255	31.504	13.06467	23.02317	20.87206	-0.68786
**KIFC2**	15.94942	13.35221	12.98902	6.14948	10.85012	9.235067	-0.68886
**AL118506.1**	6.947193	6.542327	6.309255	3.890491	3.961226	4.404521	-0.69189
**SARS2**	1.579449	1.351472	1.596692	0.765003	1.083555	0.946795	-0.69572
**HSD11B1L**	6.874194	5.181269	6.014727	2.778068	4.036726	4.321188	-0.69838
**WASH7P**	9.689722	10.07664	9.562786	3.659104	7.2426	7.160908	-0.69933
**ZSCAN16**	3.202309	3.642708	4.050736	2.534452	2.129295	2.044387	-0.69978
**FIBIN**	3.091183	2.208972	2.570969	1.573897	1.551724	1.716597	-0.7009
**AC012313.5**	2.100952	2.228086	1.699263	1.149163	1.266101	1.276142	-0.70758
**EPPK1**	1.823334	1.458029	1.352943	0.828187	0.914401	1.094445	-0.70797
**C1QL4**	13.07057	13.47757	12.7319	5.358023	9.709375	8.918051	-0.71164
**DUXAP10**	9.642177	9.204593	8.366651	4.878373	5.833377	5.889086	-0.71306
**DEXI**	1.609978	1.581993	1.556853	0.967767	0.933408	0.995441	-0.7132
**MAFA**	2.643517	2.102881	2.359623	1.113124	1.7208	1.496562	-0.71451
**DNAAF3**	4.479673	3.552364	3.338375	1.656374	2.569083	2.686667	-0.71808
**LINC00893**	3.775952	3.824856	3.707063	1.896971	2.71497	2.259944	-0.71855
**RAB24**	13.37716	12.70821	10.65498	4.966115	9.541965	7.795877	-0.72007
**ADCK5**	8.267162	6.791501	7.68657	3.490649	5.630985	4.685703	-0.72013
**TEPSIN**	5.086456	4.360099	4.055616	2.286036	3.082135	2.818517	-0.72184
**PPP1R12C**	19.4232	15.81405	16.09409	7.167635	12.34518	11.53694	-0.72526
**MBD3**	23.20129	21.48406	20.03166	10.46435	14.70393	13.94042	-0.72666
**C8orf82**	9.241845	7.746005	7.302348	3.399679	5.997521	5.27518	-0.72727
**BCHE**	9.190932	8.473077	8.040366	6.774232	4.165113	4.528592	-0.73273
**SLC6A9**	13.57619	11.84138	10.75195	6.024543	7.995458	7.693691	-0.73617
**PAQR6**	23.3947	18.53515	19.47045	8.732637	14.40931	13.68691	-0.73741
**ASMTL-AS1**	12.63127	7.984763	10.47008	3.702958	8.976837	5.957787	-0.73806
**ASB16**	4.427615	4.415368	4.468644	1.792301	3.401537	2.76073	-0.74283
**TMEM121B**	2.48039	2.010012	2.026776	1.213852	1.469871	1.191894	-0.74982
**RAB26**	4.533283	5.265202	4.909096	2.219428	3.409611	3.104588	-0.75191
**DPP7**	12.52327	10.61138	11.80511	4.04681	9.517994	7.173143	-0.7526
**C20orf204**	3.023158	2.015501	2.526511	1.170797	1.900206	1.416506	-0.75346
**PRR22**	6.982325	4.859371	5.151537	3.086671	3.479394	3.451221	-0.76247
**TTYH1**	0.784798	0.684425	0.761089	0.334825	0.56518	0.411169	-0.76639
**KREMEN2**	7.129981	6.436221	6.291807	2.454463	4.96233	4.256869	-0.76646
**CACNG8**	4.701204	4.204148	4.889729	1.911922	3.396974	2.797846	-0.76696
**CASKIN1**	6.54947	5.524793	5.563133	2.268268	4.237665	3.805038	-0.77446
**ALOX12**	3.632945	3.812959	3.590162	1.527841	2.60795	2.309523	-0.7759
**AC093249.6**	6.925641	7.868366	6.606306	2.51955	4.887216	5.062961	-0.7792

**AC012085.1**	18.8524	17.01203	14.06373	7.424454	9.739575	11.89648	-0.78079
**MZF1**	7.853558	5.98225	5.970481	3.43473	4.018178	4.051894	-0.78372
**SHC2**	8.915948	8.103927	8.133396	3.755511	5.660775	5.192907	-0.78387
**CD19**	3.287388	3.205609	3.448089	1.125125	2.466892	2.129857	-0.79692
**ZNF503-AS2**	15.3088	13.45161	13.53879	6.550173	8.995606	8.54317	-0.81226
**CHPF2**	25.37133	20.92135	21.24445	13.35695	12.37117	12.70379	-0.81338
**EME2**	10.53887	8.928793	9.317723	4.338722	6.197643	5.827954	-0.81478
**AC126755.1**	2.244653	2.044857	3.110635	1.027992	1.424895	1.736589	-0.82078
**C11orf95**	14.46113	12.56658	12.32802	5.526005	8.376168	8.376766	-0.82089
**LRRC10B**	2.699735	2.006411	2.086432	1.421989	1.213633	1.206268	-0.82214
**AP000525.1**	20.56796	20.7264	20.45825	7.805126	13.71102	13.38905	-0.82306
**NOXA1**	23.02967	19.51702	18.34153	6.738274	15.16647	12.48796	-0.82406
**THEM6**	15.96603	15.35831	12.629	7.212818	8.154083	9.450149	-0.82464
**TSPEAR-AS1**	2.512421	2.326821	2.265513	1.317538	1.315078	1.363928	-0.83003
**MC1R**	2.883433	2.476604	2.297349	1.048774	1.693439	1.559501	-0.83194
**RUBCN**	4.021566	4.266985	4.526541	2.485922	2.291345	2.390752	-0.8382
**ZNF595**	1.288131	1.317059	1.535921	0.521486	0.810266	0.981975	-0.8398
**AL928654.2**	29.74996	27.89857	25.06875	9.099822	16.54159	20.53718	-0.84096
**BMS1P22**	4.916513	4.193843	4.179322	1.739614	3.055094	2.60276	-0.8452
**GPT**	6.427169	5.452201	5.247673	2.308917	4.071497	3.080184	-0.85627
**C2CD4C**	1.701846	1.942794	1.902394	0.504217	1.34839	1.197632	-0.86279
**UNC5A**	1.501585	1.414229	1.284662	0.54291	0.978206	0.780739	-0.86776
**LINC00235**	4.759926	4.522304	4.677447	2.170821	2.968373	2.494934	-0.87073
**ASPDH**	4.272912	4.273218	5.655033	1.399852	3.557065	2.752815	-0.88126
**TRIM73**	1.000533	0.972952	0.803286	0.493267	0.501952	0.510401	-0.88305
**AC118344.1**	10.54756	8.696671	10.07329	4.100716	5.833102	5.911041	-0.88775
**FBLL1**	6.183571	5.016562	5.166951	1.904176	3.723112	3.096351	-0.9078
**SPDYE2B**	3.694401	2.851237	3.63897	0.989867	2.664457	1.716312	-0.92323
**LINC02593**	12.44235	14.16685	14.31544	6.662398	7.1672	7.72694	-0.92484
**MFSD3**	35.70174	32.9939	29.16976	10.35643	20.85404	19.98983	-0.93465
**FAM157C**	1.234292	1.305872	2.553588	0.860224	0.982214	0.819883	-0.93604
**STPG3-AS1**	3.326217	3.397609	3.551309	1.174847	2.24289	1.93329	-0.94127
**COL11A2**	1.426517	1.530739	1.916814	0.546186	1.152072	0.828316	-0.94794
**CCDC107**	4.714342	4.25458	3.989246	0.829039	2.214984	3.643027	-0.95442
**RTEL1-TNFRSF6B**	0.947572	0.880397	0.96013	0.364364	0.626888	0.433145	-0.96893
**RAB7A**	126.9954	133.5249	129.5667	66.84112	66.65108	65.60925	-0.97029
**TSPAN10**	6.93613	5.259886	4.748504	2.116895	3.045882	3.279904	-1.00505
**CBS**	15.09487	21.90331	15.84784	6.602797	9.941193	9.449789	-1.02363
**SLC26A1**	2.170716	1.665327	1.70498	0.83301	0.867782	1.015423	-1.02856
**SLC9A3**	4.639689	3.669418	3.865496	1.147925	2.803768	2.008634	-1.03041
**CCDC189**	5.353115	4.474125	4.035799	1.211906	3.292381	2.234003	-1.04079
**SLC9A3-AS1**	28.315	24.43009	24.59875	6.845731	15.94213	14.33728	-1.05889

**AC018665.1**	6.570549	6.474915	6.118667	2.74147	2.932961	3.509424	-1.06124
**ATG16L1**	11.2585	13.65985	14.25562	5.895226	6.449445	6.212577	-1.07791
**RAB33B**	6.025816	6.322558	5.852213	2.566809	3.121957	2.758632	-1.10741
**ATG12**	11.02586	12.36956	13.6995	5.596051	5.649863	5.839082	-1.11849
**TRAF5**	4.045552	4.128197	4.706473	2.262212	1.932552	1.665896	-1.13602
**ULK2**	8.265984	9.269855	9.128846	3.696843	4.040473	4.287031	-1.14897
**TRAF1**	2.230595	1.93836	2.352005	1.02002	0.955552	0.922151	-1.17016
**CXCL2**	0.493063	0.58791	0.627878	1.658113	1.582655	1.667459	-1.1938
**ATG13**	25.65236	29.26524	28.62358	12.48151	12.19296	11.62636	-1.20249
**COL7A1**	6.850815	6.446036	6.499882	1.714836	3.547436	2.687173	-1.31634
**CRTC1**	5.649161	4.726604	5.102536	2.121521	1.958515	2.026512	-1.34182
**TSC1**	6.895658	7.521659	7.785585	2.765073	2.879937	3.043938	-1.35349
**FAS**	1.530314	1.5602	1.783221	0.665559	0.602256	0.632255	-1.35898
**TERT**	2.52317	2.437242	2.500726	0.954563	1.02366	0.925127	-1.36168
**ATG4C**	28.2257	30.39657	33.65921	11.62899	11.5628	11.45574	-1.41329
**ADCY7**	3.072358	2.956511	2.820333	1.02638	0.951236	1.125125	-1.512
**IL1R1**	0.308034	0.296158	0.305036	0.095213	0.102365	0.112582	-1.55163
**ZFP36**	3.385207	3.045678	3.224731	1.02582	1.023655	1.122252	-1.6061
**BOLA2B**	11.35128	0.407713	5.814495	0.500856	0.427468	0.700342	-3.43164

Appendix Table S4. Relationship between WDR6 expression and clinicopathological features of HCC patients (n=110)

variable	Relative WDR6 Expression		P-value
	**Low (n=53)**	**High (n=57)**	
**Age**			0.702
≤60	28	33	
>60	25	24	
**Gender**			0.838
Male	37	38	
Female	16	19	
**Histological differentiation**			0.258
Well/Moderately	22	30	
Poorly	31	27	
**Tumor size**			**0.004**
≤5cm	33	19	
>5cm	20	38	
**Distant metastasis**			**0.025**
Yes	7	18	
No	46	39	
**Tumor stage**			**0.002**
I-II	35	20	
III-IV	18	37	
**Intrahepatic metastasis**			**0.001**
Yes	6	22	
No	47	35	
**AFP(μg/L)**			0.566
≤20	26	24	
>20	27	33	
**HBV**			0.073
Positive	30	42	
Negative	23	15	
**Liver Cirrhosis**			0.345

Yes	31	28
No	22	29

Note: HCC patients were divided into WDR6 high group and low group according to the analysis of qRT-PCR. Differences among variables were evaluated by χ^2 or Fisher's exact χ^2 -test.

Appendix Table S5. **Univariate and multivariate analysis of factors associated with survival in HCC patients (n=110)**

Variable	Univariate analysis		multivariate analysis	
	HR (95% CI)	P-value	HR (95% CI)	P-value
**Gender**				
Male vs. Female	0.775(0.466-1.289)	0.326		
**Age**				
>60 vs. ≤60	0.881(0.544-1.428)	0.608		
**Histological differentiation**				
Well/Moderately vs. Poorly	0.683(0.425-1.097)	0.115		
**Tumor size**				
>5cm vs. ≤5cm	0.687(0.428-1.101)	0.119		
**Distant metastasis**				
Yes vs. No	0.417(0.242-0.720)	**0.002**	0.480(0.278-0.831)	**0.009**
**Tumor stage**				
III-IV vs. I-II	0.500(0.301-0.830)	**0.007**		
**AFP (μg/L)**				
≤20 vs. >20	1.704(1.059-2.744)	**0.028**		
**Liver cirrhosis**				
Yes vs. No	1.218(0.758-1.958)	0.414		
**HBV**				
Positive vs. Negative	1.266(0.769-2.085)	0.354		
**WDR6 expression**				

High vs. Low	2.281(1.394-3.734)	**0.001**	1.928(1.157-3.213)	**0.012**
