# Supplementary figures and images for: Targeting WDxR motif reprograms immune microenvironment and inhibits hepatocellular carcinoma progression
Source: EMBO Mol Med. 2023 Mar 22;15(5):e15924. doi: 10.15252/emmm.202215924 (PMC10165360; doi:10.15252/emmm.202215924)

**Ai**

WDR6

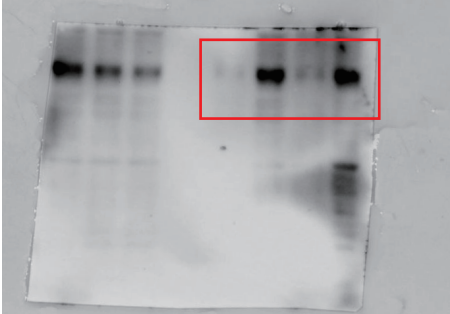

GAPDH

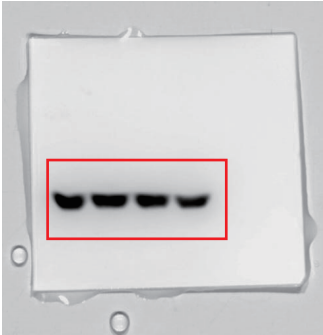

**C**

WDR6

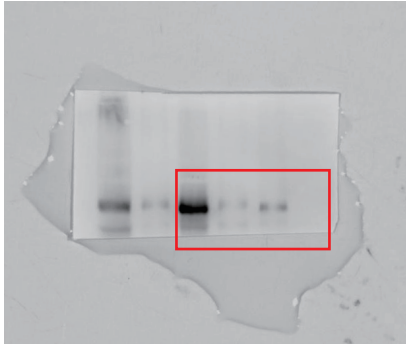

GAPDH

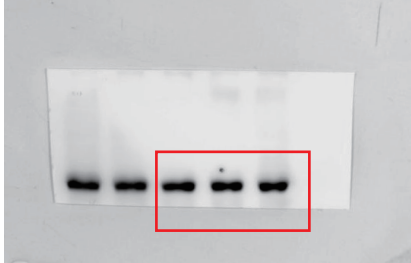

**D**

WDR6

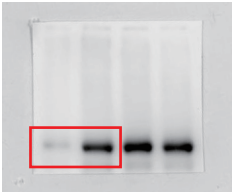

GAPDH

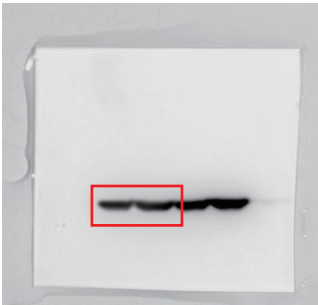

Supplement: Supplementary file 2 — Source Data for Appendix [file EMMM-15-e15924-s004.zip › Supporting S1/FS1A and C and D.pdf]

**Ci**

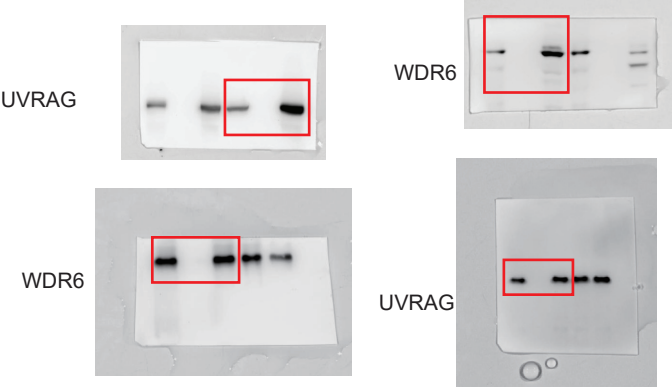

**Cii**

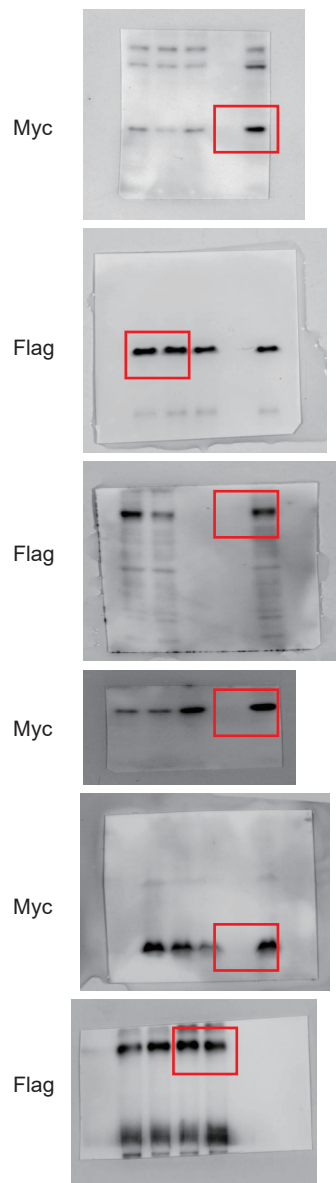

**Ciii**

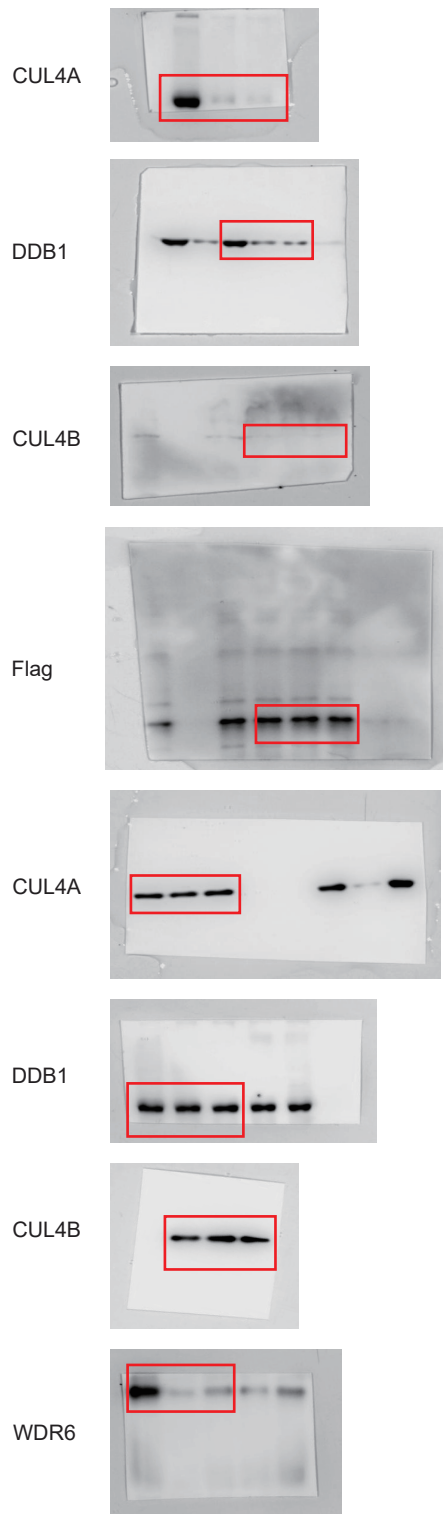

**D**

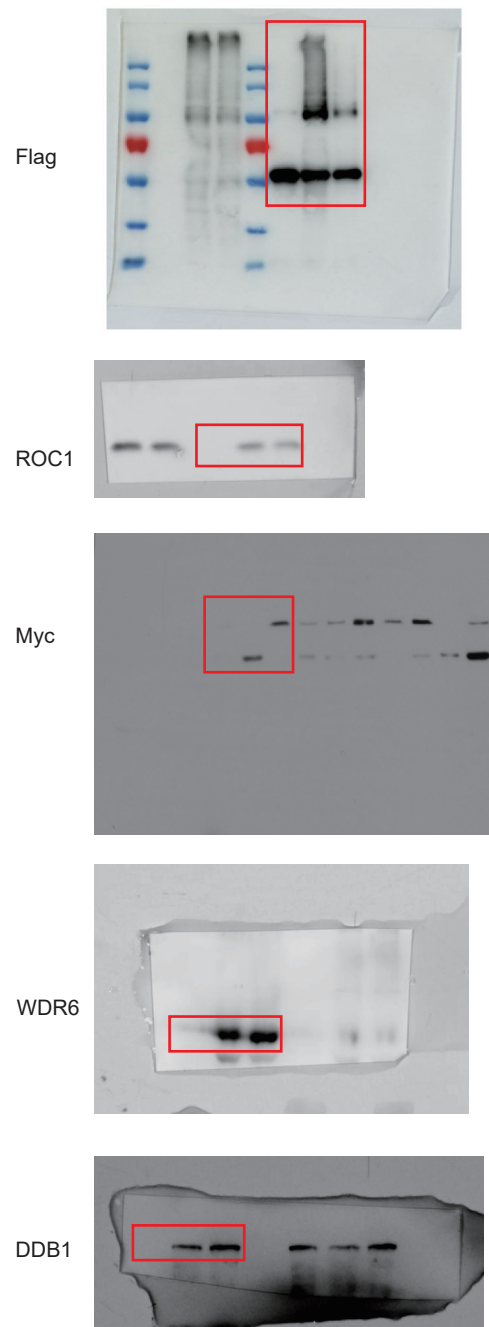

Supplement: Supplementary file 2 — Source Data for Appendix [file EMMM-15-e15924-s004.zip › Supporting S2/FS2.pdf]

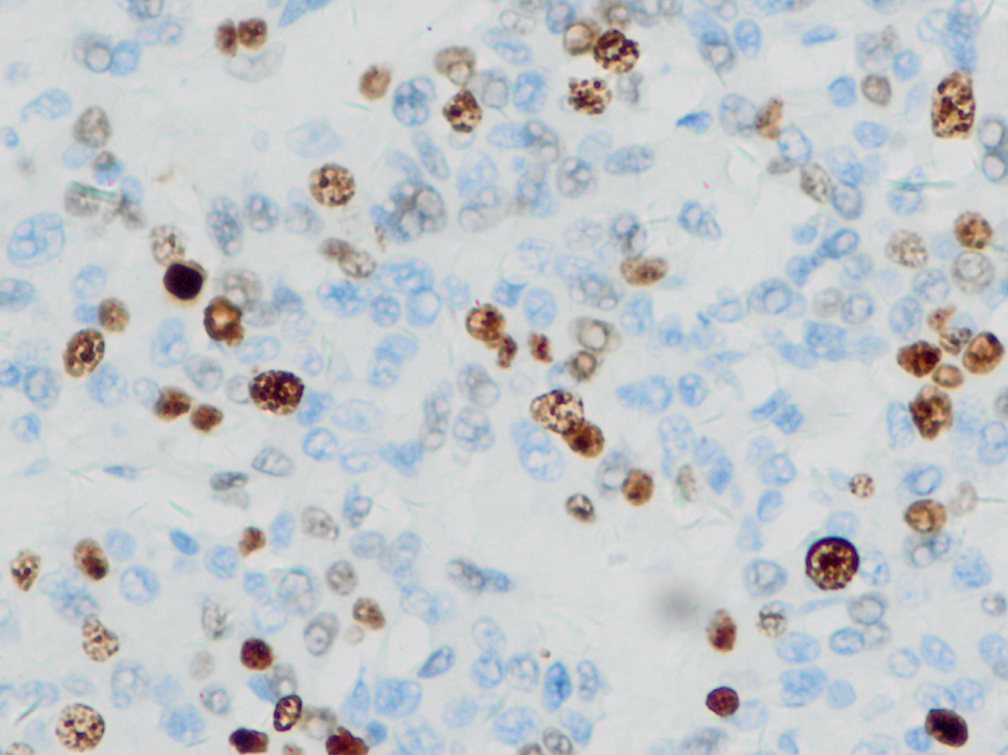

Supplement: Supplementary file 2 — Source Data for Appendix [file EMMM-15-e15924-s004.zip › Supporting S3/case1 pp65.tif]

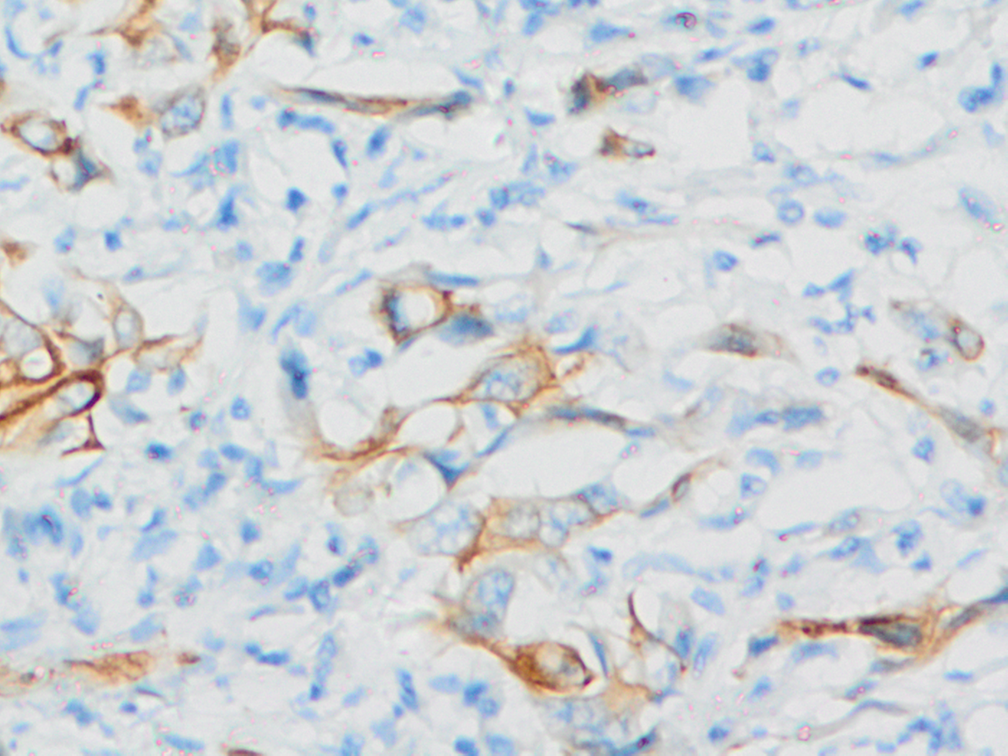

Supplement: Supplementary file 2 — Source Data for Appendix [file EMMM-15-e15924-s004.zip › Supporting S3/case1 UVRAG.tif]

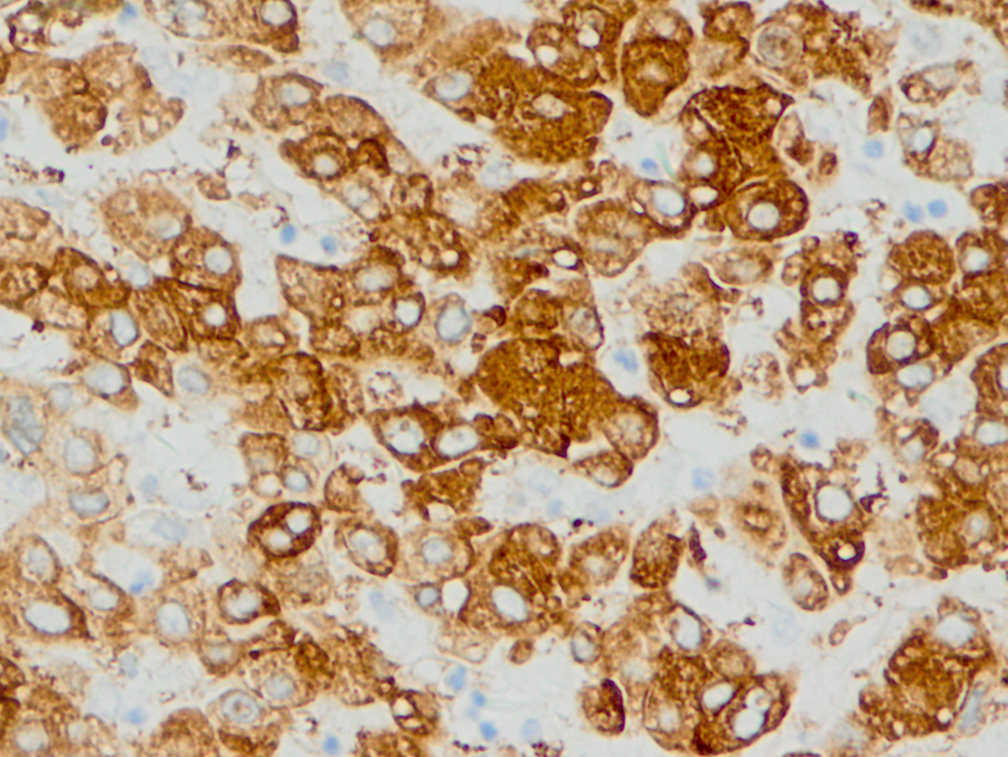

Supplement: Supplementary file 2 — Source Data for Appendix [file EMMM-15-e15924-s004.zip › Supporting S3/case1 WDR6.tif]

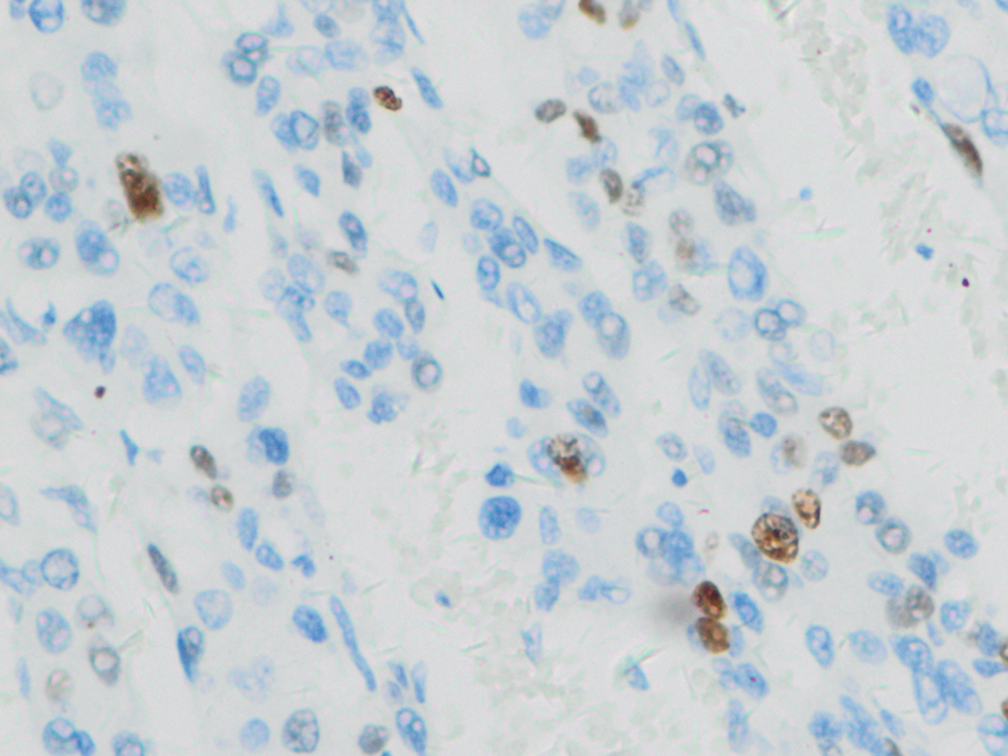

Supplement: Supplementary file 2 — Source Data for Appendix [file EMMM-15-e15924-s004.zip › Supporting S3/case5 pp65.tif]

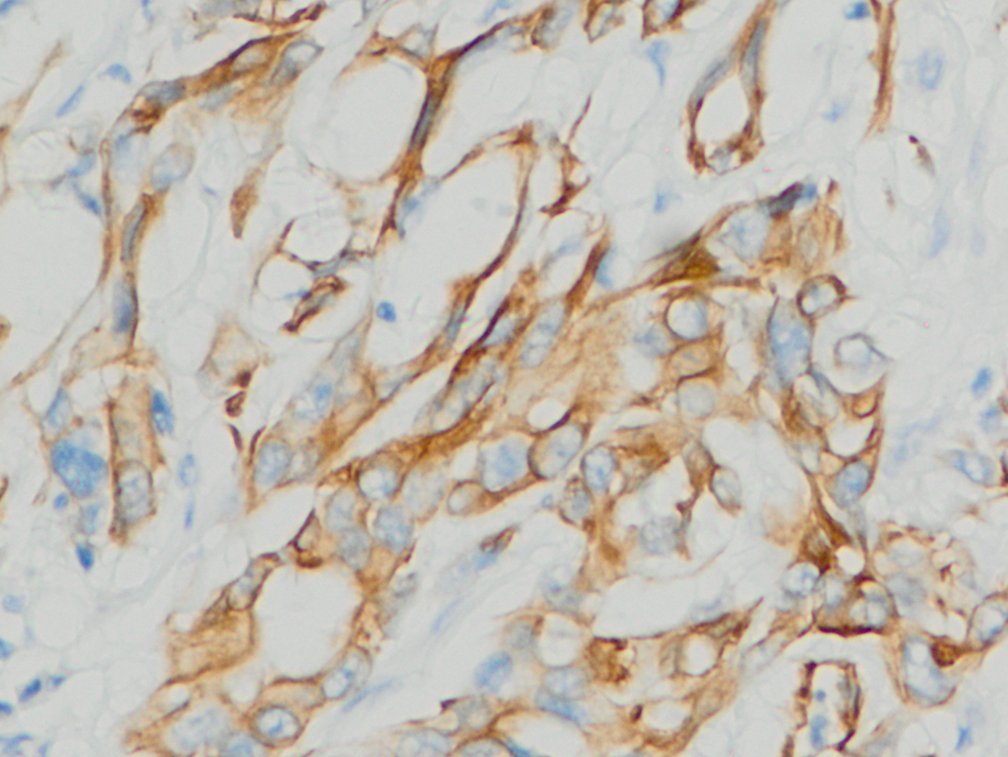

Supplement: Supplementary file 2 — Source Data for Appendix [file EMMM-15-e15924-s004.zip › Supporting S3/case5 UVRAG.tif]

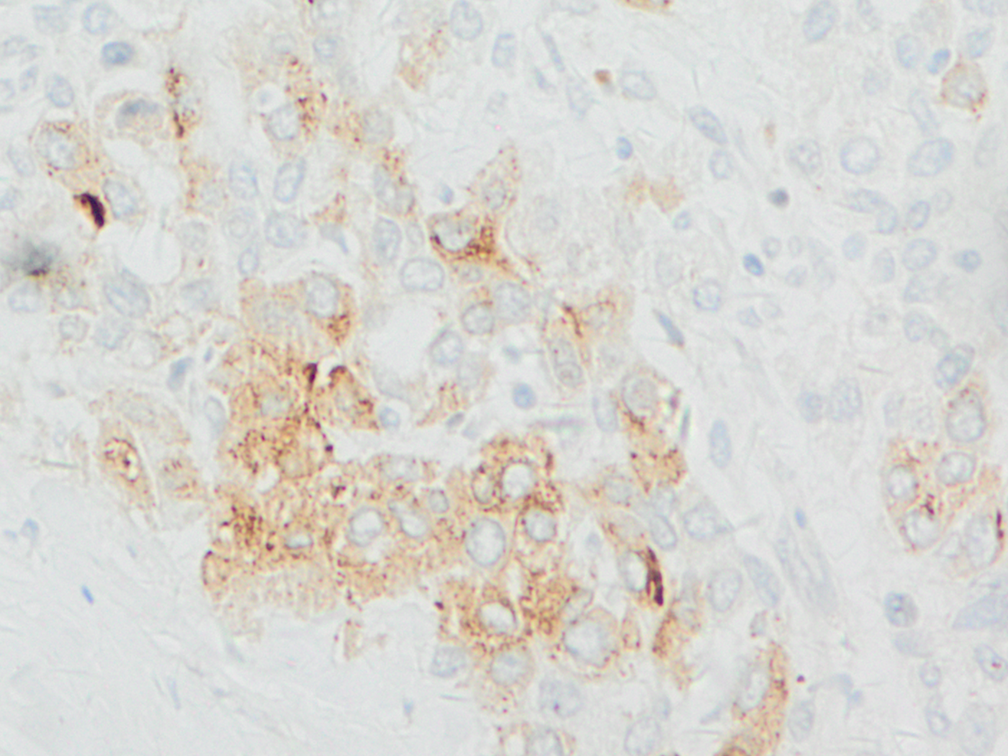

Supplement: Supplementary file 2 — Source Data for Appendix [file EMMM-15-e15924-s004.zip › Supporting S3/case5 WDR6.tif]

**B**

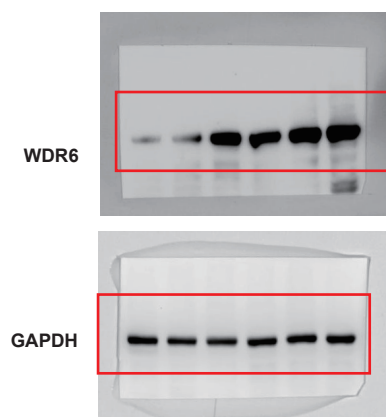

Supplement: Supplementary file 3 — Source Data for Figure 1 [file EMMM-15-e15924-s001.zip › Fig1/Fig1B.pdf]

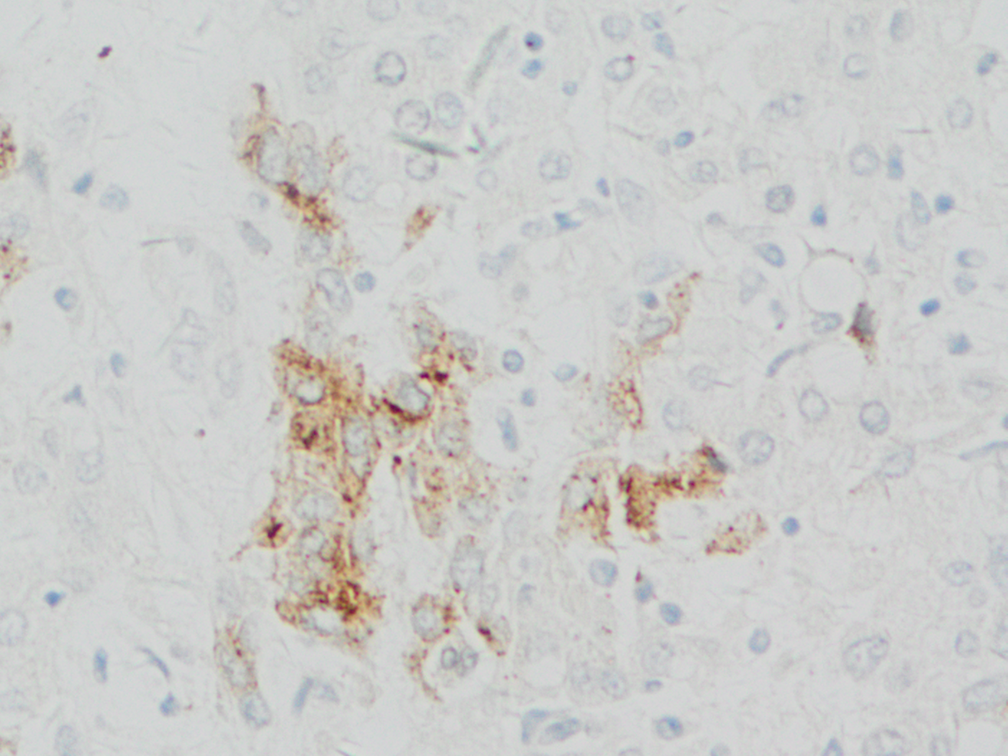

Supplement: Supplementary file 3 — Source Data for Figure 1 [file EMMM-15-e15924-s001.zip › Fig1/Low WDR6.tif]

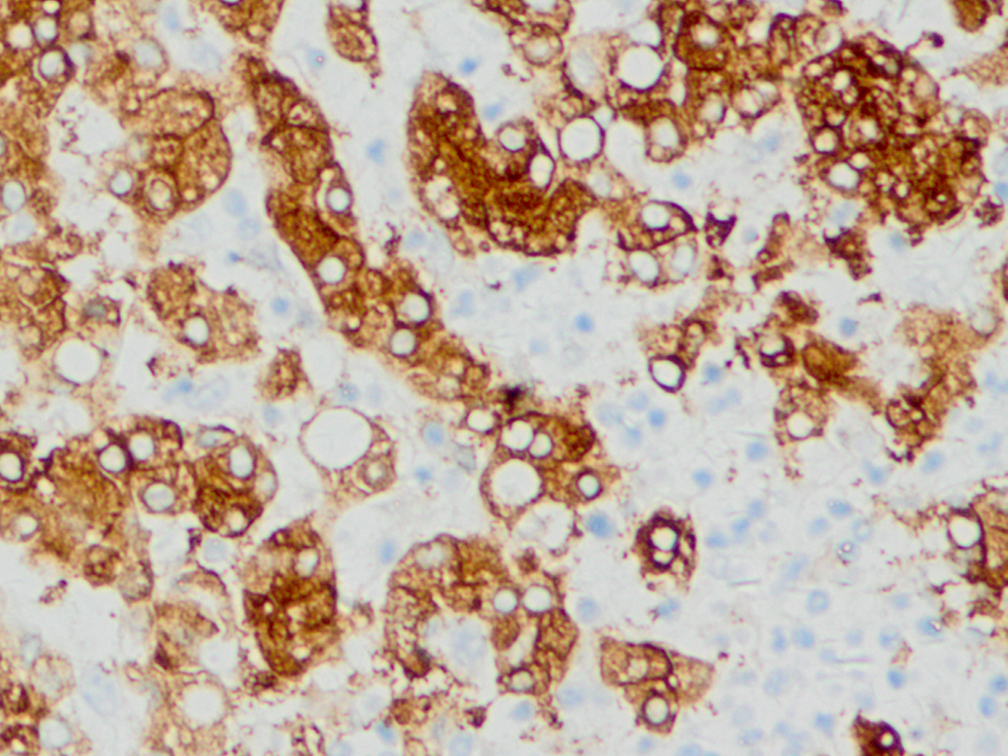

Supplement: Supplementary file 3 — Source Data for Figure 1 [file EMMM-15-e15924-s001.zip › Fig1/High WDR6.tif]

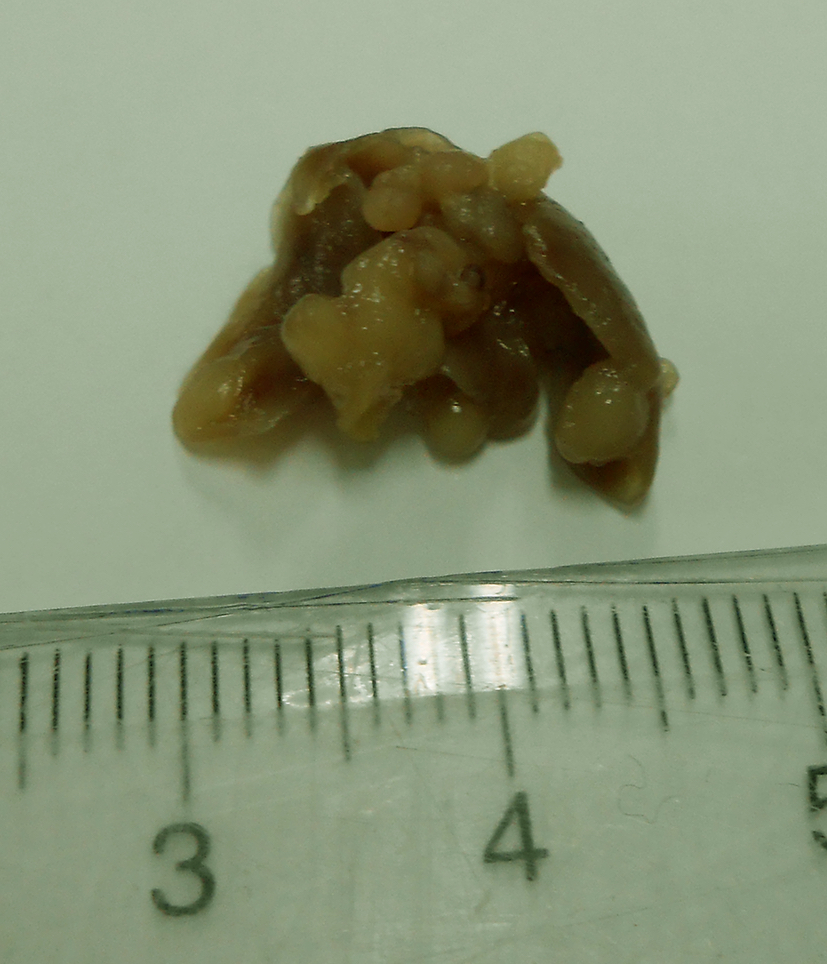

Supplement: Supplementary file 4 — Source Data for Figure 2 [file EMMM-15-e15924-s008.zip › Fig2/shControl lung.jpg]

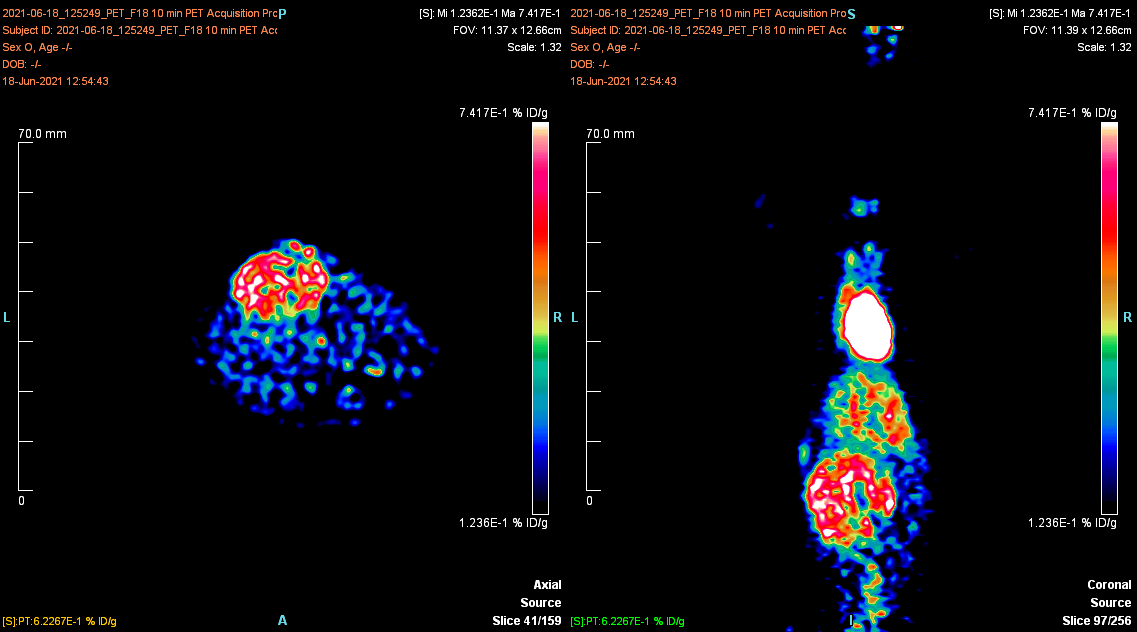

Supplement: Supplementary file 4 — Source Data for Figure 2 [file EMMM-15-e15924-s008.zip › Fig2/shControl liver.tif]

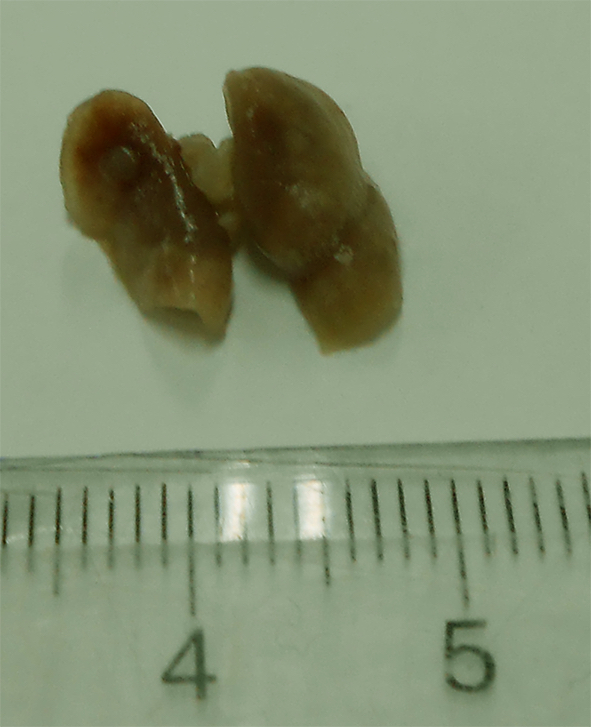

Supplement: Supplementary file 4 — Source Data for Figure 2 [file EMMM-15-e15924-s008.zip › Fig2/shWDR6 lung.jpg]

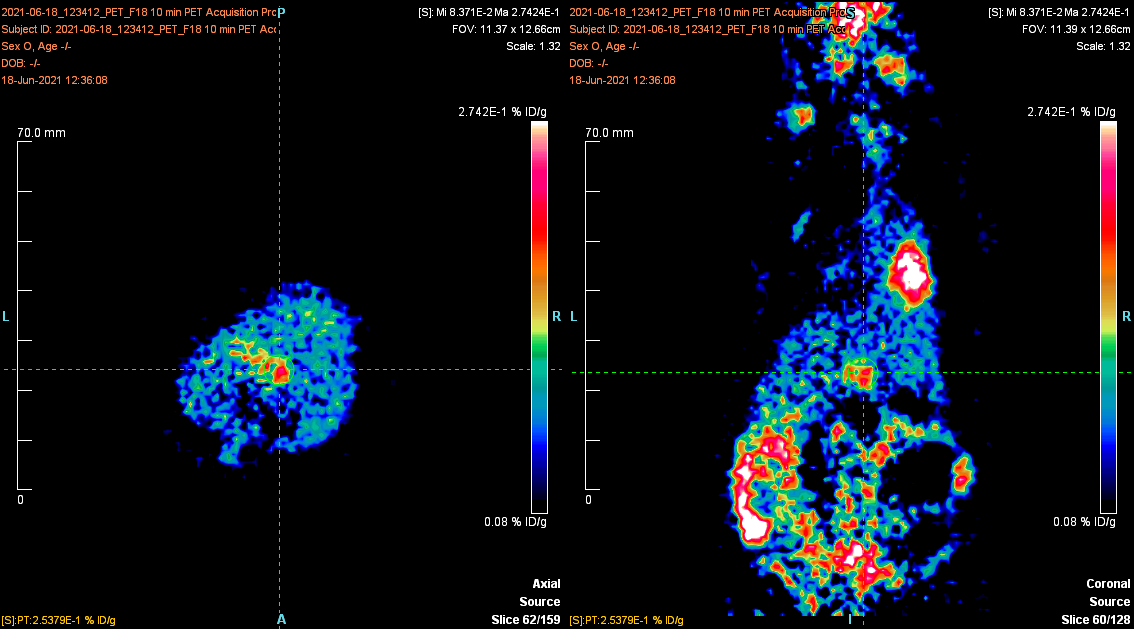

Supplement: Supplementary file 4 — Source Data for Figure 2 [file EMMM-15-e15924-s008.zip › Fig2/shWDR6 liver.tif]

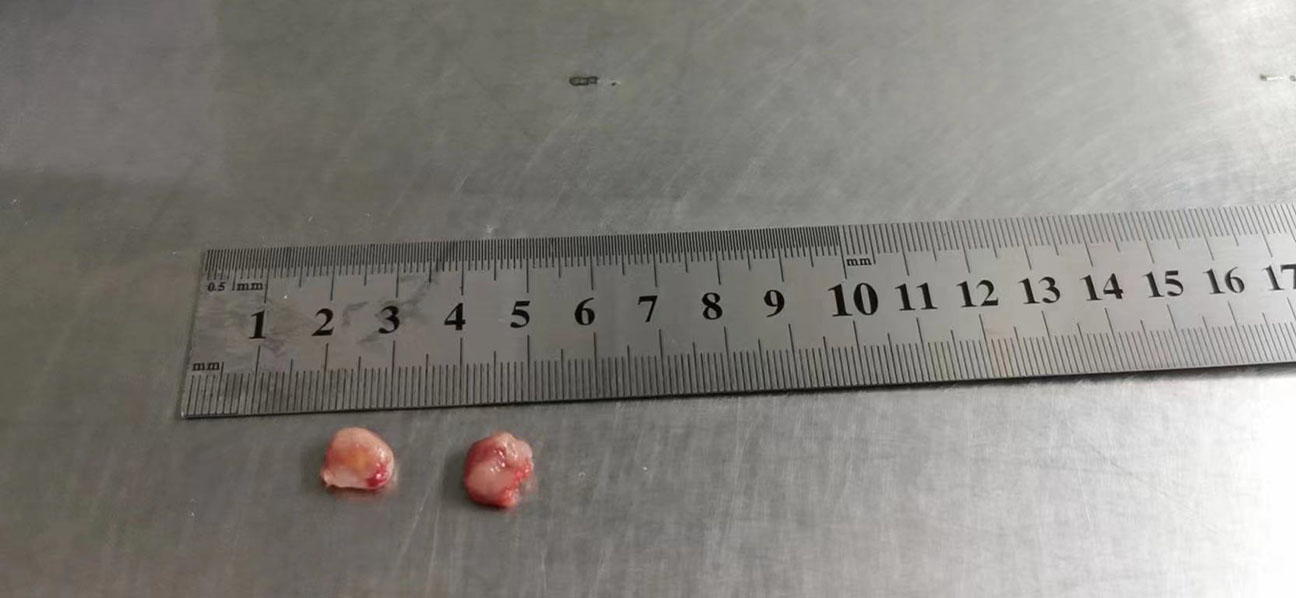

Supplement: Supplementary file 4 — Source Data for Figure 2 [file EMMM-15-e15924-s008.zip › Fig2/Fig2C.jpg]

WDR6

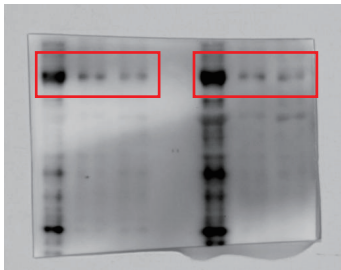

GAPDH

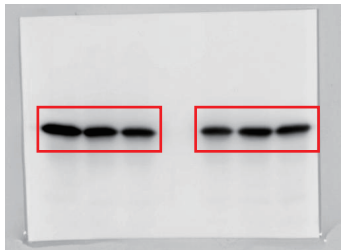

Supplement: Supplementary file 4 — Source Data for Figure 2 [file EMMM-15-e15924-s008.zip › Fig2/Fig2A.pdf]

ci

p65

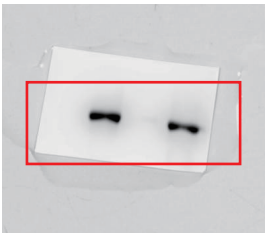

LC3

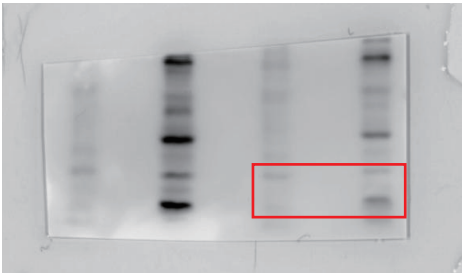

Di

p65

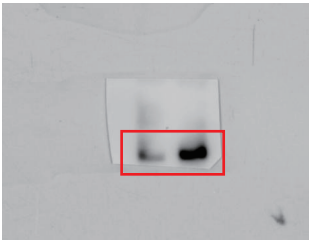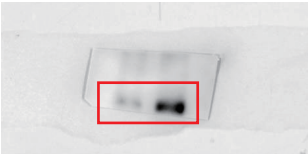

SQSTM1

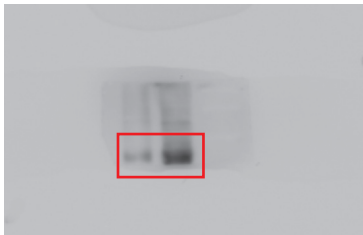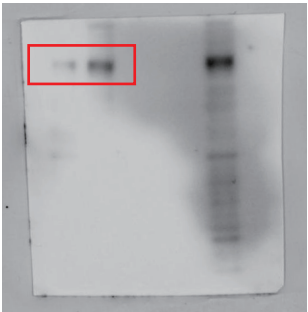

GAPDH

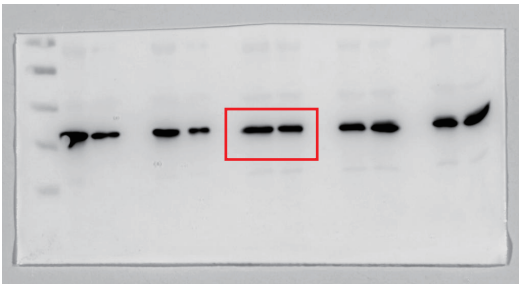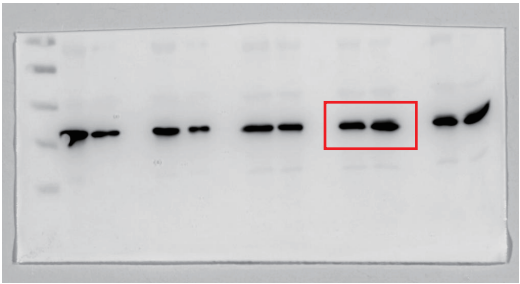

Supplement: Supplementary file 5 — Source Data for Figure 5 [file EMMM-15-e15924-s007.zip › Fig5/Fig5C and D.pdf]

**B**

p65

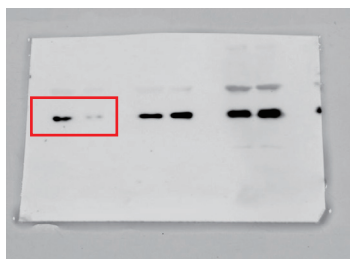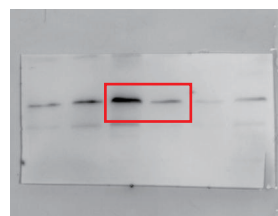

p-p65

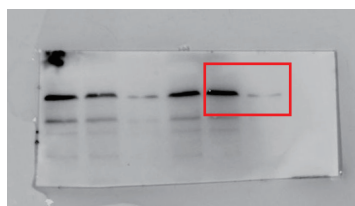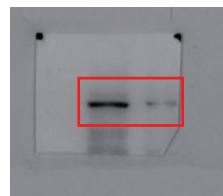

RelB

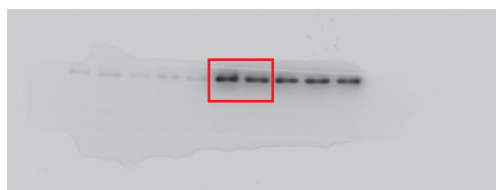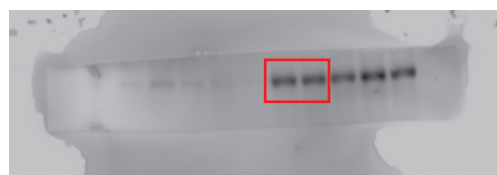

c-Rel

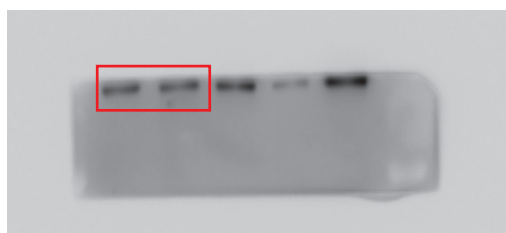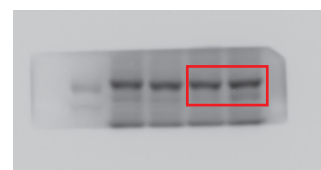

p52

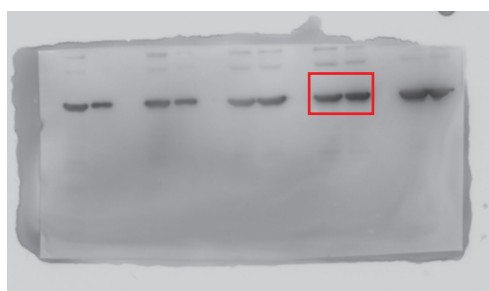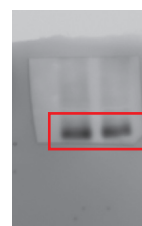

p50

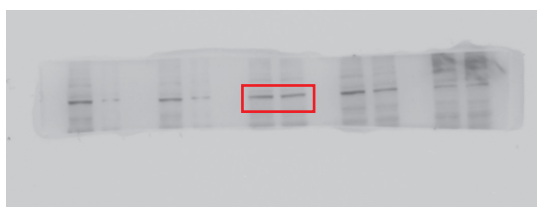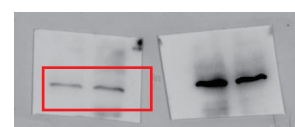

GAPDH

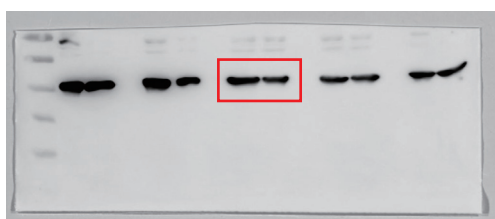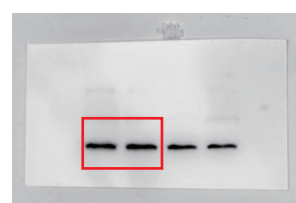

Supplement: Supplementary file 5 — Source Data for Figure 5 [file EMMM-15-e15924-s007.zip › Fig5/Fig5B.pdf]

Ub

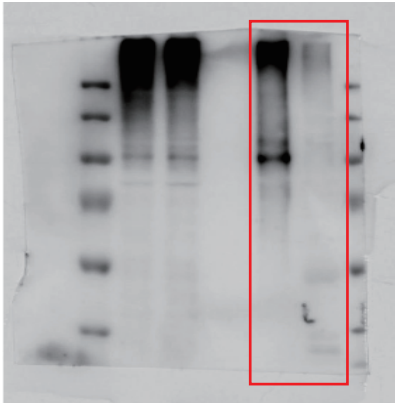

Flag

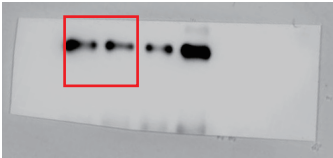

Ub

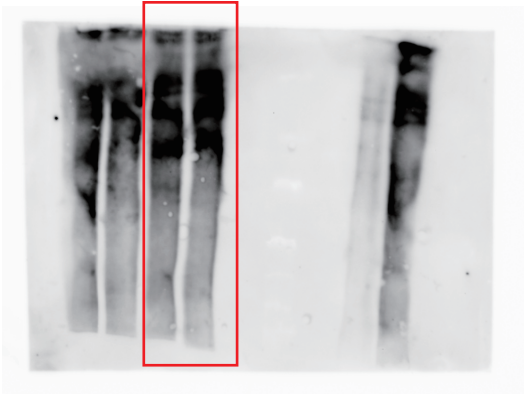

Flag

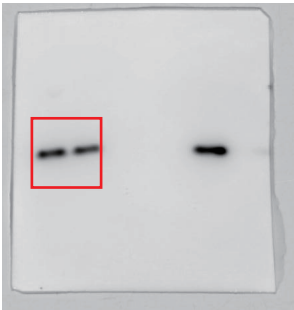

Myc

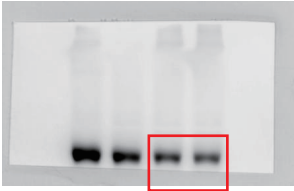

GAPDH

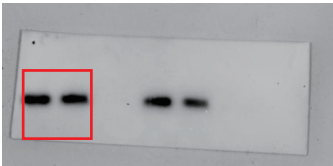

Supplement: Supplementary file 6 — Source Data for Figure 6 [file EMMM-15-e15924-s009.zip › Fig6/Fig6Eii.pdf]

**C**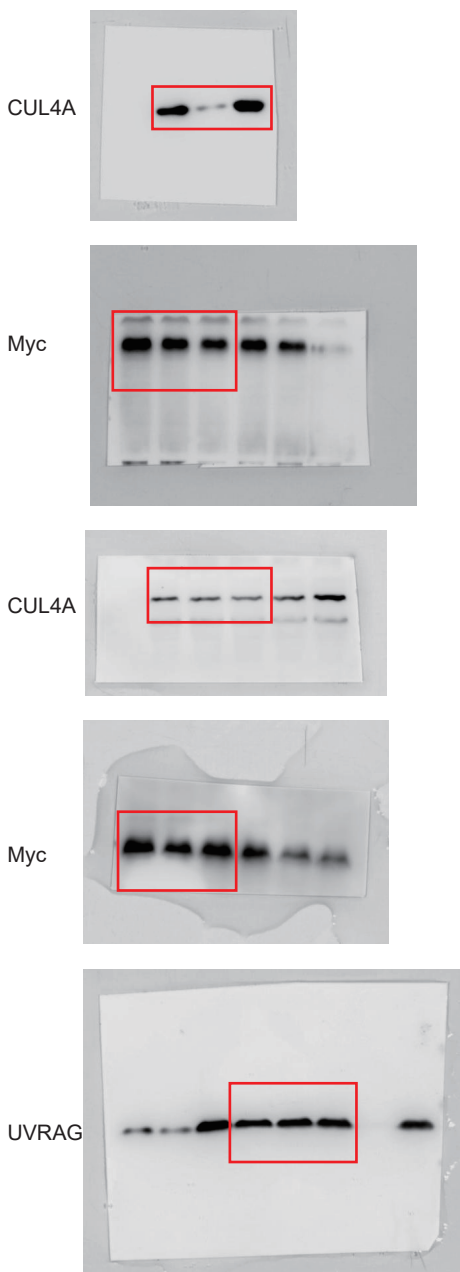**Di**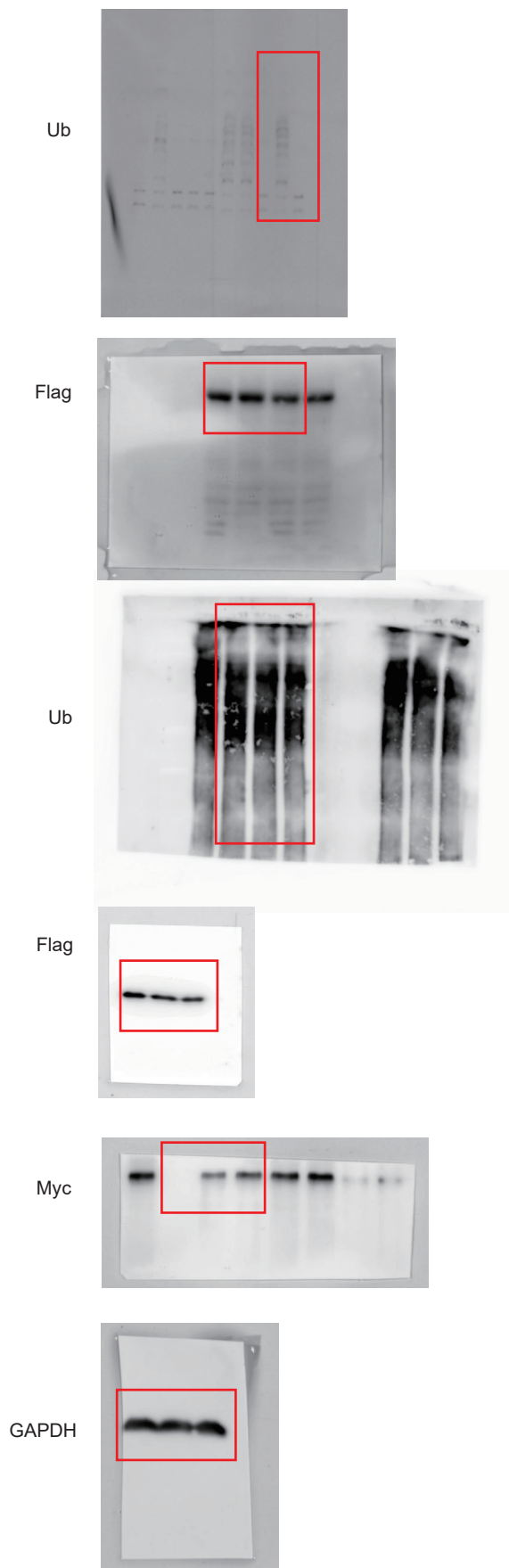**Dii**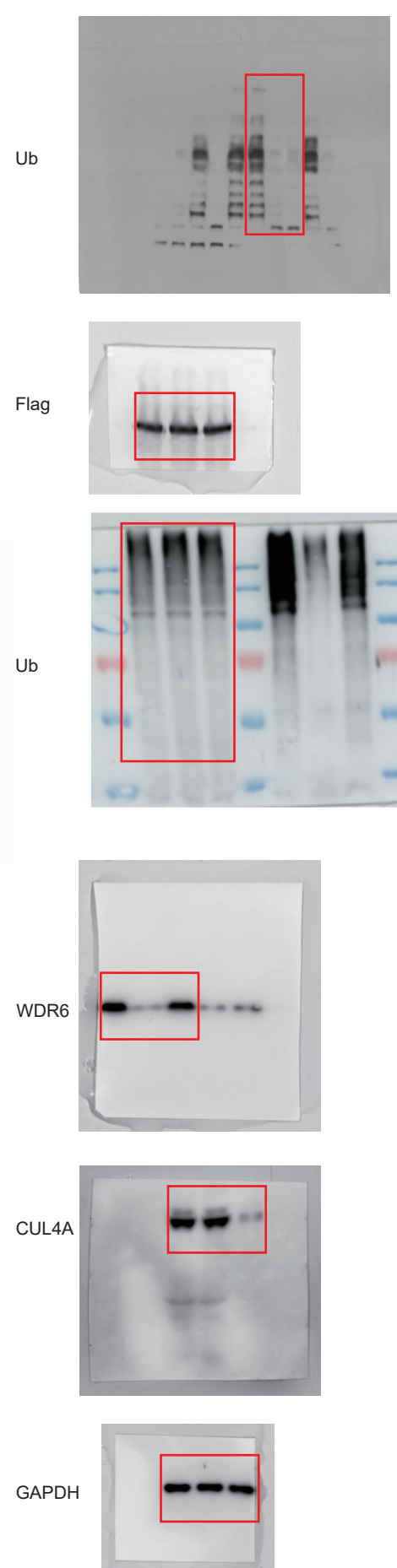

Supplement: Supplementary file 6 — Source Data for Figure 6 [file EMMM-15-e15924-s009.zip › Fig6/Fig6C and D.pdf]

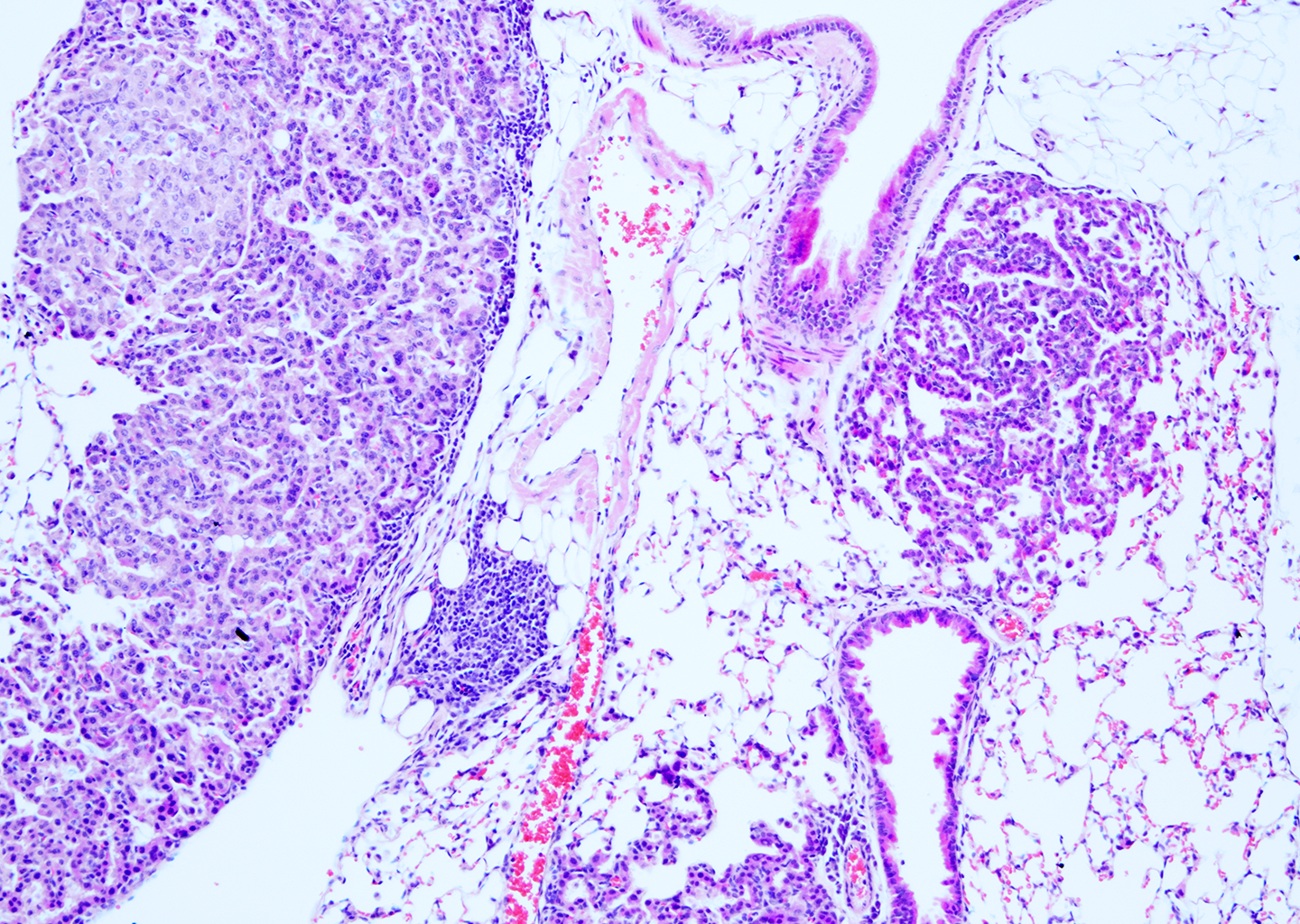

Supplement: Supplementary file 8 — Source Data for Figure 8 [file EMMM-15-e15924-s005.zip › Fig8/pep2 lung.tif]

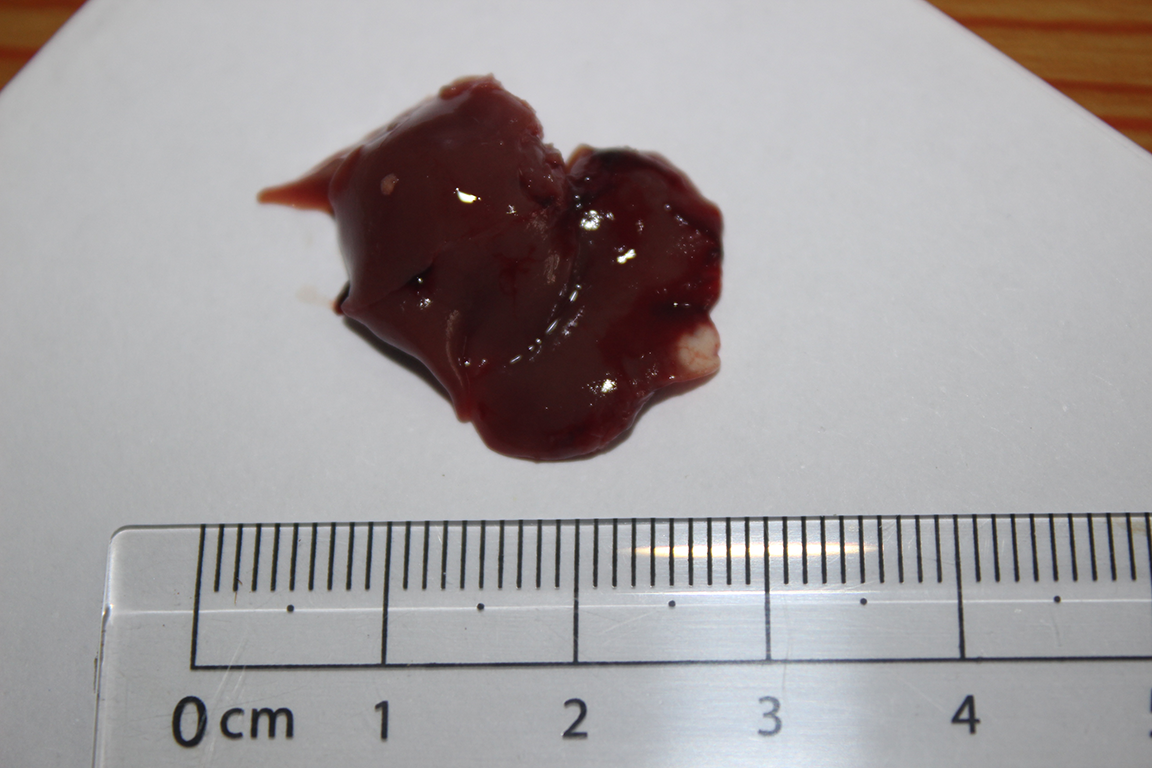

Supplement: Supplementary file 8 — Source Data for Figure 8 [file EMMM-15-e15924-s005.zip › Fig8/pep2 PDL1 liver.tif]

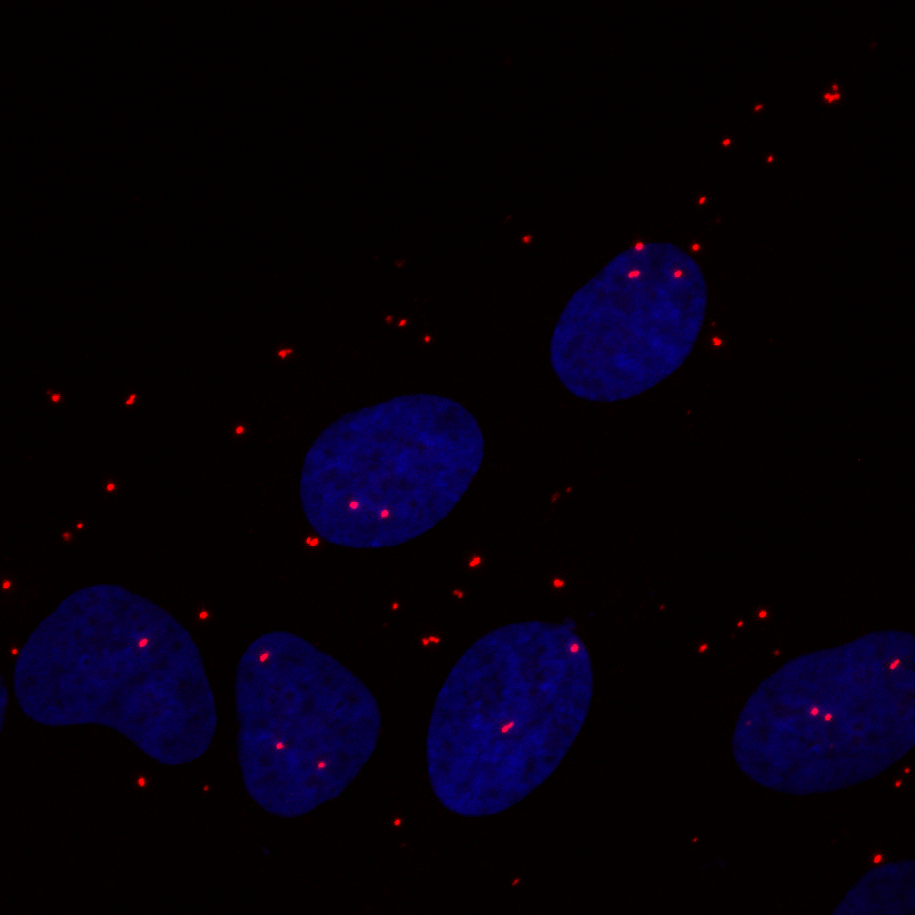

Supplement: Supplementary file 8 — Source Data for Figure 8 [file EMMM-15-e15924-s005.zip › Fig8/pep2 WDxR PLA.tif]

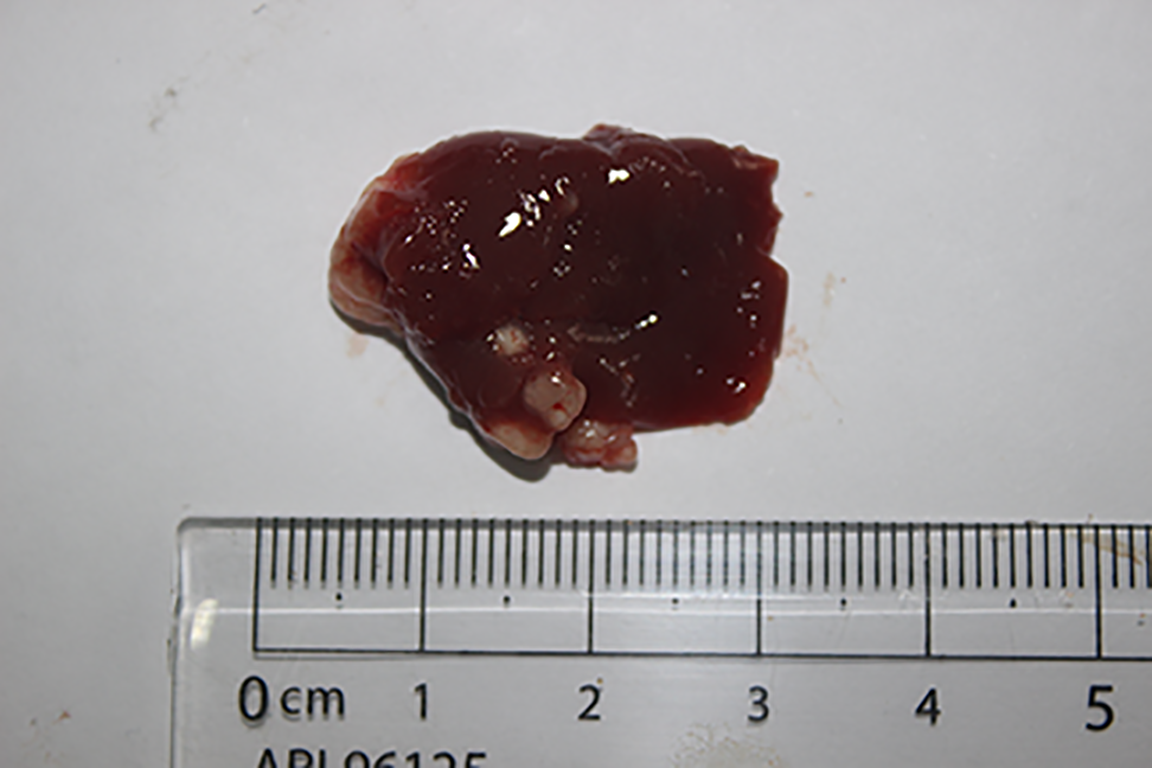

Supplement: Supplementary file 8 — Source Data for Figure 8 [file EMMM-15-e15924-s005.zip › Fig8/pep2 liver.tif]

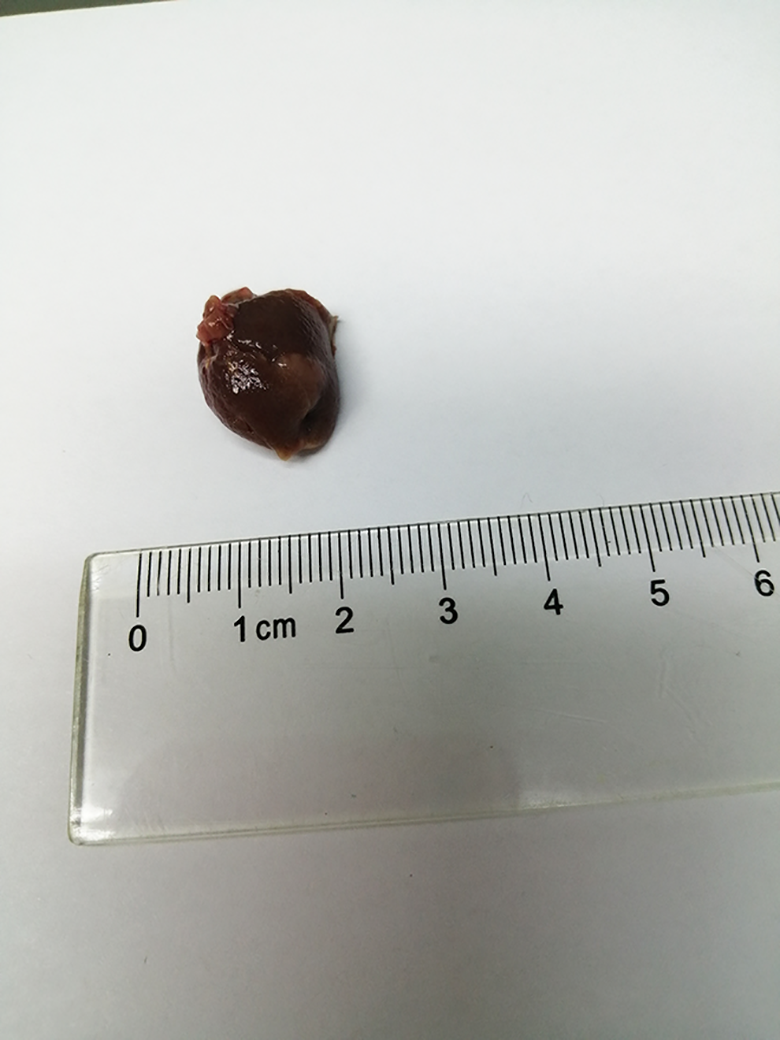

Supplement: Supplementary file 8 — Source Data for Figure 8 [file EMMM-15-e15924-s005.zip › Fig8/PDL1 liver.tif]

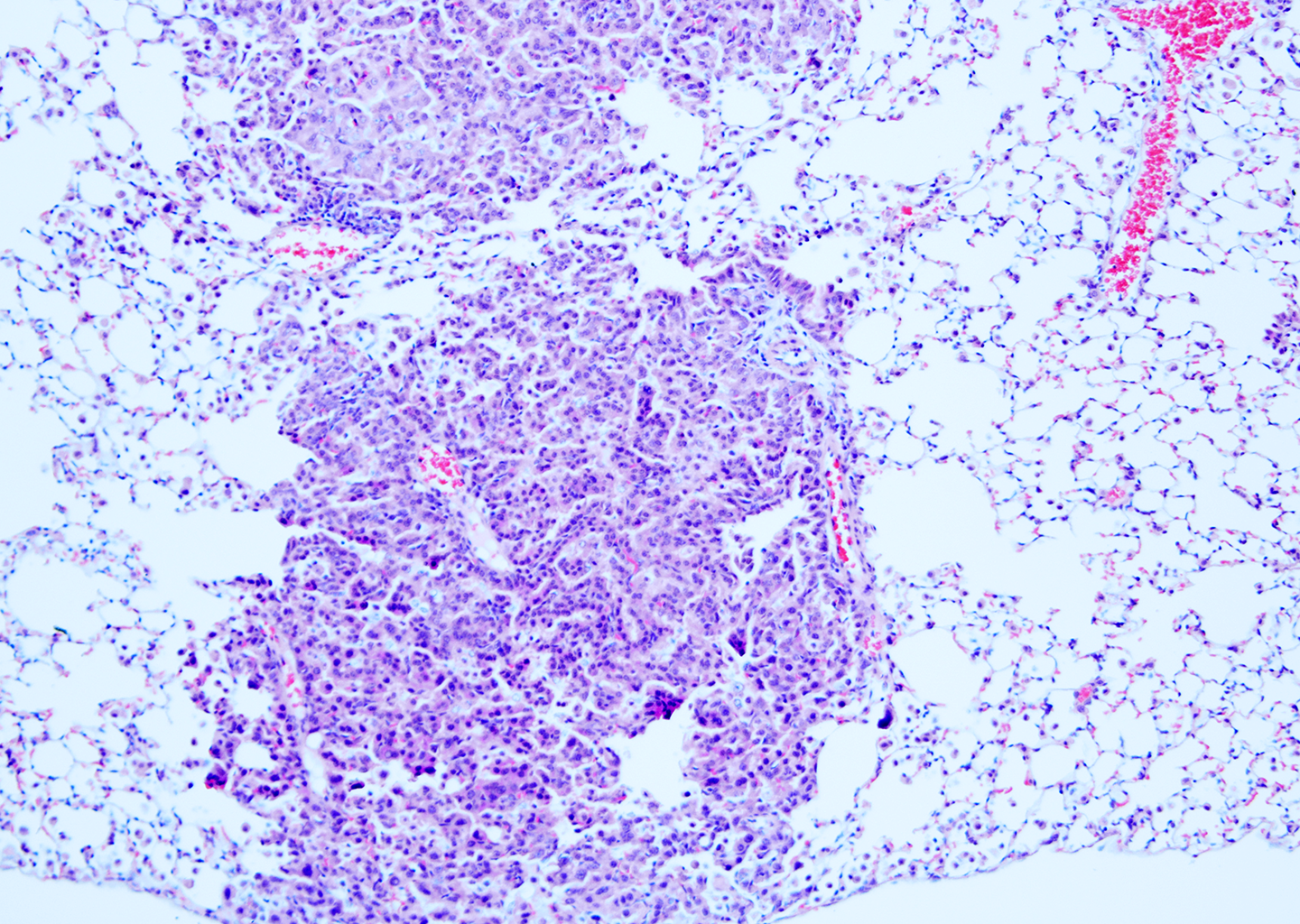

Supplement: Supplementary file 8 — Source Data for Figure 8 [file EMMM-15-e15924-s005.zip › Fig8/vehicle lung.tif]

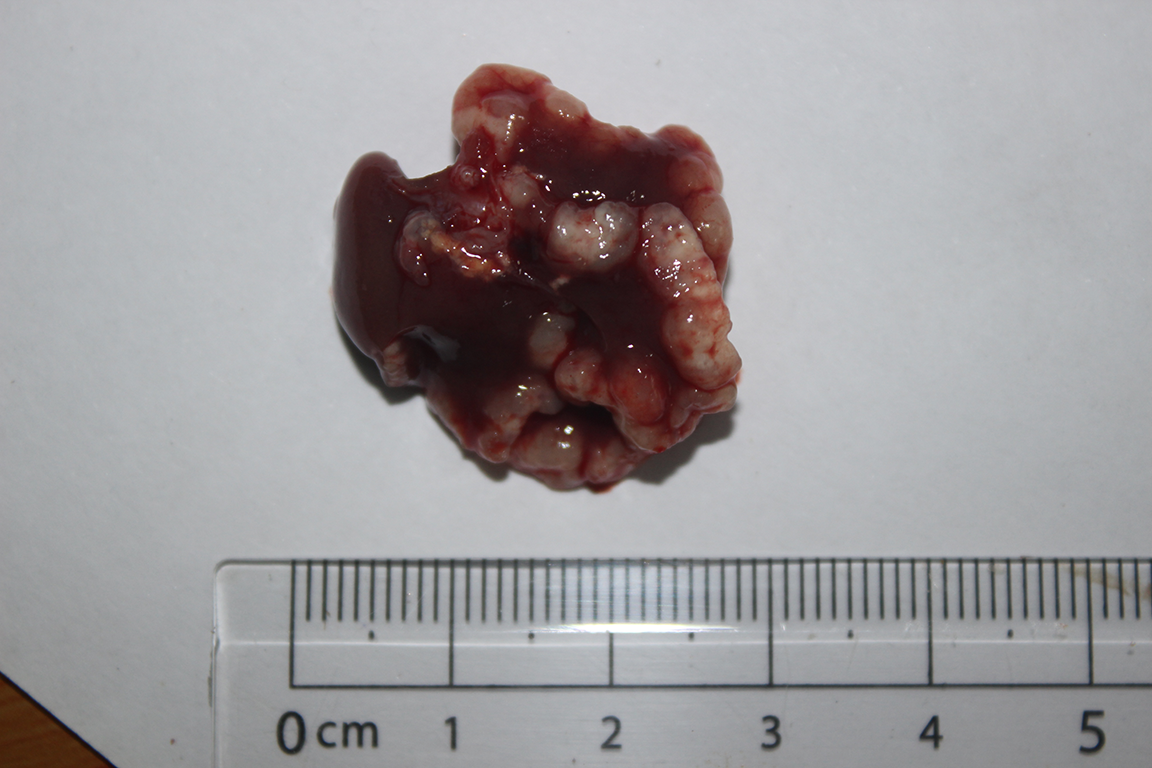

Supplement: Supplementary file 8 — Source Data for Figure 8 [file EMMM-15-e15924-s005.zip › Fig8/Vehicle liver.tif]

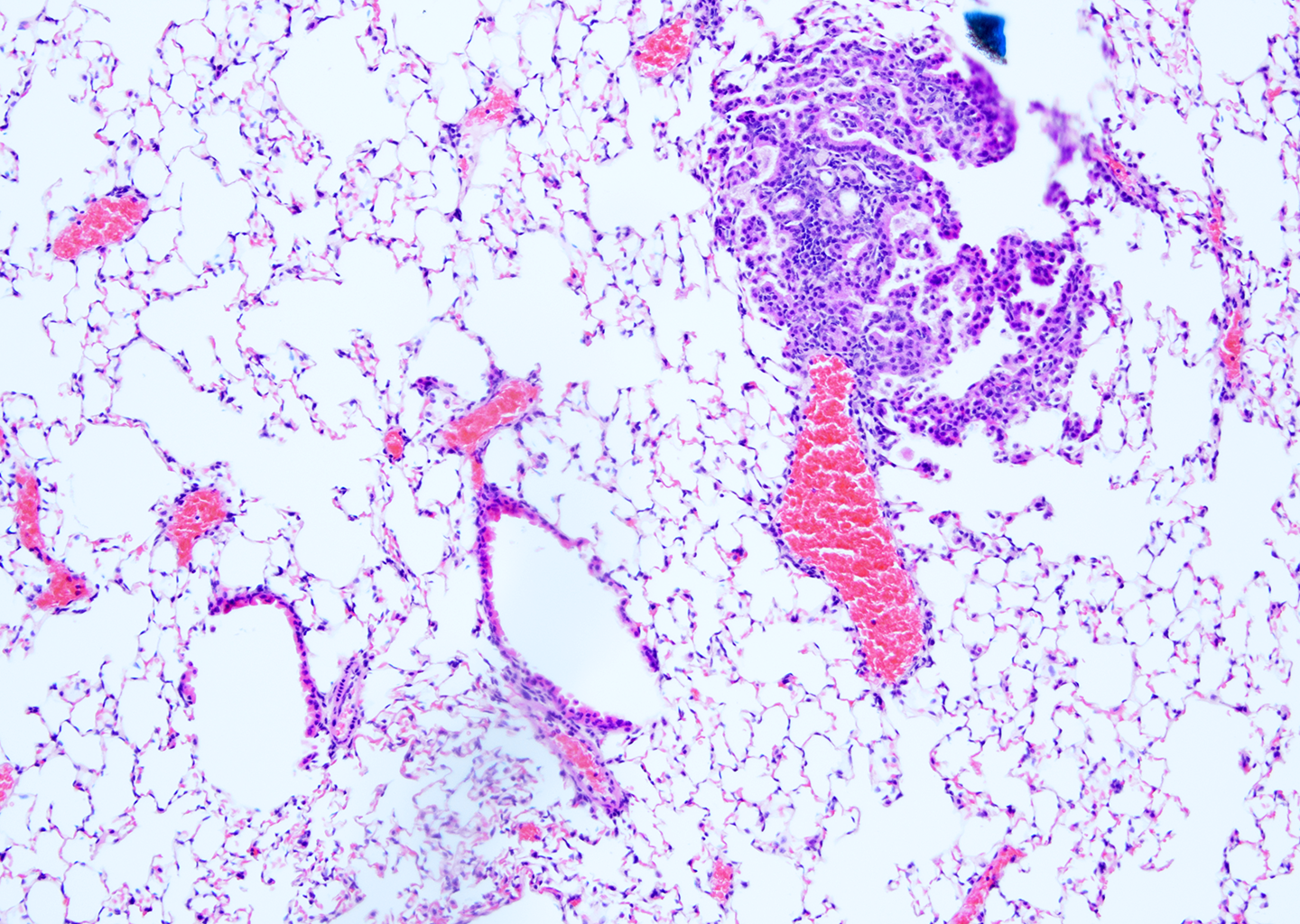

Supplement: Supplementary file 8 — Source Data for Figure 8 [file EMMM-15-e15924-s005.zip › Fig8/PDL1 lung.tif]

**A**

HA

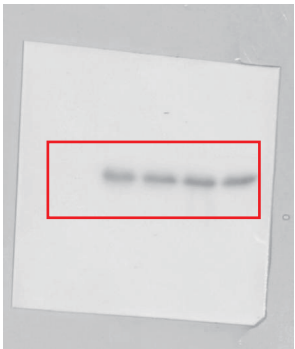

His

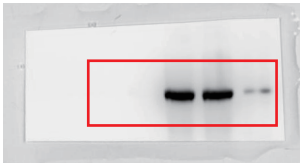

HA

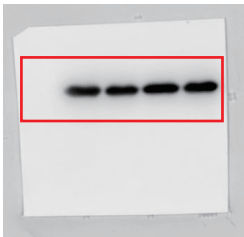

His

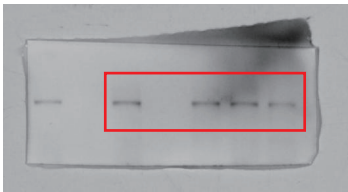

Supplement: Supplementary file 8 — Source Data for Figure 8 [file EMMM-15-e15924-s005.zip › Fig8/Fig8A.pdf]

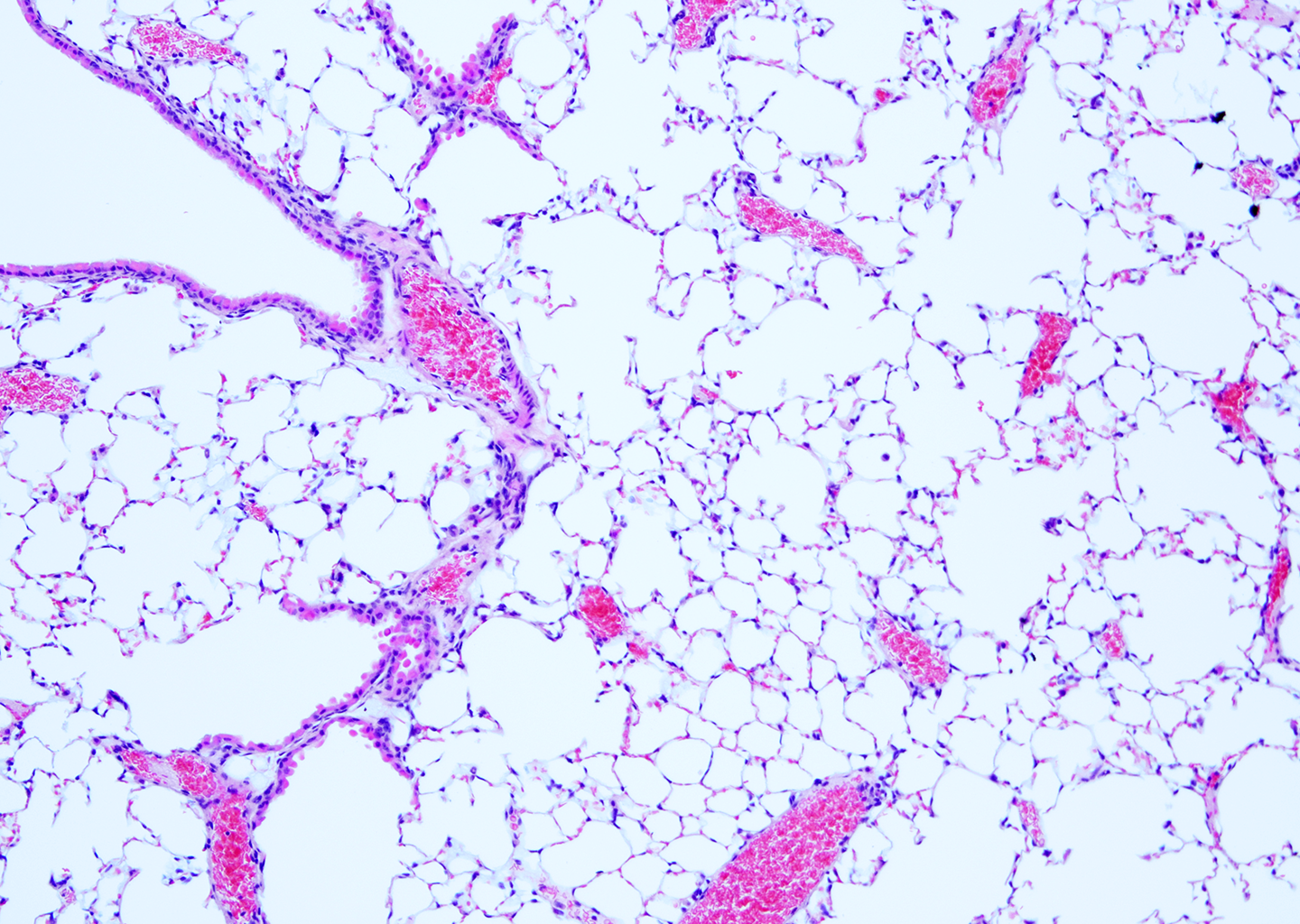

Supplement: Supplementary file 8 — Source Data for Figure 8 [file EMMM-15-e15924-s005.zip › Fig8/pep2 PDL1 lung.tif]

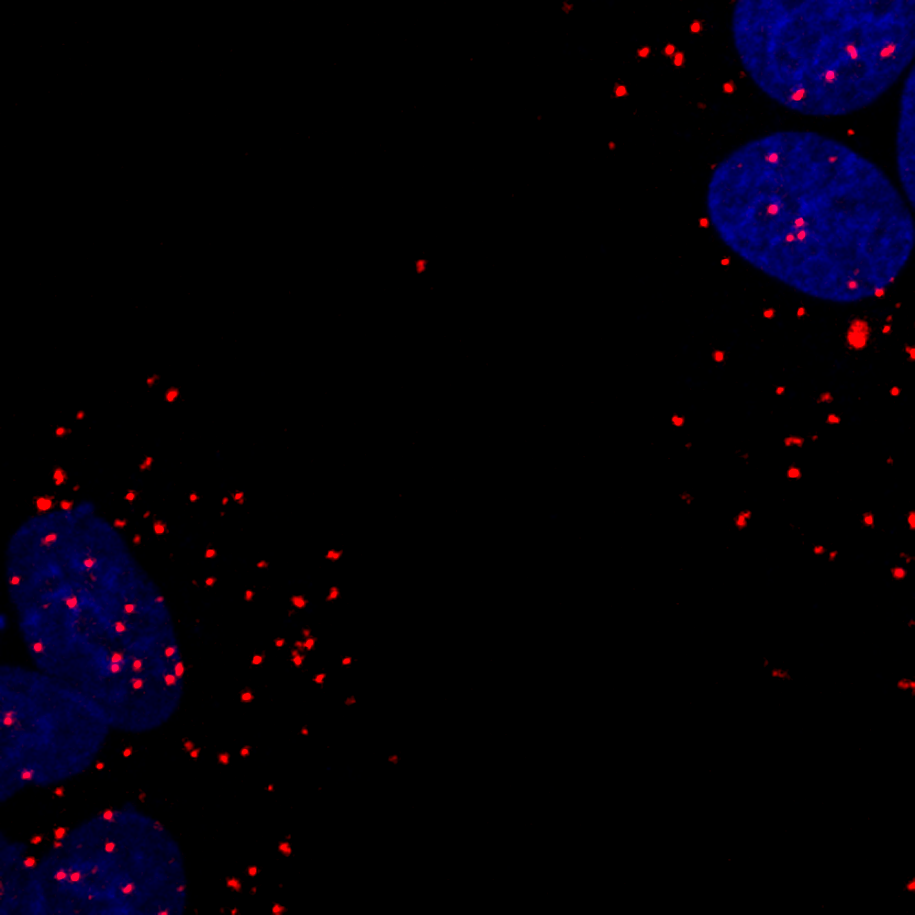

Supplement: Supplementary file 8 — Source Data for Figure 8 [file EMMM-15-e15924-s005.zip › Fig8/pep2 control PLA.tif]
