# Supplementary material for: Targeting WDxR motif reprograms immune microenvironment and inhibits hepatocellular carcinoma progression
Source: EMBO Mol Med. 2023 Mar 22;15(5):e15924. doi: 10.15252/emmm.202215924 (PMC10165360; doi:10.15252/emmm.202215924)
Supplement: Supplementary file 7 — Source Data for Figure 7 [file EMMM-15-e15924-s002.zip › Fig7/Fig7.pdf]

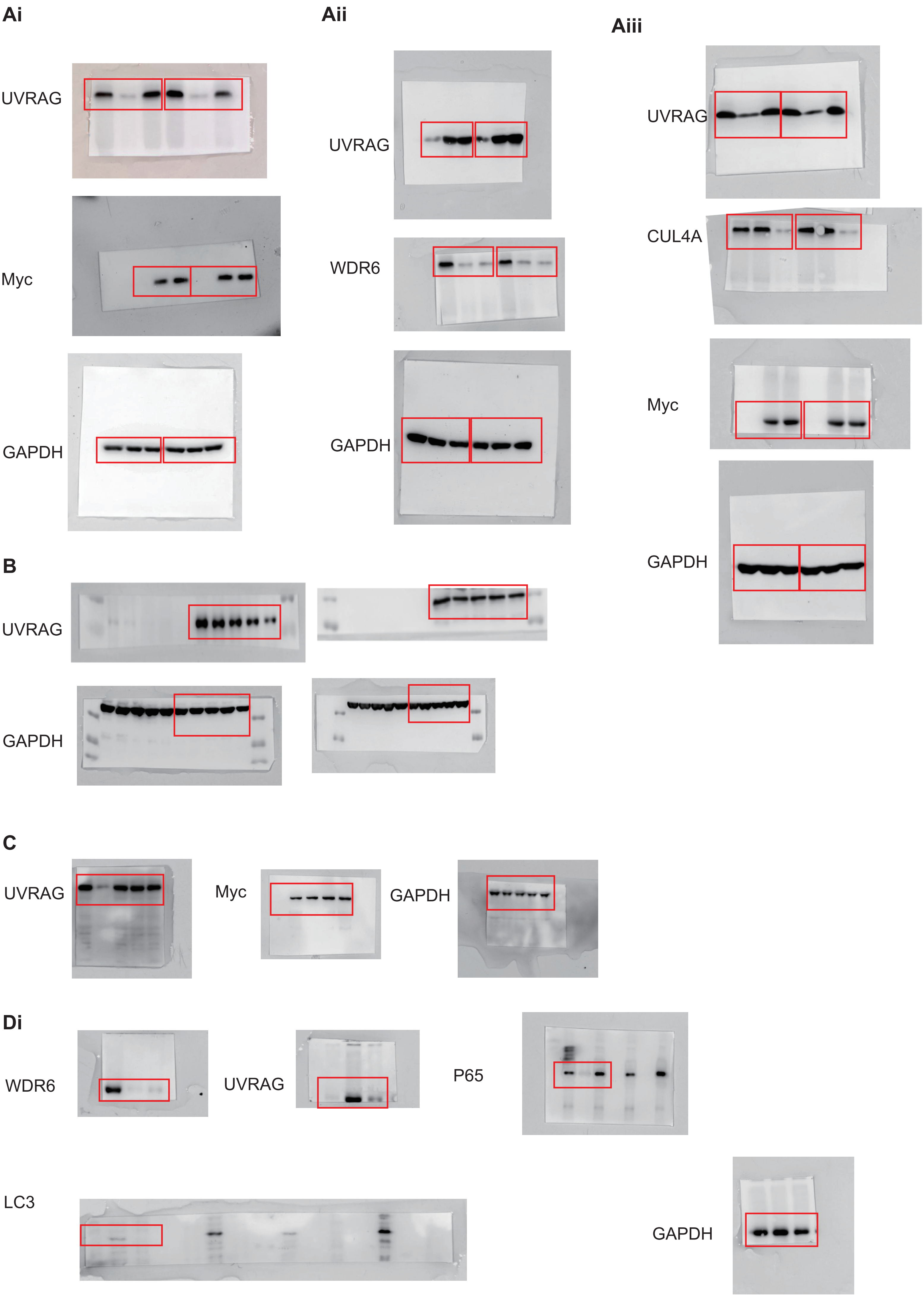
Ai
UVRAG
Myc
GAPDH
Aii
UVRAG
WDR6
GAPDH
Aiii
UVRAG
CUL4A
Myc
GAPDH
B
UVRAG
GAPDH
C
UVRAG
Myc
GAPDH
Di
WDR6
UVRAG
P65
LC3
GAPDH
