# Supplementary material for: Targeting WDxR motif reprograms immune microenvironment and inhibits hepatocellular carcinoma progression
Source: EMBO Mol Med. 2023 Mar 22;15(5):e15924. doi: 10.15252/emmm.202215924 (PMC10165360; doi:10.15252/emmm.202215924)
Supplement: Supplementary file 9 [file EMMM-15-e15924-s006.pdf]

EMBO Molecular Medicine

Targeting WDxR motif reprograms immune microenvironment and inhibits hepatocellular carcinoma progression

Heng Zhang, Gang Chen, Xing Feng, Huiwen Song, Lingbing Meng, Yao Fu, Jun Yang, Zhiwen Fan, Youxiang Ding, Zhijie Du, Jianchao Wang, Li Yang, Jun Zhang, Lixia Sun, Zhigang Liu, zhiyong zhang, quanhai li, and Xiangshan Fan
DOI: 10.15252/emmm.202215924

Corresponding author(s): zhiyong zhang (zhangz2@rwjms.rutgers.edu) , quanhai li (liquanh2@163.com), Xiangshan Fan (fanxiangshan@nju.edu.cn)

Review Timeline:

Submission Date:	20th Feb 22
Editorial Decision:	8th Mar 22
Revision Received:	30th Jun 22
Editorial Decision:	15th Jul 22
Revision Received:	11th Dec 22
Editorial Decision:	12th Jan 23
Revision Received:	25th Feb 23
Accepted:	28th Feb 23

Editor: Lise Roth

Transaction Report:

(Note: With the exception of the correction of typographical or spelling errors that could be a source of ambiguity, letters and reports are not edited. Depending on transfer agreements, referee reports obtained elsewhere may or may not be included in this compilation. Referee reports are anonymous unless the Referee chooses to sign their reports.)

8th Mar 2022

Dear Prof. Zhang,

Thank you for the submission of your manuscript to EMBO Molecular Medicine. We have now received feedback from the three reviewers who agreed to evaluate your manuscript. As you will see from the reports below, the referees acknowledge the interest of the study and are overall supporting publication of your work pending appropriate revisions.

Addressing the reviewers' concerns in full will be necessary for further considering the manuscript in our journal, and acceptance of the manuscript will entail a second round of review. Please note that the referees insisted on having the manuscript edited for English language. EMBO Molecular Medicine encourages a single round of revision only and therefore, acceptance or rejection of the manuscript will depend on the completeness of your responses included in the next, final version of the manuscript. For this reason, and to save you from any frustrations in the end, I would strongly advise against returning an incomplete revision.

Revised manuscripts should be submitted within three months of a request for revision; they will otherwise be treated as new submissions, except under exceptional circumstances in which a short extension is obtained from the editor.

When submitting your revised manuscript, please carefully review the instructions that follow below. We perform an initial quality control of all revised manuscripts before re-review; failure to include requested items will delay the evaluation of your revision.

We require:

1) A .docx formatted version of the manuscript text (including legends for main figures, EV figures and tables). Please make sure that the changes are highlighted to be clearly visible.

2) Individual production quality figure files as .eps, .tif, .jpg (one file per figure). For guidance, download the 'Figure Guide PDF' (https://www.embopress.org/page/journal/17574684/authorguide#figureformat).

3) A .docx formatted letter INCLUDING the reviewers' reports and your detailed point-by-point responses to their comments. As part of the EMBO Press transparent editorial process, the point-by-point response is part of the Review Process File (RPF), which will be published alongside your paper.

4) A complete author checklist, which you can download from our author guidelines (https://www.embopress.org/page/journal/17574684/authorguide#submissionofrevisions). Please insert information in the checklist that is also reflected in the manuscript. The completed author checklist will also be part of the RPF.

5) It is mandatory to include a 'Data Availability' section after the Materials and Methods. Before submitting your revision, primary datasets produced in this study need to be deposited in an appropriate public database, and the accession numbers and database listed under 'Data Availability'. Please remember to provide a reviewer password if the datasets are not yet public (see https://www.embopress.org/page/journal/17574684/authorguide#dataavailability).
In case you have no data that requires deposition in a public database, please state so in this section. Note that the Data Availability Section is restricted to new primary data that are part of this study.

6) For data quantification: please specify the name of the statistical test used to generate error bars and P values, the number (n) of independent experiments (specify technical or biological replicates) underlying each data point and the test used to calculate p-values in each figure legend. The figure legends should contain a basic description of n, P and the test applied. Exact p values must be included in figures or legends. Graphs must include a description of the bars and the error bars (s.d., s.e.m.).

7) We would also encourage you to include the source data for figure panels that show essential data. Numerical data should be provided as individual .xls or .csv files (including a tab describing the data). For blots or microscopy, uncropped images should be submitted (using a zip archive if multiple images need to be supplied for one panel). Additional information on source data and instruction on how to label the files are available at
.

8) Our journal encourages inclusion of *data citations in the reference list* to directly cite datasets that were re-used and obtained from public databases. Data citations in the article text are distinct from normal bibliographical citations and should directly link to the database records from which the data can be accessed. In the main text, data citations are formatted as follows: "Data ref: Smith et al, 2001" or "Data ref: NCBI Sequence Read Archive PRJNA342805, 2017". In the Reference list, data citations must be labeled with "[DATASET]". A data reference must provide the database name, accession number/identifiers and a resolvable link to the landing page from which the data can be accessed at the end of the reference. Further instructions are available at .

9) We replaced Supplementary Information with Expanded View (EV) Figures and Tables that are collapsible/expandable online. A maximum of 5 EV Figures can be typeset. EV Figures should be cited as 'Figure EV1, Figure EV2" etc... in the text and their respective legends should be included in the main text after the legends of regular figures.
- For the figures that you do NOT wish to display as Expanded View figures, they should be bundled together with their legends in a single PDF file called *Appendix*, which should start with a short Table of Content. Appendix figures should be referred to in the main text as: "Appendix Figure S1, Appendix Figure S2" etc.
- Additional Tables/Datasets should be labeled and referred to as Table EV1, Dataset EV1, etc. Legends have to be provided in a separate tab in case of .xls files. Alternatively, the legend can be supplied as a separate text file (README) and zipped together with the Table/Dataset file.

See detailed instructions here:
.

10) The paper explained: EMBO Molecular Medicine articles are accompanied by a summary of the articles to emphasize the major findings in the paper and their medical implications for the non-specialist reader. Please provide a draft summary of your article highlighting
- the medical issue you are addressing,
- the results obtained and
- their clinical impact.

This may be edited to ensure that readers understand the significance and context of the research. Please refer to any of our published articles for an example.

11) For more information: There is space at the end of each article to list relevant web links for further consultation by our readers. Could you identify some relevant ones and provide such information as well? Some examples are patient associations, relevant databases, OMIM/proteins/genes links, author's websites, etc...

12) Author contributions: the contribution of every author must be detailed in a separate section (before the acknowledgments).

13) Conflict of interest: We updated our journal's competing interests policy in January 2022 and request authors to consider both actual and perceived competing interests. Please review the policy https://www.embopress.org/competing-interests and update your competing interests if necessary.

14) Every published paper now includes a 'Synopsis' to further enhance discoverability. Synopses are displayed on the journal webpage and are freely accessible to all readers. They include a short stand first (maximum of 300 characters, including space) as well as 2-5 one-sentences bullet points that summarizes the paper. Please write the bullet points to summarize the key NEW findings. They should be designed to be complementary to the abstract - i.e. not repeat the same text. We encourage inclusion of key acronyms and quantitative information (maximum of 30 words / bullet point). Please use the passive voice. Please attach these in a separate file or send them by email, we will incorporate them accordingly.

Please also suggest a striking image or visual abstract to illustrate your article as a PNG file 550 px wide x 300-600 px high.

15) As part of the EMBO Publications transparent editorial process initiative (see our Editorial at http://embomolmed.embopress.org/content/2/9/329), EMBO Molecular Medicine will publish online a Review Process File (RPF) to accompany accepted manuscripts.
In the event of acceptance, this file will be published in conjunction with your paper and will include the anonymous referee reports, your point-by-point response and all pertinent correspondence relating to the manuscript. Let us know whether you agree with the publication of the RPF and as here, if you want to remove or not any figures from it prior to publication.
Please note that the Authors checklist will be published at the end of the RPF.

EMBO Molecular Medicine has a "scooping protection" policy, whereby similar findings that are published by others during review or revision are not a criterion for rejection. Should you decide to submit a revised version, I do ask that you get in touch after three months if you have not completed it, to update us on the status.

I look forward to receiving your revised manuscript.

Yours sincerely,

Lise Roth

Lise Roth, PhD

Editor
EMBO Molecular Medicine

***** Reviewer's comments *****

Referee #1 (Comments on Novelty/Model System for Author):

The in vitro and in vivo model systems, patient samples and the statistical analysis authors used in the current study are adequate.

Referee #1 (Remarks for Author):

In the current manuscript, authors investigated the involvement of WD-repeat 6 protein in HCC. They have identified that WDR6 promotes HCC progression by enhancing Ub-dependent degradation of UVRAG tumor suppressor, hence fostering an immunosuppressive and pro-metastatic tumor microenvironment. Overall, it is an interesting study with novel findings, especially, identification of WDR6/UVRAG/NF-κB pathway in HCC. The experiments were logical and well connected. Authors performed a large number of in vitro experiments and good number of in vivo experiments which together support their conclusions. It is an interesting study and adds significant value and new information to the field of HCC.
Minor comments:

1. Figure 1A: It would be nice if the authors include a table showing the fold change of all the 7 genes so that the reader will have a clear picture of how WDR6 is expressed in relation to other 6 genes.
2. Figure 1B, lower bar graph: It would be easier for the reader if normal hepatocytes and metastatic tumor cells are shown in different color bars just like Figure 1Ci and Cii.
3. Page 8: "Since WDR6 knockdown did not change the mRNA levels of the five members (p65, RelB, c-Rel, p52, and p50) of the NF-κB family, but just downregulated the protein levels of p65 and pp65 (Fig. 5B)". I did not find the mRNA data in Fig 5.
4. Although, overall, the manuscript is relatively well written and logical, it has several grammatical mistakes and requires proof reading.

Referee #2 (Comments on Novelty/Model System for Author):

The use of both human and mouse HCC cell lines is good/ But authors did not use the same cell line model in all successive validation studies. This raised. the questiob whether observed findings are cell odel-dependent which limits general application of findings to HCC.

Referee #2 (Remarks for Author):

This paper is about the implications of WDR6 protein or WDxR peptide motif in hepatocellular carcinoma (HCC). WDR6 appears as a promoter of HCC progression by stimulating growth and metastasis via regulation of tumor microenvironment. Using human and mouse models of HCC\ the authors show that WDR6 overexpression in HCC allows favorable microenvironment by increasing MDSc in expense of CD8+ T cells. These cellular events are linked to WDR6 targeting of UVRAG to CUL4A-DDB1-ROC1 E3 ubiquitin ligase complex via WDxR motif and promotes its degradation. This blocks p65 degradation and activate TNFa expression leading to elevated MDSC accumulation. Inhibition of WDR6 by competitive cell=penenrating peptide increased the efficacy of anti-PDL1 treatment in mice.
Major comments:
1-authors used different in vitro and in vivo models by switching between models when testing successive hypotheses. A complete set of analysis using the same model would make this paper much better.
2-Cell-penetrating peptide treatment has major drawbacks for clinical use (specificity and stability issues). It is too early to expect that WDR6 can be a valid target in human HCC.
Minor comments:
1-The manuscript text, particularly supplementary material text requires English language editing.
2-Description of CCL4 treatment of mice is not clear.

Referee #3 (Comments on Novelty/Model System for Author):

First of all, WDR6, as a member of WD-repeat family, is rarely reported in tumors, which makes this research innovative. Secondly, the author used gene knockout model mice, which made the data more reliable. Finally, the author explored the WDxR-like peptide enhances the efficiency of anti-PD-1 against HCC, which is beneficial to clinical transformation.

Referee #3 (Remarks for Author):

The manuscript "Targeting WDxR motif reprograms immune microenvironment and inhibits hepatocellular carcinoma progression" presents the data about WDR6 can increase the levels of TNFα by stabilize the protein levels of p65, so as to recruits MDSCs and promotes hepatocellular carcinoma progression. The experiment data suggested that WDxR-like peptide enhances the efficiency of anti-PD-1 against HCC with WDR6 dysregulation. The author has made innovative research, but there are still the following problems to be solved.

1. Some other molecules in Figure 1A have higher fold change than WDR6. Please further explain the reason for choosing WDR6.
2. In Figure 2, there is no difference in tumor growth curve compared with the control by injecting shWDR6 cells into immunodeficiency mice, so is there any difference in tumor metastasis?
3. Please clarify which cell line or tissue was used to perform RNA-Seq in Figure 2E;
4. Please mark the number of animals in each group in Figure 3C
5. Please mark the comparison object corresponding to each asterisk in Figure 5A.
6. In Figure 5, the author proved that p65 acted as a transcription factor to regulate TNFa instead of phosphorylation. Please use corresponding experiments such as CHIP or double fluorescence to prove it.
7. In Figure 7B, the color of the right picture grouping label is inconsistent with the reality;
8. The content shown in Figure 8Ciii is inconsistent with the description in the result section, please check it.

Referee #1 (Comments on Novelty/Model System for Author):

The in vitro and in vivo model systems, patient samples and the statistical analysis authors used in the current study are adequate.

Referee #1 (Remarks for Author):

In the current manuscript, authors investigated the involvement of WD-repeat 6 protein in HCC. They have identified that WDR6 promotes HCC progression by enhancing Ub-dependent degradation of UVRAG tumor suppressor, hence fostering an immunosuppressive and pro-metastatic tumor microenvironment. Overall, it is an interesting study with novel findings, especially, identification of WDR6/UVRAG/NF-κB pathway in HCC. The experiments were logical and well connected. Authors performed a large number of in vitro experiments and good number of in vivo experiments which together support their conclusions. It is an interesting study and adds significant value and new information to the field of HCC.

Minor comments:

1. Figure 1A: It would be nice if the authors include a table showing the fold change of all the 7 genes so that the reader will have a clear picture of how WDR6 is expressed in relation to other 6 genes.

Response: Good suggestion! We included one table as Appendix Table S1.

2. Figure 1B, lower bar graph: It would be easier for the reader if normal hepatocytes and metastatic tumor cells are shown in different color bars just like Figure 1Ci and Cii.

Response: We updated Figure 1B as reviewer suggested.

3. Page 8: "Since WDR6 knockdown did not change the mRNA levels of the five members (p65, RelB, c-Rel, p52, and p50) of the NF-κB family, but just downregulated the protein levels of p65 and pp65 (Fig. 5B)". I did not find the mRNA data in Fig 5.

Response: We are sorry for this mistake. Please check our new Appendix Fig S2A.

4. Although, overall, the manuscript is relatively well written and logical, it has several grammatical mistakes and requires proof reading.

Response: Lots of thank Dr. Kavita Israni-Winger from Immunobiology Department of Yale University for proofreading this manuscript! We corrected the grammatical mistakes.

Referee #2 (Comments on Novelty/Model System for Author):

The use of both human and mouse HCC cell lines is good/ But authors did not use the

same cell line model in all successive validation studies. This raised. the questiob whether observed findings are cell odel-dependent which limits general application of findings to HCC.

Referee #2 (Remarks for Author):

This paper is about the implications of WDR6 protein or WDxR peptide motif in hepatocellular carcinoma (HCC). WDR6 appears as a promoter of HCC progression by stimulating growth and metastasis via regulation of tumor microenvironment. Using human and mouse models of HCC\ the authors show that WDR6 overexpression in HCC allows favorable microenvironment by increasing MDSc in expense of CD8+ T cells. These cellular events are linked to WDR6 targeting of UVRAG to CUL4A-DDB1-ROC1 E3 ubiquitin ligase complex via WDxR motif and promotes its degradation. This blocks p65 degradation and activate TNFa expression leading to elevated MDSC accumulation. Inhibition of WDR6 by competitive cell=penenrating peptide increased the efficacy of anti-PDL1 treatment in mice.
Major comments:
1-authors used different in vitro and in vivo models by switching between models when testing successive hypotheses. A complete set of analysis using the same model would make this paper much better.

Response: We completely understood the reviewer's comments. Thanks for this great suggestion! Given that we have already carried out in vivo experiments using two mouse cell lines H22 and Hepa1-6, we decided to use these two mouse cell lines to perform a complete set of in vitro analysis. In brief, we analyzed the effects of WDR6 knockdown (Hepa1-6 shWDR6) and overexpression (H22-WDR6) on several parameters and reorganized our manuscript:
1, cell growth in Appendix Fig S1C and D;
2, TNFα mRNA level and secretion in Fig 4Aii and B;
3, MDSC migration in Fig 4C;
Combined all, we provide a solid complete set of data using same model. We wish that performing these experiments can meet the requirement by reviewer. At the same time, we moved several previous data from human HCC cell lines to Appendix Figure S1.

2-Cell-penetrating peptide treatment has major drawbacks for clinical use (specificity and stability issues). It is too early to expect that WDR6 can be a valid target in human HCC.

Response: The reviewer is absolutely right! We totally agreed with the reviewer's comments about WDxR-like peptide. Cell penetrating peptides (CPPs), ideally 4–30 amino acids long, have been predominantly investigated for about twenty years. Due to their small size, high specificity, rapid renal clearance, reduced non-specific toxic effects, and efficient delivery to tumor, CPPs seem to have emerged as alternatives for monoclonal antibodies. A lot of CPP-based clinical trials have already been performed or are currently underway. Although *in vitro* and *in vivo* studies have reported promising

progression in design and application of CPPs, so far, no peptide or peptide conjugated drug for cancer therapeutic has received approval from US Food and Drug Administration (FDA). Therefore, it is of foremost importance to further improve the effects of CPPs on tumors, with reduced side-effect, higher specificity to the desired tissue or organ, increased stability, and enhanced therapeutic efficacy. In this study, we tried to use a WDxR peptide to treat HCC in mouse. However, we know that these data are very preliminary, and it is too early to expect that WDR6 can be a valid target in human HCC, as the reviewer points out. We included this description in the Discussion section.

Minor comments:
1-The manuscript text, particularly supplementary material text requires English language editing.

Response: We revised manuscript text, particularly supplementary material text as required for English language editing. Lots of thank Dr. Kavita Israni-Winger from Immunobiology Department of Yale University for proofreading this manuscript!

2-Description of CCL4 treatment of mice is not clear.

Response: We revised our description of CCL4 treatment in mice.

Referee #3 (Comments on Novelty/Model System for Author):

First of all, WDR6, as a member of WD-repeat family, is rarely reported in tumors, which makes this research innovative. Secondly, the author used gene knockout model mice, which made the data more reliable. Finally, the author explored the WDxR-like peptide enhances the efficiency of anti-PD-1 against HCC, which is beneficial to clinical transformation.

Referee #3 (Remarks for Author):

The manuscript "Targeting WDxR motif reprograms immune microenvironment and inhibits
hepatocellular carcinoma progression" presents the data about WDR6 can increase the levels of TNFα by stabilize the protein levels of p65, so as to recruits MDSCs and promotes hepatocellular carcinoma progression. The experiment data suggested that WDxR-like peptide enhances the efficiency of anti-PD-1 against HCC with WDR6 dysregulation. The author has made innovative research, but there are still the following problems to be solved.

1. Some other molecules in Figure 1A have higher fold change than WDR6. Please further explain the reason for choosing WDR6.

Response: Besides the reasons that we have stated, we focused on WDR6 for another two reasons: 1, WDR6 is rarely reported in tumors; 2, The dysregulation of other six genes has been reported to be associated with HCC pathogenesis. For example, we previously reported the effects of ATPIF1 on HCC (Song, Song et al., 2014b, PMID: 25042864). We added this description into the Results section.

2. In Figure 2, there is no difference in tumor growth curve compared with the control by injecting shWDR6 cells into immunodeficiency mice, so is there any difference in tumor metastasis?

Response: Compared with the control, there is not any difference in tumor metastasis by injecting shWDR6 cells into immunodeficiency mice.

3. Please clarify which cell line or tissue was used to perform RNA-Seq in Figure 2E;

Response: We used HCC patient tumor tissues (two groups: WDR6 high & WDR6 low) which were subjected to RNA-seq in Figure 2E.

4. Please mark the number of animals in each group in Figure 3C.

Response: We marked the number of animals in each group in Figure 3C.

5. Please mark the comparison object corresponding to each asterisk in Figure 5A.

Response: Thanks for this suggestion! We marked the comparison object corresponding to each asterisk in Figure 5A.

6. In Figure 5, the author proved that p65 acted as a transcription factor to regulate TNFa instead of phosphorylation. Please use corresponding experiments such as CHIP or double fluorescence to prove it.

Response: We included CHIP. Please check our Appendix Fig S2B.

7. In Figure 7B, the color of the right picture grouping label is inconsistent with the reality;

Response: We corrected this mistake in Figure 7B. Thanks for this suggestion!

8. The content shown in Figure 8Ciii is inconsistent with the description in the result section, please check it.

Response: We are sorry for this mistake! Thanks a lot! We corrected this mistake in figure legends.

15th Jul 2022

Dear Prof. Zhang,

Thank you for submitting your revised manuscript to EMBO Molecular Medicine.
We have now received the report from referee #2 who re-reviewed your manuscript. As you will see, while this referee is satisfied with your responses to the initial concerns, he/she also raises a key issue regarding the choice of antibody to study PD1 inhibition.
Therefore, we would like you to address this referee's concern in a new revised version of your manuscript.

Additionally, please also address the following editorial issues:

- Please address the queries from our data editors (in Data edited MS file) and use this file for any further modification. Please remove the yellow highlights and keep in track changes mode any new change. Please also remove the yellow highlights in the Appendix file.
- We note that you currently have 3 first-authors. Can you confirm that this is correct, and that these 3 authors contributed equally to the work?
- Data Availability Section: Please note that all datasets should be made public before acceptance of your manuscript.
- We would also encourage you to include the source data for figure panels that show essential data. Numerical data should be provided as individual .xls or .csv files (including a tab describing the data). For blots or microscopy, uncropped images should be submitted (using a zip archive if multiple images need to be supplied for one panel). In particular, please clarify the similarities between Figure 4Ai and 7Dii. If images are re-used in several figures, it should be clearly stated in the figure legends.
- Please provide in your figures or in their legends the exact p values, not a range, including for non-significant (n.s.) values.
- Thank you for providing a synopsis picture. Please upload it as a PNG/TIFF/JPEG file 550px wide x 300-600 px high.
- As part of the EMBO Publications transparent editorial process initiative (see our Editorial at http://embomolmed.embopress.org/content/2/9/329), EMBO Molecular Medicine will publish online a Review Process File (RPF) to accompany accepted manuscripts.
This file will be published in conjunction with your paper and will include the anonymous referee reports, your point-by-point response and all pertinent correspondence relating to the manuscript. Let us know whether you agree with the publication of the RPF and as here, if you want to remove or not any figures from it prior to publication.
Please note that the Authors checklist will be published at the end of the RPF.

I look forward to receiving your revised manuscript.

With kind regards,

Lise Roth

Lise Roth, PhD
Senior Editor
EMBO Molecular Medicine

***** Reviewer's comments *****

Referee #2 (Comments on Novelty/Model System for Author):

There is a problem in the choice of antibody to study the effect of PD-1 inhibition in animal model of HCC.

Referee #2 (Remarks for Author):

The authors addressed adequately my concerns in the review of the first manuscript.
Major comment:
Unfortunately, the following key issue came into my attention during the review of the revised manuscript: On page 21 line 3, the authors stated than 'BALB/c mouse was injected via i.p. using vehicle or Pep-WDxR, with or without PD-1 antibody (10F.9G2, Bio X Cell, 2 mg/kg) or IgG antibody (4 mg/kg), respectively'. Furthermore, the headline of the result section related to immunotherapy stated A WDxR-like peptide increases the efficacy of anti-PD-1 therapy in HCC treatment'. Accordingly, the legend of Fig. 8 states 'Fig. 8. A WDxR-like peptide increases the efficacy of anti-PD-1 antibody against HCC. 'PD-1 ab'

qualification was used several times in Fig.8 illustrating in vivo tumor assays.

!0F.9G2 is a rat monoclonal antibody directed to mouse PDL-1, but not PD-1 (Dahan et al. Cancer Cell, 28: 285-295, 2015). Thus, in contrast to the authors repeated statements in the paper, they have targeted PDL-1 but nor PD-1. This may have important implications as PDL-1 is a PD-1 ligand diverted by some cancer cells, whereas PD-1 is expressed by T cells as a receptor involved in immune checkpoint control. I believe this situation does not compromise the validity of the data reporting the role of WDR6 in HCC, but may create a problem for therapies combining WDxR-like peptide and anti PD-1 (or anti-PDL-1) antibody. Although both antibodies act mainly to disrupt PD1-PDL1 binding, their mechanism of action may differ, as suggested for example by Dahan et al. (Dahan et al. Cancer Cell, 28: 285-295, 2015). The authors need to provide clarifications about the issues raised and need to revise their manuscript carefully to make necessary changes in different parts including the abstract.

15th Jul 2022

Dear Prof. Zhang,

Thank you for submitting your revised manuscript to EMBO Molecular Medicine.
We have now received the report from referee #2 who re-reviewed your manuscript. As you will see, while this referee is satisfied with your responses to the initial concerns, he/she also raises a key issue regarding the choice of antibody to study PD1 inhibition.
Therefore, we would like you to address this referee's concern in a new revised version of your manuscript.

Response: Yes, we addressed the 2# referee's concern.

Additionally, please also address the following editorial issues:

- Please address the queries from our data editors (in Data edited MS file) and use this file for any further modification. Please remove the yellow highlights and keep in track changes mode any new change. Please also remove the yellow highlights in the Appendix file.
- We note that you currently have 3 first-authors. Can you confirm that this is correct, and that these 3 authors contributed equally to the work?

Response: Yes, I confirm that these authors contributed equally to the work.

- Data Availability Section: Please note that all datasets should be made public before acceptance of your manuscript.

Response: Yes, all datasets were made public in the first revision.

- We would also encourage you to include the source data for figure panels that show essential data. Numerical data should be provided as individual .xls or .csv files (including a tab describing the data). For blots or microscopy, uncropped images should be submitted (using a zip archive if multiple images need to be supplied for one panel). In particular, please clarify the similarities between Figure 4Ai and 7Dii. If images are re-used in several figures, it should be clearly stated in the figure legends.

Response: Yes, we included the source data for all figure panels. For the similarities between Figure 4Ai and Figure 7Dii, we clarified it in Figure legend: Although these three groups were compared, the results needed to be presented in different figures.

- Please provide in your figures or in their legends the exact p values, not a range, including for non-significant (n.s.) values.

Response: Yes, we provided the exact p values in Figures or their legends, including non-significant (n.s.) values.

- Thank you for providing a synopsis picture. Please upload it as a PNG/TIFF/JPEG file 550px wide x 300-600 px high.

Response: Yes, we uploaded the synopsis picture as a PNG/TIFF/JPEG file 550px wide x 300-600 px high.

- As part of the EMBO Publications transparent editorial process initiative (see our Editorial at http://embomolmed.embopress.org/content/2/9/329), EMBO Molecular Medicine will publish online a Review Process File (RPF) to accompany accepted manuscripts.
This file will be published in conjunction with your paper and will include the anonymous referee reports, your point-by-point response and all pertinent correspondence relating to the manuscript. Let us know whether you agree with the publication of the RPF and as here, if you want to remove or not any figures from it prior to publication.
Please note that the Authors checklist will be published at the end of the RPF.

Response: Yes, we agree with the publication of the RPF.

I look forward to receiving your revised manuscript.

With kind regards,

Lise Roth

Lise Roth, PhD
Senior Editor
EMBO Molecular Medicine

***** Reviewer's comments *****

Referee #2 (Comments on Novelty/Model System for Author):

There is a problem in the choice of antibody to study the effect of PD-1 inhibition in animal model of HCC.

Referee #2 (Remarks for Author):

The authors addressed adequately my concerns in the review of the first manuscript.
Major comment:
Unfortunately, the following key issue came into my attention during the review of the revised manuscript: On page 21 line 3, the authors stated than 'BALB/c mouse was injected via i.p. using vehicle or Pep-WDxR, with or without PD-1 antibody (10F.9G2, Bio X Cell, 2 mg/kg) or IgG antibody (4 mg/kg), respectively'. Furthermore, the headline of the result section related to immunotherapy stated A WDxR-like peptide increases the efficacy of anti-PD-1 therapy in HCC treatment'. Accordingly, the legend of Fig. 8 states 'Fig. 8. A WDxR-like peptide increases the efficacy of anti-PD-1 antibody against HCC. 'PD-1 ab' qualification was used several times in Fig.8 illustrating in vivo tumor assays.

!0F.9G2 is a rat monoclonal antibody directed to mouse PDL-1, but not PD-1 (Dahan et al. Cancer Cell, 28: 285-295, 2015). Thus, in contrast to the authors repeated statements in the paper, they have targeted PDL-1 but nor PD-1. This may have important implications as PDL-1 is a PD-1 ligand diverted by some cancer cells, whereas PD-1 is expressed by T cells as a receptor involved in immune checkpoint control. I believe this situation does not compromise the validity of the data reporting the role of WDR6 in HCC, but may create a problem for therapies combining WDxR-like peptide and anti PD-1 (or anti-PDL-1) antibody. Although both antibodies act mainly to disrupt PD1-PDL1 binding, their mechanism of action may differ, as suggested for example by Dahan et al. (Dahan et al. Cancer Cell, 28: 285-295, 2015). The authors need to provide clarifications about the issues raised and need to revise their manuscript carefully to make necessary changes in different parts including the abstract.

Response: Many thanks for the referee's comment! And this referee was so nice and pointed to every error in the manuscript, which greatly improve the manuscript. We also carefully checked and repeated experiments. Based on the referee's suggestion, we revised the manuscript and made necessary changes including the abstract.

12th Jan 2023

Dear Prof. Zhang,

Thank you for submitting your revised manuscript to EMBO Molecular Medicine, and please accept my apologies for the delay in getting back to you during this busy time of the year. We have now received the report from referee #2 who re-reviewed your manuscript. As you will see below, this referee is now supportive of publication, and I am therefore pleased to inform you that we will be able to accept your manuscript once the following editorial points will be addressed:

1/ Manuscript text:
- Materials and Methods:
o Please indicate the origin of the mice, and the formula used to measure the tumors.
o Please provide the antibody concentrations.
o Please include a statement that informed consent was obtained from all subjects and that the experiments conformed to the principles set out in the WMA Declaration of Helsinki and the Department of Health and Human Services Belmont Report.
o Please indicate whether the cells were tested for mycoplasma contamination and were authenticated (when applicable).
o In the statistic section, please provide information on exclusion criteria, blinding and randomization.
Moreover, please carefully check again the language and grammar of this section.
- Data Availability Section: Please remove "All other data are available from the corresponding author on request." We note that the dataset PXD034521 is still private and should be made public before acceptance.
- Author contributions: CRediT has replaced the traditional author contributions section because it offers a systematic machine readable author contributions format that allows for more effective research assessment. Please remove the Authors Contributions from the manuscript and use the free text boxes beneath each contributing author's name in our system to add specific details on the author's contribution. More information is available in our guide to authors.

2/ Source data:
Thank you for providing Source Data. Please carefully check them again, in particular the source data for figure 7Aii, 7Aiii, and Appendix Fig. S2Ci.

3/ Checklist
- Cell lines: please provide information regarding mycoplasma contamination and authentication.
- You filled the section "animal observed in or captured from the field" but I do not think this section is applicable to your study, please check.

4/ Thank you for providing "The paper explained". I added minor modifications and comments, please adjust as you see fit.
Problem
The bad prognosis and high mortality of hepatocellular carcinoma (HCC) are ascribed to a high recurrence rate post-resection and the absence of early diagnostic tools. We investigated the role of WDR6 in HCC. (please add a sentence to explain why)
Results
WDR6 deficiency inhibited HCC growth and metastasis in immune-competent mice only. WDR6 degraded UVRAG by recruiting CUL4A-DDB1-ROC1 E3 ligase complex through its unique WDxR motif, which upregulated p65-induced TNFα, elevated intra-tumoral MDSC number, and reduced CD8+ T cell infiltration, thereby promoting HCC progression. These immunosuppressive effects were reversed by TNFα blockade. TNFα in turn recruited NF-κB to activate WDR6 transcription, establishing a WDR6-TNFα loop. Clinically, the WDR6/UVRAG/NF-κB pathway was hyper-activated in HCC, and predicted a poor prognosis. Importantly, a WDxR-like peptide enhanced the efficacy of anti-PD-L1 against HCC.
Impact
Our findings demonstrate the therapeutic potential of immune-modulation to suppress HCC with WDR6 dysregulation.

5/ Thank you for providing a nice synopsis. I slightly modified your text, please let me know if you agree with the following or amend as you see fit:

The WD-repeat (WDR) family affects carcinogenesis, but its role in immune microenvironment is poorly characterized. This study reports the cellular and molecular effects of targeting WDR6 on HCC.
- Functional loss of WDR6 inhibited HCC growth and metastasis only in immune-competent mice
- Increased number of intra-tumoral PMN-MDSCs mediated by WDR6 activation was reversed by TNFα blockade
- UVRAG degradation was enhanced by WDR6 via a WDxR motif, thereby leading to TNFα increase and PMN-MDSC recruitment in HCC.
- Anti-PD-L1 immunotherapy efficiency was improved by a WDxR-like peptide in HCC with WDR6 dysregulation.

We note that you agree with the publication of the Review Process File.

I look forward to receiving your revised manuscript.

Yours sincerely,

Lise Roth
Lise Roth, PhD
Senior Editor
EMBO Molecular Medicine

The system will prompt you to fill in your funding and payment information. This will allow Wiley to send you a quote for the article processing charge (APC) in case of acceptance. This quote takes into account any reduction or fee waivers that you may be eligible for. Authors do not need to pay any fees before their manuscript is accepted and transferred to our publisher.

***** Reviewer's comments *****

Referee #2 (Remarks for Author):

The revised manuscript adequately addressed the last issue (PDL1 antibody) remaining for the acceptance of this paper.

The authors addressed the editorial requests.

28th Feb 2023

Dear Dr. Zhang,

Thank you for submitting your revised manuscript. I am pleased to inform you that your manuscript is accepted for publication and is now being sent to our publisher to be included in the next available issue of EMBO Molecular Medicine!

We would like to remind you that as part of the EMBO Publications transparent editorial process initiative, EMBO Molecular Medicine will publish a Review Process File online to accompany accepted manuscripts.

To better promote your work among the Chinese readership, we would like to invite you to prepare a short summary of the manuscript in Chinese, which we will promote on the WeChat platform 'BioArt' with 240,000 followers.

If you are interested in taking up this opportunity, we recommend to cover the article very close to its online publication date. Thus, ideally we will very much appreciate it if you can send us a draft by replying to this invitation email within the next 7 working days. My colleague Jingyi Hou will be happy to suggest any edits that may help to further improve the text. Please let us know within 3 days if you plan (or not) to contribute such a short summary in Chinese.

Below please find some general guidelines on how to prepare a summary. I have also included links to recent examples for your reference.

Congratulations on your interesting work!

Sincerely,

Lise Roth

Lise Roth, Ph.D
Senior Editor
EMBO Molecular Medicine

Follow us on Twitter @EmboMolMed
Sign up for eTOCs at embopress.org/alertsfeeds

General Guidelines for Chinese summary:

• These summary articles are meant to be targeting general audience so please limit the use of specialized technical terms, acronyms and jargon.
• A summary usually starts with brief background information of the reported work, which is followed by explaining the findings in some detail, and ends with a short review of the conclusions as well as the implications of the work and future directions for the research.
• The summary should at least contain one graphical item, such as a scheme or a figure from the paper.
• Please provide ONE SINGLE document containing all text and graphical materials, ideally as a Word.docx or .doc file. Please DO NOT provide the document as a .pdf file.
• Please DO NOT publicly release the document before the paper is officially published online.

Summary Examples

https://mp.weixin.qq.com/s/kF2fOWvJRKrDi0ap90IvIA

EMBO J | 罗招庆/欧阳松应揭示谷酰胺脱氨酶MvcA的去泛素化功能

EMBO J丨王松灵院士团队揭示组织内应力调控大型哺乳动物乳恒牙替换的新机制
"炎"或"焱"？--曾木圣团队等报道酪氨酸激酶EphA2抑制RNA病毒对呼吸道NLRP3炎症小体通路过度激活及肺损伤
研究者鉴定了EphA2作为NLRP3的负性调节因子，对理解炎症过度活化的调控和人类自身免疫性疾病的发病机制及研发相应的干预手段具有重要意义。
mp.weixin.qq.com

*** *** *** IMPORTANT INFORMATION *** *** ***

SPEED OF PUBLICATION
The journal aims for rapid publication of papers, using using the advance online publication "Early View" to expedite the process: A properly copy-edited and formatted version will be published as "Early View" after the proofs have been corrected. Please help the Editors and publisher avoid delays by providing e-mail address(es), telephone and fax numbers at which author(s) can be contacted.

Should you be planning a Press Release on your article, please get in contact with embomolmed@wiley.com as early as possible, in order to coordinate publication and release dates.

LICENSE AND PAYMENT:

All articles published in EMBO Molecular Medicine are fully open access: immediately and freely available to read, download and share.

EMBO Molecular Medicine charges an article processing charge (APC) to cover the publication costs. You, as the corresponding author for this manuscript, should have already received a quote with the article processing fee separately. Please let us know in case this quote has not been received.

Once your article is at Wiley for editorial production you will receive an email from Wiley's Author Services system, which will ask you to log in and will present you with the publication license form for completion. Within the same system the publication fee can be paid by credit card, an invoice, pro forma invoice or purchase order can be requested.

Payment of the publication charge and the signed Open Access Agreement form must be received before the article can be published online.

PROOFS

You will receive the proofs by e-mail approximately 2 weeks after all relevant files have been sent o our Production Office. Please return them within 48 hours and if there should be any problems, please contact the production office at embopressproduction@wiley.com.

Please inform us if there is likely to be any difficulty in reaching you at the above address at that time. Failure to meet our deadlines may result in a delay of publication.

All further communications concerning your paper proofs should quote reference number EMM-2022-15924-V4 and be directed to the production office at embopressproduction@wiley.com.

EMBO Press Author Checklist

Corresponding Author Name: Zhiyong Zhang
Journal Submitted to: EMBO Molecular Medicine
Manuscript Number: EMM-2022-15924

USEFUL LINKS FOR COMPLETING THIS FORM
The EMBO Journal - Author Guidelines
EMBO Reports - Author Guidelines
Molecular Systems Biology - Author Guidelines
EMBO Molecular Medicine - Author Guidelines

Reporting Checklist for Life Science Articles (updated January 2022)
This checklist is adapted from Materials Design Analysis Reporting (MDAR) Checklist for Authors. MDAR establishes a minimum set of requirements in transparent reporting in the life sciences (see Statement of Task: 10.31222/osf.io/9sm4x). Please follow the journal's guidelines in preparing your manuscript.
Please note that a copy of this checklist will be published alongside your article.

Abridged guidelines for figures
1. Data
The data shown in figures should satisfy the following conditions:
- the data were obtained and processed according to the field's best practice and are presented to reflect the results of the experiments in an accurate and unbiased manner.
- ideally, figure panels should include only measurements that are directly comparable to each other and obtained with the same assay.
- plots include clearly labeled error bars for independent experiments and sample sizes. Unless justified, error bars should not be shown for technical replicates.
- if n<5, the individual data points from each experiment should be plotted. Any statistical test employed should be justified.
- Source Data should be included to report the data underlying figures according to the guidelines set out in the authorship guidelines on Data Presentation.

2. Captions
Each figure caption should contain the following information, for each panel where they are relevant:
- a specification of the experimental system investigated (eg cell line, species name).
- the assay(s) and method(s) used to carry out the reported observations and measurements.
- an explicit mention of the biological and chemical entity(ies) that are being measured.
- an explicit mention of the biological and chemical entity(ies) that are altered/varied/perturbed in a controlled manner.
- the exact sample size (n) for each experimental group/condition, given as a number, not a range;
- a description of the sample collection allowing the reader to understand whether the samples represent technical or biological replicates (including how many animals, litters, cultures, etc.).
- a statement of how many times the experiment shown was independently replicated in the laboratory.
- definitions of statistical methods and measures:
  - common tests, such as t-test (please specify whether paired vs. unpaired), simple χ2 tests, Wilcoxon and Mann-Whitney tests, can be unambiguously identified by name only, but more complex techniques should be described in the methods section;
  - are tests one-sided or two-sided?
  - are there adjustments for multiple comparisons?
  - exact statistical test results, e.g., P values = x but not P values < x;
  - definition of 'center values' as median or average;
  - definition of error bars as s.d. or s.e.m.

Please complete ALL of the questions below.
Select "Not Applicable" only when the requested information is not relevant for your study.

Materials

Newly Created Materials	Information included in the manuscript?	In which section is the information available? (Reagents and Tools Table, Materials and Methods, Figures, Data Availability Section)
New materials and reagents need to be available; do any restrictions apply?	Not Applicable	

Antibodies	Information included in the manuscript?	In which section is the information available? (Reagents and Tools Table, Materials and Methods, Figures, Data Availability Section)
For **antibodies** provide the following information:<br>- Commercial antibodies: RRID (if possible) or supplier name, catalogue number and or/clone number<br>- Non-commercial: RRID or citation	Yes	Materials and Methods

DNA and RNA sequences	Information included in the manuscript?	In which section is the information available? (Reagents and Tools Table, Materials and Methods, Figures, Data Availability Section)
**Short novel DNA or RNA including primers, probes**: provide the sequences.	Yes	Materials and Methods

Cell materials	Information included in the manuscript?	In which section is the information available? (Reagents and Tools Table, Materials and Methods, Figures, Data Availability Section)
**Cell lines:** Provide species information, strain. Provide accession number in repository **OR** supplier name, catalog number, clone number, and/**OR** RRID.	Yes	Materials and Methods
**Primary cultures:** Provide species, strain, sex of origin, genetic modification status.	Yes	Materials and Methods
Report if the cell lines were recently **authenticated** (e.g., by STR profiling) and tested for mycoplasma contamination.	Yes	Materials and Methods

Experimental animals	Information included in the manuscript?	In which section is the information available? (Reagents and Tools Table, Materials and Methods, Figures, Data Availability Section)
**Laboratory animals or Model organisms:** Provide species, strain, sex, age, genetic modification status. Provide accession number in repository **OR** supplier name, catalog number, clone number, **OR** RRID.	Yes	Materials and Methods
**Animal observed in or captured from the field:** Provide species, sex, and age where possible.	Not Applicable	
Please detail **housing and husbandry conditions**.	Yes	Materials and Methods

Plants and microbes	Information included in the manuscript?	In which section is the information available? (Reagents and Tools Table, Materials and Methods, Figures, Data Availability Section)
**Plants:** provide species and strain, ecotype and cultivar where relevant, unique accession number if available, and source (including location for collected wild specimens).	Not Applicable	
**Microbes:** provide species and strain, unique accession number if available, and source.	Not Applicable	

Human research participants	Information included in the manuscript?	In which section is the information available? (Reagents and Tools Table, Materials and Methods, Figures, Data Availability Section)
If collected and within the bounds of privacy constraints report on age, sex and gender or ethnicity for all study participants.	Not Applicable	

Core facilities	Information included in the manuscript?	In which section is the information available? (Reagents and Tools Table, Materials and Methods, Figures, Data Availability Section)
If your work benefited from core facilities, was their service mentioned in the acknowledgments section?	Not Applicable	

Design

Study protocol	Information included in the manuscript?	In which section is the information available? (Reagents and Tools Table, Materials and Methods, Figures, Data Availability Section)
If study protocol has been **pre-registered, provide DOI in the manuscript**. For clinical trials, provide the trial registration number **OR** cite DOI.	Not Applicable	
Report the **clinical trial registration number** (at ClinicalTrials.gov or equivalent), where applicable.	Not Applicable	

Laboratory protocol	Information included in the manuscript?	In which section is the information available? (Reagents and Tools Table, Materials and Methods, Figures, Data Availability Section)
Provide DOI OR other citation details if **external detailed step-by-step protocols** are available.	Not Applicable	

Experimental study design and statistics	Information included in the manuscript?	In which section is the information available? (Reagents and Tools Table, Materials and Methods, Figures, Data Availability Section)
Include a statement about **sample size** estimate even if no statistical methods were used.	Yes	Figures
Were any steps taken to minimize the effects of subjective bias when allocating animals/samples to treatment (e.g. **randomization procedure**)? If yes, have they been described?	Yes	Figures
Include a statement about **blinding** even if no blinding was done.	Yes	Materials and Methods
Describe **inclusion/exclusion criteria** if samples or animals were excluded from the analysis. Were the criteria pre-established?<br><br>If sample or data points were omitted from analysis, report if this was due to attrition or intentional exclusion and provide justification.	Not Applicable	
For every figure, are **statistical tests** justified as appropriate? Do the data meet the assumptions of the tests (e.g., normal distribution)? Describe any methods used to assess it. Is there an estimate of variation within each group of data? Is the variance similar between the groups that are being statistically compared?	Yes	Figures<br>Materials and Methods

Sample definition and in-laboratory replication	Information included in the manuscript?	In which section is the information available? (Reagents and Tools Table, Materials and Methods, Figures, Data Availability Section)
In the figure legends: state number of times the experiment was **replicated** in laboratory.	Yes	Figures
In the figure legends: define whether data describe **technical or biological replicates**.	Yes	Figures

Ethics

Ethics	Information included in the manuscript?	In which section is the information available? (Reagents and Tools Table, Materials and Methods, Figures, Data Availability Section)
Studies involving **human participants**: State details of **authority granting ethics approval** (IRB or equivalent committee(s), provide reference number for approval.	Yes	Materials and Methods
Studies involving **human participants**: Include a statement confirming that **informed consent** was obtained from all subjects and that the experiments conformed to the principles set out in the WMA Declaration of Helsinki and the Department of Health and Human Services Belmont Report.	Yes	Materials and Methods
Studies involving **human participants:** For publication of **patient photos**, include a statement confirming that consent to publish was obtained.	Not Applicable	
Studies involving experimental **animals**: State details of **authority granting ethics approval** (IRB or equivalent committee(s), provide reference number for approval. Include a statement of compliance with ethical regulations.	Yes	Materials and Methods
Studies involving **specimen and field samples:** State if relevant **permits** obtained, provide details of authority approving study; if none were required, explain why.	Not Applicable	

Dual Use Research of Concern (DURC)	Information included in the manuscript?	In which section is the information available? (Reagents and Tools Table, Materials and Methods, Figures, Data Availability Section)
Could your study fall under dual use research restrictions? Please check biosecurity documents and list of **select agents and toxins** (CDC): https://www.selectagents.gov/sat/list.htm	Not Applicable	
If you used a select agent, is the security level of the lab appropriate and reported in the manuscript?	Not Applicable	
If a study is subject to dual use research of concern regulations, is the name of the **authority granting approval and reference number** for the regulatory approval provided in the manuscript?	Not Applicable	

Reporting

The MDAR framework recommends adoption of discipline-specific guidelines, established and endorsed through community initiatives. Journals have their own policy about requiring specific guidelines and recommendations to complement MDAR.

Adherence to community standards	Information included in the manuscript?	In which section is the information available? (Reagents and Tools Table, Materials and Methods, Figures, Data Availability Section)
State if relevant guidelines or checklists (e.g., **ICMJE, MIBBI, ARRIVE, PRISMA**) have been followed or provided.	Not Applicable	
For **tumor marker prognostic studies,** we recommend that you follow the **REMARK** reporting guidelines (see link list at top right). See author guidelines, under 'Reporting Guidelines'. Please confirm you have followed these guidelines.	Not Applicable	
For **phase II and III randomized controlled trials**, please refer to the **CONSORT** flow diagram (see link list at top right) and submit the CONSORT checklist (see link list at top right) with your submission. See author guidelines, under 'Reporting Guidelines'. Please confirm you have submitted this list.	Not Applicable	

Data Availability

Data availability	Information included in the manuscript?	In which section is the information available? (Reagents and Tools Table, Materials and Methods, Figures, Data Availability Section)
Have **primary datasets** been deposited according to the journal's guidelines (see 'Data Deposition' section) and the respective accession numbers provided in the Data Availability Section?	Yes	Data Availability
Were **human clinical and genomic datasets** deposited in a public access-controlled repository in accordance to ethical obligations to the patients and to the applicable consent agreement?	Not Applicable	
Are **computational models** that are central and integral to a study available without restrictions in a machine-readable form? Were the relevant accession numbers or links provided?	Not Applicable	
If publicly available data were reused, provide the respective **data citations in the reference list**.	Not Applicable	
